# EXACT PHYLODYNAMIC LIKELIHOOD VIA STRUCTURED MARKOV GENEALOGY PROCESSES

**Published:** 2025-01-16

**Authors:** AARON A. KING, QIANYING LIN, EDWARD L. IONIDES

## Abstract

We consider genealogies arising from a Markov population process in which individuals are categorized into a discrete collection of compartments, with the requirement that individuals within the same compartment are statistically exchangeable. When equipped with a sampling process, each such population process induces a time-evolving tree-valued process defined as the genealogy of all sampled individuals. We provide a construction of this genealogy process and derive exact expressions for the likelihood of an observed genealogy in terms of filter equations. These filter equations can be numerically solved using standard Monte Carlo integration methods. Thus, we obtain statistically efficient likelihood-based inference for essentially arbitrary compartment models based on an observed genealogy of individuals sampled from the population.

## 1. Introduction.

When the genome of an infectious agent accumulates mutations on timescales similar to those of transmission and infection progression, the resulting pattern of differences among genomes contains information on the history of the pathogen’s passage through individual hosts and the host population. As Grenfell et al. (2004) observed, one can extract this information to gain insight into the structure and dynamics of the host-pathogen system. In particular, one can formalize mathematical models of transmission, estimate their parameters, and compare their ability to explain data, following standard statistical paradigms. This is known as *phylodynamic* inference. Alizon (2024) gives a good review of the history of the subject.

The most common approach to phylodynamic inference rests upon a mathematical linkage between the tree-like *genealogy* or *phylogeny* that expresses the relationships of shared ancestry among sampled genomes and a model of the dynamics of the transmission system. Various linkages are possible, but because it is maximally efficient (i.e., loses the least information), it is desirable to be able to compute the likelihood function for models of interest. This is simply the probability density of a given genealogy conditional on a given model, viewed as a function of the parameters of that model. In particular, if S is a set of genome sequences, Φ a genealogical tree relating these sequences, E a model of sequence evolution, and D a dynamic transmission model, then the likelihood is

𝓛(D,E)=f(S∣D,E)=∫f(S∣Φ,E)f(Φ∣D)dΦ,

where the integral is taken over all possible genealogies and we somewhat loosely use the symbol f for the various distinct probability densities, the nature of each of which is clear from its arguments. In this expression, f(S∣Φ,E) is typically the Felsenstein (2004) phylogenetic likelihood. The function f(Φ∣D), which links the phylogeny to the dynamic model, may be termed the *phylodynamic likelihood*. In the Bayesian context, this same function is sometimes referred to as a *tree prior* (Möller et al., 2018; Volz & Siveroni, 2018). The computation of this function has remained out of reach, except in several special cases. This paper presents theory that enables its computation for a very broad range of dynamic models.

Existing approaches to the phylodynamic likelihood have been based on one of two mathematical idealizations. The first is the Kingman (1982a) coalescent, by which likelihood of a given genealogy is computed using a reverse-time argument. This computation provides the exact likelihood for a genealogy resulting from a particular, constant population-size, dynamic model (the Moran model, e.g., Moran, 1958; Kingman, 1982b; Möhle, 2000). Extensions of this approach develop approximate likelihoods for the case when the population size varies as a function of time (Griffiths & Tavaré, 1994; Drummond et al., 2005) or according to an SIR process (Volz et al., 2009; Rasmussen et al., 2011), as long as the population size is large and the sample-fraction remains negligible. The second idealization is the linear birth-death process, for which exact expressions for the likelihood are available (Stadler, 2010). Linearity in this context amounts to the assumption that distinct lineages do not interact: it is the resulting self-similarity of genealogies that renders the likelihood analytically tractable. Extensions of this approach develop approximations via linearization of nonlinear processes or restriction to scenarios in which population growth is nearly linear (e.g., MacPherson et al., 2021). Although the tractability of these approaches makes them attractive, concern naturally arises as to validity of the approximations in specific cases, the biases introduced by them, and the amount of information in data left unutilized by these approximate methods. For this reason, there is interest in improved phylodynamic inference techniques.

What would an ideal phylodynamic inference method look like? First, it would afford exact computation of the phylodynamic likelihood, so that comparisons among parameterizations and models could be made on a sound basis. Second, because nonlinearity, nonstationarity, noise, and measurement error are prominent and ubiquitous in epidemiology, it would accommodate nonlinear, time-inhomogeneous, stochastic transmission models. Third, because many of the most scientifically important uncertainties concern heterogeneities in transmission rates and the susceptibility, behavior, age, and location of hosts, it would accommodate host populations structured by these factors. While some structuring factors (e.g., age, spatial location) are most naturally expressed in terms of continuous variables, discretely structured models have repeatedly proved their value in epidemiology. In particular, compartmental models are extremely flexible and have often been used as approximations when continuous structure leads to uncomfortably high model dimension. Finally, because there is typically uncertainty not only in the parameters, but also in the structure, of a host-pathogen system, an improved phylodynamic inference methodology would place minimal restrictions on the form of the models that it can accommodate. This paper demonstrates how these desiderata can be achieved—at least for models with discrete structure—including arbitrary nonlinear compartmental models.

Of course, practical considerations play an important role as well. In practice, data availability typically places strong limits on the degree of model complexity that can be supported by data. In addition, computational expense typically grows with model complexity and this can also limit the utility of otherwise attractive models and inference methods. Nevertheless, in the present paper we confine ourselves to theoretical considerations. The results we present could form the basis for a variety of distinct algorithms the relative value of which will depend on the questions asked, models proposed, and data available, and in any case remains to be seen. Moreover, although the theory we present is valid for models with even a countably infinite number of compartments, lack of data and computational resources will in practice require that the models that can effectively be employed may be much simpler than desired.

To connect a model at the level of a population with genealogies based on samples taken from individual hosts, it is necessary to make assumptions about the individuals in the population. The simplest such assumption is that the individuals that are identical with respect to the population dynamics are indeed statistically identical. That is, that they are *exchangeable*. In a compartmental model, this is tantamount to the assumption that the residence times of the individuals within each compartment are identically distributed, though not independent. Although exchangeability is indeed an additional assumption, it is so natural that it is frequently unrecognized as such, and one often reads statements to the effect that exchangeability of individuals is a consequence of the Markovian assumption. Nonetheless, since it adds minimal additional structure, it is the natural assumption, and the one we will make in this paper.

In the following, we take as our starting point a transmission model in the form of a discretely structured, Markov process. We show how such a process uniquely induces each of several stochastic processes in the space of genealogies. We go on to derive expressions for the exact likelihoods of these genealogies.

Code sufficient for the reproduction of all the results presented in this paper are freely available for download at https://github.com/kingaa/structured-genealogy-process-paper. An archival version of these will be stored on Zotero upon publication of a peer-reviewed version of this paper. The open-source **R** package **phylopomp** (https://github.com/kingaa/phylopomp) implements the simulation and likelihood-computation algorithms employed here.

## 2. Mathematical preliminaries.

### 2.1. Notation.

Throughout the paper, we will adopt the convention that a bold-face symbol (e.g., X), denotes a random element. We will be concerned with a variety of stochastic processes, in both discrete and continuous time. In both cases, we will use a subscript to indicate the time parameter: e.g., $X_{t}$ or $G_{k}$, where t takes values in the non-negative reals ${\mathbb{R}}_{+}$ and k in the non-negative integers ${\mathbb{Z}}_{+}$. In the case of continuous-time processes, we will assume that sample paths are càdlàg i.e., right-continuous with left limits. We will frequently need to refer to the left-limit of such a process. Accordingly, if $\Phi_{t}$ is a càdlàg random process, we definè

$${\tilde{\Phi }}_{t}:=\{\begin{array}{cc} \underset{s↑t}{\lim}\Phi_{s}, & t>0,\\ \Phi_{0}, & t=0. \end{array}$$

Note that ${\tilde{\Phi }}_{t}$ is thus left-continuous with right limits.

If $\Phi_{t}$, $t\in {\mathbb{R}}_{+}$ is a pure jump process, knowledge of its sample path is equivalent to knowledge of the number, $K_{t}$, of jumps it has taken as of time t, the jump times ${\hat{T}}_{k}$, and the embedded chain ${\hat{\Phi }}_{k}:=\Phi_{{\hat{T}}_{k}}$, $k=0,\ldots ,K_{t}$.

In particular, if we adopt the convention that ${\hat{T}}_{0}=0$ and ${\hat{T}}_{K_{t}+1}=t$, then $\Phi_{t}={\hat{\Phi }}_{k}$ for $t\in \left[{\hat{T}}_{k},{\hat{T}}_{k+1}\right)$, $k=0,\ldots ,K_{t}$.

### 2.2. Population process.

**Figure 1.**
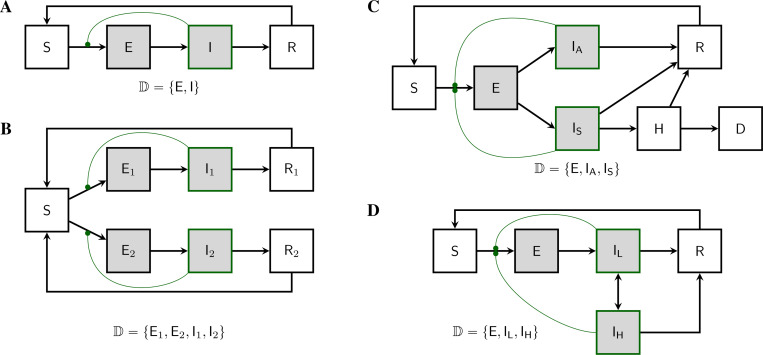
Examples of discretely-structured population models. Demes are shaded. Compartments containing infectious hosts are outlined in green. Curved green lines connect transmission rates with the compartments whose occupancies control their modulation; each such connection gives rise to a nonlinearity in the model. **(A)** An SEIRS model. Susceptible individuals (S), once infected, enter a transient incubation phase (E) before they become infectious (I). Upon recovery (R), individuals experience immunity from reinfection. If this immunity wanes, they re-enter the susceptible compartment. Pathogen lineages are to be found in hosts within the E and I compartments only. Accordingly, there are two demes: D={E,I}. If there is exactly one lineage per host, then the occupancy, $n\left(X_{t}\right)=\left(n_{\text{E}}\left(X_{t}\right),n_{\text{I}}\left(X_{t}\right)\right)$, is the integer 2-vector giving the numbers of hosts in the respective compartments. See §2.6 for definition and discussion of demes and deme occupancy. **(B)** In this four-deme model, two distinct pathogen strains compete for susceptibles. **(C)** A three-deme model according to which, after an incubation period, hosts may develop asymptomatic infection $\left({\text{I}}_{\text{A}}\right)$. If they do not recover, symptomatically infected hosts $\left({\text{I}}_{\text{S}}\right)$ can progress to hospitalization (H) and death (D). **(D)** A three-deme model with heterogeneity in transmission behavior. Contagious individuals move randomly between low-transmission $\left({\text{I}}_{\text{L}}\right)$ and high-transmission $\left({\text{I}}_{\text{H}}\right)$ behaviors.

We are motivated by the desire for exact phylodynamic inference methods for as wide a class of epidemiological models as possible. In particular, we would like to be able to formulate and parameterize an arbitrary compartmental model and to quantify its ability to explain data using likelihood. Fig. 1 depicts a few such models in order to give a sense of the kinds of complexities that can arise. Of course, with the ability to entertain models with countably many compartments, much greater complexity is possible. In particular, one can model not only complex infection progression, but also strain structure, behavioral structure, age structure, and spatial structure using compartmental models. As is well known, one can discretize continuous structure-variables and employ the linear chain trick to accommodate non-exponential residence times. While the utility of these approximations will vary, a very wide range of model assumptions lie within the scope of the theory presented here.

We will assume that our population process is a time-inhomogeneous Markov jump process, $X_{t}$, $t\in {\mathbb{R}}_{+}$, taking values in some space X. In earlier work (King et al., 2022), we limited ourselves to the case $X={\mathbb{Z}}^{d}$, but here we assume only X is a complete metric measure space with a countable dense subset. The population process is completely specified by its initial-state density, $p_{0}$, and its transition rates α. In particular, we suppose that

(1) $$\text{Prob}\left[X_{0}\in \text{𝓔}\right]=\int_{\text{𝓔}}p_{0}(x)\text{d}x$$

for all measurable sets 𝓔⊆X. For any $t\in {\mathbb{R}}_{+},x,x^{\prime }\in X$, we think of the quantity $\alpha \left(t,x,x^{\prime }\right)$ as the instantaneous hazard of a jump from x to $x^{\prime }$. More precisely, the transition rates have the following properties:

$$\alpha \left(t,x,x^{\prime }\right)⩾0,\;\int_{X}\alpha \left(t,x,x^{\prime }\right)\text{d}x^{\prime }<\infty ,$$

for all $t\in {\mathbb{R}}_{+}$ and $x,x^{\prime }\in X$ and that, as a function of time, α is continuous almost everywhere. Henceforth, we understand that integrals are taken over all of X unless otherwise specified. Let $K_{t}$ be the number of jumps that X has taken by time t. We assume that $K_{t}$ is a simple counting process so that

$$\text{Prob}\left[K_{t+\text{Δ}}=n+1|K_{t}=n\right]=\text{Δ}\int \alpha \left(t,x,x^{\prime }\right)\text{d}x^{\prime }+o(\text{Δ}),\;\text{Prob}\left[K_{t+\text{Δ}}>n+1|K_{t}=n\right]=o(\text{Δ}),\;\text{Prob}\left[X_{t+\text{Δ}}\in \text{𝓔}|X_{t}=x,K_{t+\text{Δ}}-K_{t}=1\right]=\frac{\int_{\text{𝓔}}\alpha \left(t,x,x^{\prime }\right)\text{d}x^{\prime }}{\int \alpha \left(t,x,x^{\prime }\right)\text{d}x^{\prime }}+o(\text{Δ}).$$

We further assume that $\alpha \left(t,x,x^{\prime }\right)$ is càdlàg as a function of time for all $x,x^{\prime }\in X$ and that the number of jumps that occur in a finite time-interval is finite, i.e., $\text{Prob}\left[K_{t}<\infty \right]=1$ for all t.

### 2.3. Kolmogorov forward equation.

The above may be compactly summarized by stating that if v(t,x) satisfies the Kolmogorov forward equation (KFE),

(2) $$\frac{\partial v}{\partial t}(t,x)=\int v\left(t,x^{\prime }\right)\alpha \left(t,x^{\prime },x\right)\text{d}x^{\prime }-\int v(t,x)\alpha \left(t,x,x^{\prime }\right)\text{d}x^{\prime },$$

and if, moreover, $v(0,x)=p_{0}(x)$, then $\int_{\text{𝓔}}v(t,x)\text{d}x=\text{Prob}\left[X_{t}\in \text{𝓔}\right]$ for every measurable 𝓔⊆X. Eq. 2 is sometimes called the *master equation* for $X_{t}$.

### 2.4. Inclusion of jumps at deterministic times.

For modeling purposes, it is sometimes desirable to insist that certain events occur at known times. For example, if samples are collected at specific times in such a way that the timing itself conveys no information about the process, one might wish to condition on the sampling time. We can expand the class of population models to allow for this as follows. Suppose that $S=\left\{s_{1},s_{2},\ldots ,\right\}\subset {\mathbb{Z}}_{+}$ is a sequence of event times. Let us postulate that, at each of these times, an event occurs at which $X_{t}$ jumps according to a given probability kernel π. In particular, for any state x∈X and measurable $\text{𝓔}\subset X,\pi \left(s_{i},x,\text{𝓔}\right)$ is the probability that the jump at time $s_{i}$ is to 𝓔, conditional on the state just before the jump being x. With this notation, the KFE for the process becomes

(3) $$\frac{\partial v}{\partial t}(t,x)=\int v\left(t,x^{\prime }\right)\alpha \left(t,x^{\prime },x\right)\text{d}x^{\prime }-\int v(t,x)\alpha \left(t,x,x^{\prime }\right)\text{d}x^{\prime },\;t\notin S,$$

(4) $$v(t,x)\text{d}x=\int \tilde{v}\left(t,x^{\prime }\right)\pi \left(t,x^{\prime },\text{d}x\right)\text{d}x^{\prime },\;t\in S.$$

Note that the Eq. 3 is identical to Eq. 2; we call this the regular part of the KFE. We refer to Eq. 4 as the *singular part* of the KFE.

As a matter of notation, one can represent Eqs. 3 and 4 as a single equation in the form of Eq. 2. In particular, if in Eq. 2 we make the substitution

$$\alpha \left(t,x,x^{\prime }\right)\mapsto \alpha \left(t,x,x^{\prime }\right)+\sum_{s\in S}\delta (t,s)\frac{\text{d}\pi }{\text{d}x^{\prime }}\left(t,x,x^{\prime }\right),$$

we obtain an equation which we can view as shorthand for Eqs. 3 and 4. Here, δ(t,s) is a Dirac delta function and $\text{d}\pi /\text{d}x^{\prime }$ denotes the density (i.e., Radon-Nikodym derivative) of π with respect to the measure on X.

### 2.5. Jump marks.

It will be useful to divide the jumps of the population process $X_{t}$ into distinct categories, which differ with respect to the changes they induce in a genealogy. For this purpose, we let U be a countable set of jump *marks* such that

$$\alpha \left(t,x,x^{\prime }\right)=\sum_{u\in U}\alpha_{u}\left(t,x,x^{\prime }\right).$$

**Figure 2.**
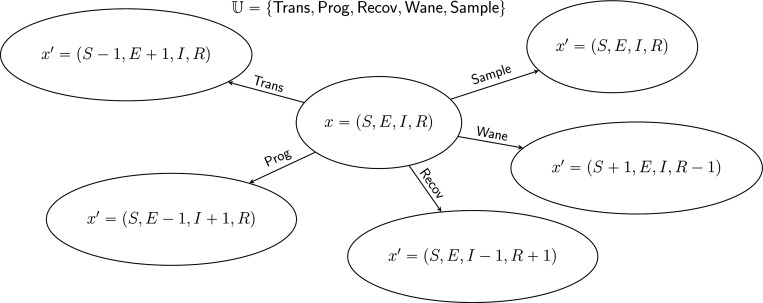
Markov state transition diagram for the SEIRS model depicted in Fig. 1A. The state, x, is characterized by four numbers, S, E, I, and R. From a given state x, there are five possible kinds of jumps $x\mapsto x^{\prime }$. Accordingly, the set, U, of jump marks has five elements. Each of these is of a different type: Trans (transmission) is of birth type, Prog (progression) is of migration type, Recov (recovery) is of death type, Sample (sampling) is of sample type, and Wane (loss or waning of immunity) is of neutral type. See §3.1 for a description of these jump types. Note that, in this formulation, when a sampling event occurs, the state does not change.

Fig. 2 shows an example for U has five elements. In the following, sums over u are to be taken over the whole of U unless otherwise indicated.

Let us define the *jump mark* process, $U_{t}$, to be the mark of the latest jump as of time t. As usual, we take the sample paths of $U_{t}$ to be càdlàg. Observe that, though $X_{t}$ and $\left(X_{t},U_{t}\right)$ are Markov processes, $U_{t}$ is not.

### 2.6. Demes and deme occupancy.

Our first goal in this paper is to show how a given population process induces a unique stochastic process on the space of genealogies. At each time, this genealogy will represent the relationships of shared ancestry among a population of lineages extant at that time. To accommodate the structure of the population, this population of lineages will itself be subdivided into discrete categories. In particular, we suppose that there are a countable set of subpopulations, within each of which individual lineages are exchangeable. We call these subpopulations *demes*, and use the symbol D to denote an index set for them. Fig. 1 illustrates this concept in the context of several compartmental models.

We define the *deme occupancy* function $n:D\times X\to {\mathbb{Z}}_{+}$ so that for i∈D, x∈X, $n_{i}(x)$ is the number of lineages in deme i when the population is in state x.

### 2.7. Examples.

The class of population models to which the theory presented here applies is very broad indeed. In particular, it encompasses the entire class of compartmental models with time-dependent flow rates. Here, to give a sense of this breadth, we briefly describe a few models of interest. Appendix B works out the theory for each of these examples.

#### SIRS model.

King et al. (2022) worked out formulas for the exact likelihood of a genealogy induced by an SIRS model. The theory developed in this paper applies, but since there is only one deme in this model, this is a simple case.

#### SEIRS model.

A simple, yet interesting, model with more than one deme is the SEIRS model (Fig. 1A). The state space is ${\mathbb{Z}}_{+}^{4}$, with the state x=(S,E,I,R) defined by the numbers of hosts in each of the four compartments. It has two demes: D={E,I}. The deme occupancy function in this case is n(x)=(E,I). Note that the terms associated with sampling cancel each other in the KFE, since, in this model, sampling has no effect on the state.

#### Two-strain competition model.

A simple model for the competition of two strains for susceptible hosts is depicted in Fig. 1B. In this model, the state vector consists of seven numbers: $x=\left(S,E_{1},E_{2},I_{1},I_{2},R_{1},R_{2}\right)$. There are four demes $\left(D=\left\{{\text{E}}_{1},{\text{E}}_{2},{\text{I}}_{1},{\text{I}}_{2}\right\}\right)$ and the occupancy function is $n(x)=\left(E_{1},E_{2},I_{1},I_{2}\right)$.

#### Superspreading model.

Fig. 1D depicts a model of superspreading. There are three demes $\left(D=\left\{\text{E},{\text{I}}_{\text{L}},{\text{I}}_{\text{H}}\right\}\right)$.

#### Linear birth-death model.

The linear birth-death process, a mainstay of existing phylodynamic methods, is a special case of the theory presented here. For this process, we have $X={\mathbb{Z}}_{+}$ and there is a single deme. $X_{t}$ represents the size of a population and $n\left(X_{t}\right)=X_{t}$.

#### Moran model and the Kingman coalescent.

The Kingman (1982a) coalescent is another workhorse in existing phylodynamic approaches. It is the ancestral process for the Moran model, in which a fixed population of n lineages experiences events at times distributed according to a rate-μ Poisson process. At each such event, an individual lineage selected uniformly at random dies and is replaced by the offspring of a second randomly selected lineage.

### 2.8. History.

Consider the Markov process $\left(X_{t},U_{t}\right)$. We define its *history process*, $H_{t}$, to be the restriction of the random function $s\mapsto \left(X_{s},U_{s}\right)$ to the interval [0,t]. Note that $H_{t}$ is itself trivially a Markov process, since it contains its own history.

Alternatively, one can think of $H_{t}$ as consisting of the sequence ${\left(\left({\hat{\text{T}}}_{k},{\hat{\text{X}}}_{k},{\hat{\text{U}}}_{k}\right)\right)}_{k=0}^{K_{t}}$. In particular, conditional on $H_{t}$, both $X_{t}$ and $U_{t}$ are deterministic, as are $K_{t}$, the embedded chains, ${\hat{\text{X}}}_{k}$, ${\hat{\text{U}}}_{k}$, and the point process of event times ${\hat{\text{T}}}_{k}$. The probability measure on the space of histories can be expressed in terms of these:

(5) $$\text{Prob}\left[{\text{dH}}_{t}\right]=p_{0}\left({\hat{\text{X}}}_{0}\right){\text{d}\hat{\text{X}}}_{0}\overset{K_{t}}{\underset{k=1}{\Pi }}\alpha_{{\hat{\text{U}}}_{k}}({\hat{\text{T}}}_{k},{\hat{\text{X}}}_{k-1},{\hat{\text{X}}}_{k}){\text{d}\hat{\text{X}}}_{k}{\text{d}\hat{\text{T}}}_{k}\exp\left(-\sum_{k=0}^{K_{t}}\int_{{\hat{\text{T}}}_{k}}^{{\hat{\text{T}}}_{k+1}}\sum_{u}\int \alpha_{u}\left(t^{\prime },{\hat{\text{X}}}_{k},x^{\prime }\right)\text{d}x^{\prime }\text{d}t^{\prime }\right)\text{,}$$

where again, by convention, ${\hat{\text{T}}}_{0}=0$ and ${\hat{\text{T}}}_{K_{t}+1}=t$.

If H is such a history, we define t(H) to be the right endpoint of its domain and use the notation $\text{ev}(\text{H}):=\left\{{\hat{\text{T}}}_{1},\ldots ,{\hat{\text{T}}}_{{\text{K}}_{t}}\right\}\subset [0,\text{t}(\text{H})]$ to denote the set of its jump times.

### 2.9. Genealogies.

**Figure 3.**
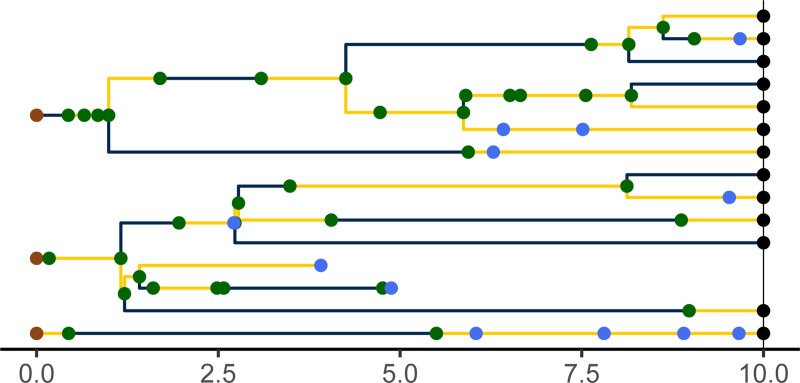
A genealogy, G, specifies the relationships of shared ancestry (via its tree-structure) and deme occupancy histories (via the coloring of its branches) of a set of lineages extant at some time t(G), as well as some samples gathered at earlier times. Here, t(G)=10 and there are two demes, D={blue, yellow}. Tip nodes, denoting extant lineages, are shown as black dots; sample nodes are shown as blue dots; internal nodes are indicated in green. Note that internal nodes occur not only at branch-points, but also inline (i.e., along branches). Wherever a lineage moves from one deme (color) to another, an internal node occurs; the converse does not necessarily hold.

A *genealogy*, G, encapsulates the relationships of shared ancestry among a set of lineages that are extant at some time $\text{t}(G)\in {\mathbb{R}}_{+}$ and perhaps a set of samples collected at earlier times (Fig. 3). A genealogy has a tree- or forest-like structure, with four distinct kinds of nodes: (i) *tip nodes*, which represent labeled extant lineages; (ii) *internal nodes*, which represent events at which lineages diverged and/or moved from one deme to another; (iii) *sample nodes*, which represent labeled samples; and (iv) *root nodes*, at the base of each tree. Each node a is associated with a specific time, t(a). In particular, if a is a tip node in G, then t(a)=t(G); if a is a sample node, then t(a)⩽t(G) is the time at which the sample was taken. Moreover, if node a is ancestral to node $a^{\prime }$, then $\text{t}(a)⩽\text{t}\left(a^{\prime }\right)$ and $\text{t}\left(a^{\prime }\right)-\text{t}(a)$ is the distance between a and $a^{\prime }$ along the genealogy. Without loss of generality we assume that t(a)=0 for all root nodes a. We let ev(G) denote the set of all internal and sample node-times of the genealogy G; we refer to these as *genealogical event times*.

Importantly, a genealogy informs us not only about the shared ancestry of any pair of lineages, but also about where in the set of demes any given lineage was at all times. Accordingly, we can visualize a genealogy as a tree, the nodes and edges of which are painted with a distinct color for each deme (Fig. 3). Note that a genealogy will in general have *branch-point nodes*, i.e., internal nodes with more than one descendant, but may also have internal nodes with only one descendant. We refer to such nodes as *inline nodes*. These occur whenever the color changes along a branch, but can also occur without a color-change.

Formally, we define a genealogy, G, to be a triple, (T,Z,Y), where $T=\text{t}(G)\in {\mathbb{R}}_{+}$ is the *genealogy time*, Z specifies the genealogy’s *tree structure*, and Y gives the *coloring*. In particular, let L be a countable set of labels and let partit(L) be the set of all collections of finite, mutually-disjoint subsets of L. That is, an element z∈partit(L) is a partition of the finite set $\cup_{z}\subseteq L$. Partition *fineness* defines a partial order on partit(L). Specifically, for $z,z^{\prime }\in \text{partit}\left(L\right)$, we say $z≼z^{\prime }$ if and only if for every $b^{\prime }\in z^{\prime }$ there is b∈z such that $b⊇b^{\prime }$. The tree structure of G is defined by a càdlàg map Z:[0,T]→partit(L) that is monotone in the sense that $t_{1}⩽t_{2}$ implies $Z_{t_{1}}≼Z_{t_{2}}$. An element $b\in Z_{t}$ is a set of labels; it represents the branch of the tree that bears the corresponding lineages. We use the notation ev(Z) to denote the set of times at which Z is discontinuous. Note that ev(Z) includes the times of all tip, sample, and branch-point nodes, but excludes inline and root nodes. Therefore, ev(Z)⊆ev(G).

The third element of G specifies the coloring of branches and locations of tip, sample, and internal nodes (including inline nodes). Mathematically, if G=(T,Z,Y), then Y is a càdlàg function that maps each point on thè genealogy to a deme and a non-negative integer. In particular, if t∈[0,T] and a is the label of any tip or sample node, $Y_{t}(a)=\left(Y_{t}^{\text{d}}(a),Y_{t}^{\text{m}}(a)\right)\in D\times {\mathbb{Z}}_{+}$, where $Y_{t}^{\text{d}}(a)$ is the deme in which the lineage of a is located at time t and $Y_{t}^{\text{m}}(a)$ is the number of internal or sample nodes encountered along the lineage of a in going from time 0 to time t. In particular, $Y_{t}^{\text{m}}(a)$ is a simple counting process, with $Y_{0}^{\text{m}}(a)=0$ for all a. Since a, $a^{\prime }\in b\in Z_{t}$ implies $Y_{t}(a)=Y_{t}\left(a^{\prime }\right)$, one can equally well think of $Y_{t}$ as a map $Z_{t}\to D\times {\mathbb{Z}}_{+}$. Given a tree Z, we let Y(Z) denote the set of colorings Y that are compatible with Z. We moreover define ${\text{Y}}_{t}(Z):=\left\{Y_{t}|Y\in \text{Y}(Z)\right\}$. Formally speaking, Y(Z) is a fiber bundle over Z, each ${\text{Y}}_{t}(Z)$ being a fiber.

It will sometimes be convenient to make use of notation whereby a genealogy $G=\left(\text{t}(G),G^{\text{Z}},G^{\text{Y}}\right)$.

### 2.10. Binomial ratio.

For $n,r,\ell ,s\in {\mathbb{Z}}_{+}^{D}$, define the *binomial ratio*

$$\left(\begin{array}{cc} n & \text{𝓁}\\ r & s \end{array}\right):=\{\begin{array}{cc} \frac{\prod_{i\in D}\left(\begin{array}{c} n_{i}-{\text{𝓁}}_{i}\\ r_{i}-s_{i} \end{array}\right)}{\prod_{i\in D}\left(\begin{array}{c} n_{i}\\ r_{i} \end{array}\right)}, & \text{if ∀}{\mathrm{i n}}_{i}⩾\left\{{\text{𝓁}}_{i},r_{i}\right\}⩾s_{i}⩾0,\\ 0, & \text{otherwise}. \end{array}$$

Observe that $\left(\begin{array}{cc} n & \text{𝓁}\\ r & s \end{array}\right)\in [0,1]$. Moreover, in consequence of the Chu-Vandermonde identity, we have

$$\sum_{s\in {\mathbb{Z}}_{+}^{D}}\left(\begin{array}{cc} n & \text{𝓁}\\ r & s \end{array}\right)\left(\begin{array}{c} \text{𝓁}\\ s \end{array}\right)=1,$$

whenever $n_{i}⩾\left\{\ell_{i},r_{i}\right\}⩾0$ for all i.

### 3. The induced genealogy process.

#### 3.1. Event types.

We now show how a given population process naturally induces a process in the space of genealogies. Specifically, at each jump in the population process, a corresponding change occurs in the genealogy, according to whether lineages branch, die, move between demes, or are sampled. For this purpose, there are five distinct *pure types* of events:

(a) *Birth-type events* result in the branching of one or more new lineages, each from some existing lineage. Examples of birth-type events include transmission events, speciations, and actual births. Importantly, we assume that all new lineages arising from a birth event share the same parent and that at most one birth event occurs at a time, almost surely.
(b) *Death-type events* result in the extinction of one or more lineages. Examples include recovery from infection, death of a host, and species extinctions. We allow for the possibility that multiple lineages die simultaneously.
(c) *Migration-type events* result in the movement of a lineage from one deme to another. Spatial movements, changes in host age or behavior, and progression of an infection can all be represented as migration-type events. We permit multiple lineages to move simultaneously.
(d) *Sample-type events* result in the collection of a sample from a lineage. We allow for the possibility that multiple samples are collected simultaneously, though we require that, in this case, each extant lineage is sampled at most once.
(e) *Neutral-type events* result in no change to any of the lineages.

Fig. 2 depicts an example with jumps of all five pure types. It is not necessary that an event be of a pure type; *compound events* partake of more than one type. For example, a sample/death-type event, in which a lineage is simultaneously sampled and removed, has been employed (Leventhal et al., 2014), as have birth/death events in which one lineage reproduces at the same moment that another dies (e.g., the Moran (1958) process). The theory presented here places few restrictions on the complexity of the events that can occur by combining events of the various pure types.

### 3.2. Genealogy process.

We now show how a given population process induces a stochastic process, $G_{t}$, on the space of genealogies. In the case of unstructured population processes (i.e., those having a single deme), King et al. (2022) gave a related construction that is equivalent to the one presented here.

**Figure 4.**
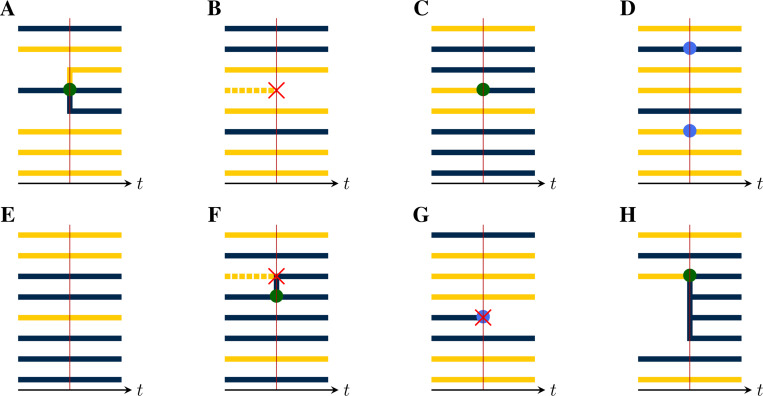
Event types differ by their effects on the genealogy. This can be seen by examining the local structure of the genealogy in the neighborhood of a jump. (**A**) A birth-type jump results in the branching of one or more child lineages from the parent. There can be only one parent, though the demes of the child lineages may differ from that of their parent. Here, a parent of the blue deme sires one child lineage in each of the blue and yellow demes. The *production* of an event is an integer vector, with one entry for each deme. The production of this event is therefore $r=\left(r_{\text{blue}},r_{\text{yellow}}\right)=(2,1)$. The *deme occupancy* of an event is the number of lineages in each deme just to the right of the event. The deme occupancy at this event is therefore $n=\left(n_{\text{blue}},n_{\text{yellow}}\right)=(3,5)$. (**B**) A death-type event causes the extinction of a lineage. Since internal nodes without children are recursively removed, the affected branch is dropped. The production of this event is r=(0,0) and the deme occupancy is n=(3,4). (**C)** A migration-type event results in the movement of one or more lineages from one deme to another. Here, one lineage moves from the yellow to the blue deme. The production of this event is r=(1,0), i.e., the production is 1 for the blue deme and 0 for the yellow. The deme occupancy is n=(6,2). (**D**) In a sample-type event, one or more sample nodes (blue circles) are inserted. Here, there are two samples, one in each of the blue and yellow demes. Accordingly, r=(1,1) and n=(2,6). (**E**) A neutral-type event has no effect on the genealogy and zero production in all demes: r=(0,0)*, n=(5,3)*. (**F**) The theory presented here allows for compound events. As an example, here a birth/death-type event occurs, wherein one yellow lineage is extinguished and a blue lineage simultaneously sires a blue child. For this event, we have r=(2,0), and n=(6,2). (**G**) Here, a compound sample/death-type event with r=(0,0) and n=(2,5) occurs. A blue lineage is sampled and simultaneously extinguished. Note that recursive removal does not occur, since sample nodes are never removed. (**H**) A compound birth/migration-type event with r=(4,0) and n=(6,2).

At each jump in the population process, a change is made to the genealogy, according to the mark, u, of the jump (Fig. 4). In particular:

(a) If u is of birth-type (Fig. 4A), it results in the creation of one new internal node, call it b. A tip node, a, of the appropriate deme is chosen with uniform probability from among those present and b is inserted so that its ancestor is that of a, while a takes b as its ancestor. One new tip node, of the appropriate deme, is created for each of the children, all of which take b as their immediate ancestor.
(b) If u is of death-type (Fig. 4B), one or more tip nodes of the appropriate demes are selected with uniform probability from among those present. These are deleted. Next, internal nodes without children are recursively removed. Sample nodes are never removed.
(c) At a migration-type event (Fig. 4C), the appropriate number of migrating lineages are selected at random with uniform probability, from among those present in the appropriate demes. For each selected lineage, one new branch node is inserted between the selected tip node and its ancestor. The color of the descendant branch changes accordingly.
(d) At a sample-type event (Fig. 4D), the appropriate number of sampled lineages are selected at random from among the tip nodes, with uniform probability according to deme. One new sample node is introduced for each selected lineage: each is inserted between a selected tip nodes and its ancestor.
(e) At a neutral-type event (Fig. 4E), no change is made to the genealogy.
(f) Finally, events of compound type (e.g., Fig. 4F–H) are accommodated by combining the foregoing rules.

In each of these events, the new node or nodes that are introduced have node-times equal to the time of the jump.

#### 3.2.1. Emergent lineages and production.

The lineages which descend from an inserted node are said to *emerge* from the event. Thus, after a birth-type event, the emerging lineages include all the new offspring as well as the parent. Likewise, at pure migration- or sample-type events, each migrating or sampled lineage emerges from the event. At pure death-type events, no lineages emerge. In general, at an event of mark u, there are $r_{i}^{u}$ emergent lineages in deme i. We require that $r_{i}^{u}$ be a constant, for each u and i. Thus there is a function $r:U\times D\to {\mathbb{Z}}_{+}$, such that $r_{i}^{u}$ lineages of deme i emerge from each event of mark u. Since, in applications, one is free to expand the set of jump-marks U as needed, this is not a restriction on the models that the theory can accommodate. We say $r^{u}:={\left(r_{i}^{u}\right)}_{i\in D}$ is the *production* of an event of mark u. Note that the lineages that die as a result of an event do not count in the production but that a parent lineage that survives the event does count.

#### 3.2.2. Conditional independence and exchangeability.

Application of these rules at each jump of $X_{t}$ constructs a chain of genealogies ${\hat{\text{G}}}_{k}$. In particular, at each jump-time ${\hat{\text{T}}}_{k}$, the genealogy ${\hat{\text{G}}}_{k-1}$ is modified according to the jump-mark ${\hat{\text{U}}}_{k}$ to yield ${\hat{\text{G}}}_{k}$. We view ${\hat{\text{G}}}_{k}$ as the embedded chain of the continuous-time genealogy process $G_{t}$. It is very important to note that, conditional on $\left({\hat{\text{X}}}_{k},{\hat{\text{U}}}_{k}\right)$, the number of parents and number of offspring in each deme is determined and the random choice of which lineages die, migrate, are sampled, or sire offspring is independent of these choices at any other times and independent of $\left({\hat{\text{X}}}_{j},{\hat{\text{U}}}_{j}\right)$ for all j≠k. Moreover, by assumption, the lineages within each deme are exchangeable: any lineage within a deme is as likely as any other lineage in that deme to be selected as a parent or for death, sampling, or migration. Finally, note that $G_{t}$ does not have the Markov property, though $\left(X_{t},U_{t},G_{t}\right)$ and $\left(X_{t},G_{t}\right)$ do. Observe in passing that, if instead of dropping tip nodes at death events we were to retain them as we do samples, the resulting genealogy—which we might call the “complete” genealogy—would have the Markov property.

### 3.3. Pruned and obscured genealogies.

**Figure 5.**
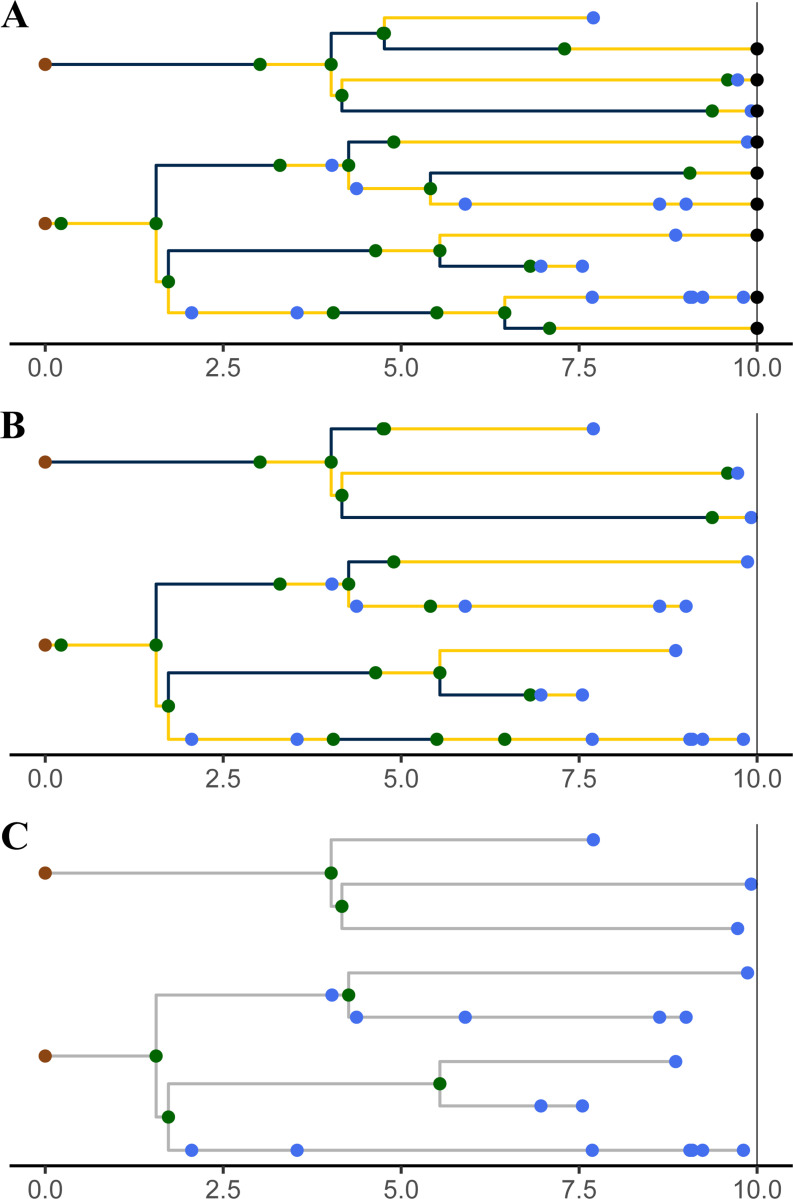
Unpruned, pruned, and obscured genealogies from a single realization of the genealogy process induced by the SEIRS model depicted in Figs. 1 and 2. (**A**) A realization of the unpruned genealogy process ${\text{G}}_{t}$ is shown at t=10. Tip nodes, corresponding to lineages alive at time t=10 are indicated with black points. Blue points represent samples; green points, internal nodes. Branches are colored according to the deme in which the corresponding lineage resided at that point in time: blue denotes E and yellow, I. (**B**) The genealogy is *pruned* by deleting all tip nodes and then recursively pruning away childless internal nodes. Sample nodes are never removed. (**C**) A pruned genealogy is *obscured* by effacing all deme information from lineage histories: the colors are erased, as are all inline nodes. See the text (§§2.9, 3.3.1, and 3.3.4) for more detail.

The process just described yields a genealogy that relates all extant members of the population, and all samples. Moreover, it details each lineage’s complete history of movement through the various demes. However, the data we ultimately wish to analyze will be based only on samples. Nor, in general, will the histories of deme occupancy be observable. A generative model must account for this loss of information. We therefore now describe how genealogies are *pruned* to yield sample-only genealogies and then *obscured* via the erasure of color from their branches (Fig. 5).

#### 3.3.1. Pruned genealogy.

Given a genealogy G, one obtains the *pruned genealogy*, P=prune(G) by first dropping every tip node and then recursively dropping every childless internal node (Fig. 5A–B). In a pruned genealogy only internal and sample nodes remain, and sample nodes are found at all of the leaves and possibly some of the interior nodes of the genealogy. Observe that a pruned genealogy is a colored genealogy: it retains information about where among the demes each of its lineages was through time (Fig. 5B). Note also that a pruned genealogy P is characterized by its time, t(P) and the functions $P^{\text{Y}}$ and $P^{\text{Z}}$ just as an unpruned genealogy is. Finally, observe that, since it contains within itself all of its past history, the pruned genealogy process $P_{t}=\text{prune}\left(G_{t}\right)$ is Markov, even though the unpruned genealogy process, $G_{t}$, is not.

#### 3.3.2. Lineage count and saturation.

**Figure 6.**
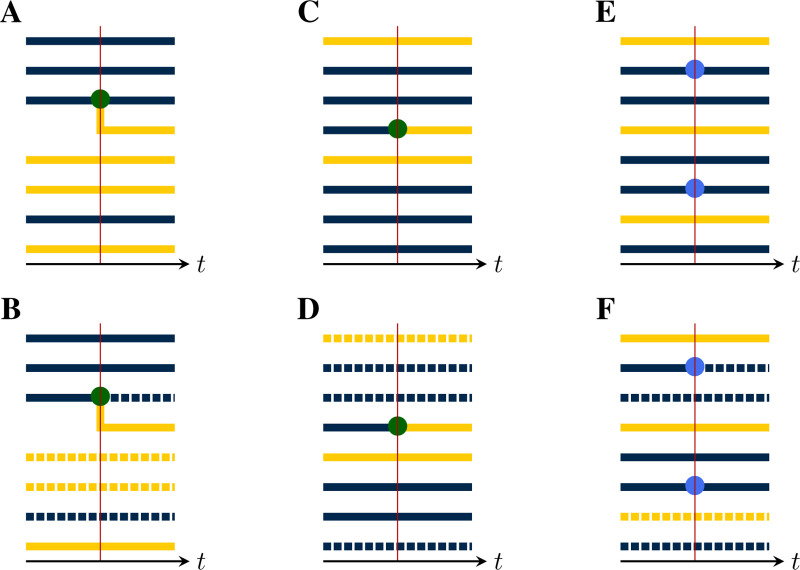
Lineage count and saturation. Each panel shows the neighborhood of a single event in the unpruned genealogy (top row) and the corresponding pruned genealogy (bottom row). Pruning consists of the removal of all branches that are not ancestral to some sample. In the bottom row of panels, pruned branches are indicated using broken lines. (**A**) A birth-type event with production $r=\left(r_{\text{blue}},r_{\text{yellow}}\right)=(1,1)$ occurs. (**B**) Suppose that pruning results in the removal of the dashed lineages. Then the lineage count at this event-time is $\ell =\left(\ell_{\text{blue}},\ell_{\text{yellow}}\right)=(2,2)$. The saturation is s=(0,1) since only a single, yellow lineage emerges from the event. (**C**) A migration-type event with production r=(0,1) occurs. (**D**) After pruning, ℓ=(2,2) and s=(0,1). **(E)** A sample-type event occurs in which two blue lineages are sampled (production r=(2,0)). **(F)** After pruning, ℓ=(2,2) and s=(1,0). Observe that in panels B and D, the local structures of the pruned genealogies are identical, though they arise from events of different type.

In the following, we will find that we need to count the deme-specific numbers of lineages present in a given pruned genealogy at a given time. Accordingly, suppose P=(T,Z,Y) is a pruned genealogy and suppose t∈[0,T]. Let $\ell_{i}$ denote the number of lineages in deme i at time t and $\ell :={\left(\ell_{i}\right)}_{i\in D}\in {\mathbb{Z}}_{+}^{D}$. Clearly, ℓ depends only $Y_{t}$. Therefore, we can define ℓ as a function such that, whenever P=(T,Z,Y) is a pruned genealogy, $\text{𝓁}\left(Y_{t}\right)$ is the vector of deme-specific lineage counts at time t. We refer to ℓ as the *lineage-count* function (cf. Fig. 6).

We will also have occasion to refer to the deme-specific number of lineages emerging from a given event. In particular, given a node time t in a pruned genealogy P=(T,Z,Y), the number $s_{i}$ of lineages of deme i emerging from all nodes with time t is well defined and we can write $s:={\left(s_{i}\right)}_{i\in D}$. Like the lineage-count we can define the *saturation* function such that, whenever P=(T,Z,Y) is a pruned genealogy, $s\left({\tilde{Y}}_{t},Y_{t}\right)$ is the function, s depends only on the local structure of P. However, s depends not only on $Y_{t}$, but also ${\tilde{Y}}_{t}$. Thus, we can define the *saturation* function such that, whenever P=(T,Z,Y) is a pruned genealogy, $s\left({\tilde{Y}}_{t},Y_{t}\right)$ is the integer vector of deme-specific numbers of emerging lineages at time t. Fig. 6 illustrates.

#### 3.3.3. Compatibility.

Suppose P is a pruned genealogy, with t(P)=T and t∈ev(P). The local structure of P at t is, in general, compatible with only a subset of the possible jumps U. For example, if the event in P at t is a branch node or a sample node, then it is compatible only with birth-type or sample-type jumps, respectively. Similarly, if the node in P at time t is one at which a lineage moves from deme i to deme $i^{\prime }$, then u must be either of $i\to i^{\prime }$ migration type or of a birth type with parent in i and $r_{i^{\prime }}^{u}>0$. To succinctly accommodate all possibilities, let us introduce the indicator function Q such that Q=1 if the local genealogy structure—which is captured by the values of $P^{Y}$ just before and after t—is compatible with an event of type u and Q=0 otherwise. That is, $Q_{u}\left(y,y^{\prime }\right)=1$ if and only if there is a feasible genealogy, G=(T,Z,Y), and history, H, and a t∈[0,T] such that, given $G_{\text{T}}=\text{G}$ and $H_{\text{T}}=\text{H}$, we have ${\text{U}}_{t}=u$, ${\tilde{\text{Y}}}_{t}=y$, and ${\text{Y}}_{t}=y^{\prime }$. We refer to Q as the *compatibility indicator*.

#### 3.3.4. Obscured genealogy.

The *obscured genealogy* is obtained by discarding all information about demes and events not visible from the topology of the tree alone (Fig. 5B–C). In particular, if P=(T,Z,Y) is a pruned genealogy, we write obs(P)=(T,Z) to denote the obscured genealogy.

## 4. Results.

### 4.1. Likelihood for pruned genealogies.

Our first result will be an expression for the likelihood of a given pruned genealogy given the history of the population process.

#### Theorem 1.

*Suppose P=(T,Z,Y) is a given pruned genealogy. Define*

(6) $$\phi_{u}\left(x,y,y^{\prime }\right):=\left(\begin{array}{cc} n(x) & \ell \left(y^{\prime }\right)\\ r^{u} & s\left(y,y^{\prime }\right) \end{array}\right)Q_{u}\left(y,y^{\prime }\right),$$

*where n is the deme occupancy (*§2.6*), $r^{u}$ is the production (*§3.2.1*), ℓ and s are the lineage-count and saturation functions, respectively (*§3.3.2*), Q is the compatibility indicator (*§3.3.3*), and the binomial ratio is as defined in* §2.10. *Then*

$$\text{Prob}\left[P_{\text{T}}=\text{P}|H_{\text{T}}=\text{H}\right]=𝟙\{\text{ev}(\text{H})⊇\text{ev}(\text{P})\}\prod_{t\in \text{ev}(\text{H})}\phi_{{\text{U}}_{t}}\left({\text{X}}_{t},{\tilde{\text{Y}}}_{t},{\text{Y}}_{t}\right).$$

*Proof.* If ev(H)⊉ev(P), then H and P are incompatible and $\text{Prob}\left[P_{\text{T}}=\text{P}|H_{\text{T}}=\text{H}\right]=0$. Similarly, if any event of H is incompatible with the local structure of P in the sense of §3.3.3, then $\text{Prob}\left[P_{\text{T}}=\text{P}|H_{\text{T}}=\text{H}\right]=0$. Let us therefore suppose that neither of these conditions hold. Conditional on $H_{\text{T}}=\text{H}$, at each time t∈ev(H), a jump of mark ${\text{U}}_{t}$ occurred, with a production of $r^{{\text{U}}_{t}}={\left(r_{i}\right)}_{i\in D}$, resulting in a deme-occupancy of $n\left({\text{X}}_{t}\right)={\left(n_{i}\right)}_{i\in D}$. In P, at time t, there are $\ell_{i}=\ell_{i}\left({\text{Y}}_{t}\right)$ lineages in deme i, of which $s_{i}=s_{i}\left({\tilde{\text{Y}}}_{t},{\text{Y}}_{t}\right)$ are emergent. By assumption, at each genealogical event, lineages within a deme are exchangeable: each has an identical probability of being involved. This exchangeability implies that each lineage present in a deme at time t was equally likely to have been one of the emergent lineages. In particular, at time t, the probability that $s_{i}$ of the $\ell_{i}$ deme-*i* lineages were among the $r_{i}$ of $n_{i}$ lineages emergent in the unpruned genealogy process is the same as the probability that, upon drawing $\ell_{i}$ balls without replacement from an urn containing $r_{i}$ red balls and $n_{i}-r_{i}$ black balls, exactly $s_{i}$ of the drawn balls are red, namely

$$\frac{\left(\begin{array}{c} n_{i}-\ell_{i}\\ r_{i}-s_{i} \end{array}\right)\left(\begin{array}{c} \ell_{i}\\ s_{i} \end{array}\right)}{\left(\begin{array}{c} n_{i}\\ r_{i} \end{array}\right)}.$$

Because our lineages are labeled, each of the $\left(\begin{array}{c} \ell_{i}\\ s_{i} \end{array}\right)$ equally probable sets of $s_{i}$ lineages is distinct; just one of these is the one present in P. Moreover, since, again conditional on $H_{\text{T}}=\text{H}$, the identities of the lineages involved in a genealogical event are random and independent of the identities selected at all other events, we have established that

$$\text{Prob}\left[P_{\text{T}}=\text{P}|H_{\text{T}}=\text{H}\right]=\prod_{t\in \text{ev}(\text{H})}\left(\begin{array}{cc} n\left({\text{X}}_{t}\right) & \ell \left({\text{Y}}_{t}\right)\\ r^{{\text{U}}_{t}} & s\left({\tilde{\text{Y}}}_{t},{\text{Y}}_{t}\right) \end{array}\right).$$

Returning to the possibility that H is incompatible with P, since $\text{Prob}\left[P_{\text{T}}=\text{P}\right]=0$ if either any $Q_{{\text{U}}_{t}}=0$ or ev(P)⊈ev(H), we obtain the result.

Next, we show how the likelihood of a pruned genealogies, unconditional on the history, can be computed. For this, we use the filter equation technology developed in Appendix A. In particular, the following theorem follows immediately from Lemma A2.

#### Theorem 2.

*Suppose that P=(T,Z,Y) is a given pruned genealogy. Suppose that w=w(t,x) satisfies the initial condition $w(0,x)=p_{0}(x)$ and the filter equation*

(7) $$\frac{\partial w}{\partial t}(t,x)=\sum_{u}\int w\left(t,x^{\prime }\right)\alpha_{u}\left(t,x^{\prime },x\right)\phi_{u}\left(x,{\tilde{\text{Y}}}_{t},{\text{Y}}_{t}\right)\text{d}x^{\prime }-\underset{u}{\sum }\int w(t,x)\alpha_{u}\left(t,x,x^{\prime }\right)\text{d}x^{\prime },\;t\notin \text{ev}(\text{P}),\;w(t,x)=\underset{u}{\sum }\int \tilde{w}\left(t,x^{\prime }\right)\alpha_{u}\left(t,x^{\prime },x\right)\phi_{u}\left(x,{\tilde{\text{Y}}}_{t},{\text{Y}}_{t}\right)\text{d}x^{\prime },\;t\in \text{ev}(\text{P}),$$

*where ϕ is defined in* Eq. 6*. Then the likelihood of P is*

𝓛(P)=∫w(T,x)dx.

### 4.2. Likelihood for obscured genealogies.

Our next result concerns the likelihood of a given obscured genealogy conditional on the history.

#### Theorem 3.

*Suppose that (T,Z) is a given obscured genealogy. Let q and π be probability kernels, such that for all x∈X and $y\in {\text{Y}}_{0}(\text{Z})$,*

$$q(x,y)⩾0,\;\sum_{y\in {\text{Y}}_{0}(\text{Z})}q(x,y)=1,$$

*and, for all $u\in U,t\in {\mathbb{R}}_{+},x,x^{\prime }\in X,y,y^{\prime }\in {\text{Y}}_{t}(\text{Z})$,*

$$\pi_{u}\left(t,x,x^{\prime },y,y^{\prime }\right)⩾0,\;\sum_{y^{\prime }\in {\text{Y}}_{t}(\text{Z})}\pi_{u}\left(t,x,x^{\prime },y,y^{\prime }\right)=1.$$

*Suppose moreover that $\pi_{u}\left(t,x,x^{\prime },y,y^{\prime }\right)>0$ whenever $\alpha_{u}\left(t,x,x^{\prime }\right)Q_{u}\left(y,y^{\prime }\right)>0$ and that q(x,y)>0 whenever $\text{Prob}\left[P_{0}^{Y}=y|X_{0}=x\right]>0$. Then there is a stochastic jump process $y_{t}$ with sample paths in Y(Z) such that $\left(X_{t},U_{t},y_{t}\right)$ is Markov and*

$$\text{Prob}\left[P_{\text{T}}^{\text{Z}}=\text{Z}|H_{\text{T}}=\text{H}\right]=𝟙\{\text{ev}(\text{H})⊇\text{ev}(\text{Z})\}E\left[\frac{1}{q\left(X_{0},y_{0}\right)}\prod_{t\in \text{ev}(\text{H})}\frac{\phi_{{\text{U}}_{t}}\left({\text{X}}_{t},{\tilde{y}}_{t},y_{t}\right)}{\pi_{{\text{U}}_{t}}\left(t,{\tilde{\text{X}}}_{t},{\text{X}}_{t},{\tilde{y}}_{t},y_{t}\right)}\right],$$

*where ϕ is defined in* Eq. 6 *and the expectation is taken over the sample paths of $y_{t}$.*

*Proof.* First, observe that, since obs is a deterministic operator,

(8) $$\text{Prob}\left[P_{\text{T}}^{\text{Z}}=\text{Z}|H_{\text{T}}=\text{H}\right]=E\left[𝟙\left\{P_{\text{T}}^{\text{Z}}=\text{Z}\right\}|H_{\text{T}}=\text{H}\right].$$

Our strategy will be to evaluate Eq. 8 using importance sampling: we will propose pruned genealogies compatible with Z as sample paths from a stochastic process driven by $X_{t}$ and evaluate the the expectation in Eq. 8 by summing over these paths. Conditional on $H_{\text{T}}=\text{H}$, the initial distribution q and probability kernel π generate a Markov chain, ${\hat{\text{y}}}_{k}$ such that

$$\begin{array}{l} \text{Prob}\left[{\hat{\text{y}}}_{0}|H_{\text{T}}=\text{H}\right]=q\left({\text{X}}_{0},{\hat{\text{y}}}_{0}\right),\;\text{Prob}\left[{\hat{\text{y}}}_{k}|{\hat{\text{y}}}_{k-1},H_{\text{T}}=\text{H}\right]=\pi_{{\hat{\text{U}}}_{k}}\left({\hat{\text{T}}}_{k},{\hat{\text{X}}}_{k-1},{\hat{\text{X}}}_{k},{\hat{\text{y}}}_{k-1},{\hat{\text{y}}}_{k}\right). \end{array}$$

The required process $y_{t}$ is the unique càdlàg process with event times ${\hat{\text{T}}}_{k}$ and ${\hat{\text{y}}}_{k}$ as its embedded chain. This construction of $y_{t}$ obviously guarantees that ev(H)⊇ev(y)⊇ev(Z) and that $\left(X_{t},U_{t},y_{t}\right)$ is Markov.

Now, for y∈Y(Z), let us define C(y)=(T,Z,y). Then, by construction, obs(C(y))=(T,Z) and, conversely, for every pruned genealogy P satisfying t(P)=T and ${\text{P}}^{\text{Z}}=\text{Z},C\left({\text{P}}^{\text{Y}}\right)=\text{P}$. Moreover, the conditions on the kernels q and π guarantee that, if $\text{Prob}\left[P_{\text{T}}=\text{P}|H_{\text{T}}=\text{H}\right]>0$ and ${\text{P}}^{\text{Z}}=\text{Z}$, then $\text{Prob}\left[y={\text{P}}^{\text{Y}}|H_{\text{T}}=\text{H}\right]>0$. We therefore have that

$$\text{Prob}\left[P_{\text{T}}^{\text{Z}}=\text{Z}|H_{\text{T}}=\text{H}\right]=E\left[\frac{\text{Prob}\left[P_{\text{T}}=C(y)|H_{\text{T}}=\text{H}\right]}{\pi (y|\text{H})}\right],$$

the expectation being taken with respect to the random process **y**. Here, by definition,

$$\pi (y|\text{H})=q\left({\text{X}}_{0},y_{0}\right)\prod_{t\in \text{ev}(\text{H})}\pi_{{\text{U}}_{t}}\left(t,{\tilde{\text{X}}}_{t},{\text{X}}_{t},{\tilde{y}}_{t},y_{t}\right).$$

The result then follows from Theorem 1.

Note that, since ${\text{Y}}_{t}(\text{Z})$ is finite, it is permissible, for example, to choose q and π to be uniform.

The final result shows how to compute the likelihood of an obscured genealogy. It is an immediate consequence of Theorem 3 and Lemma A2.

#### Theorem 4.

*Let V=(T,Z) be a given obscured genealogy. Then there are probability kernels q and π as in* Theorem 3 *such that if*

$$\beta_{u}\left(t,x,x^{\prime },y,y^{\prime }\right)=\alpha_{u}\left(t,x,x^{\prime }\right)\pi_{u}\left(t,x,x^{\prime },y,y^{\prime }\right),\;\Psi_{u}\left(t,x,x^{\prime },y,y^{\prime }\right)=\frac{\phi_{u}\left(x^{\prime },y,y^{\prime }\right)}{\pi_{u}\left(t,x,x^{\prime },y,y^{\prime }\right)},$$

*and if w=w(t,x,y) satisfies the initial condition $w(0,x,y)=p_{0}(x)𝟙\{q(x,y)>0\}$ and the filter equation*

$$\frac{\partial w}{\partial t}=\sum_{uy^{\prime }}\int w\left(t,x^{\prime },y^{\prime }\right)\beta_{u}\left(t,x^{\prime },x,y^{\prime },y\right)\Psi_{u}\left(t,x^{\prime },x,y^{\prime },y\right)\text{d}x^{\prime }-\sum_{uy^{\prime }}\int w(t,x,y)\beta_{u}\left(t,x,x^{\prime },y,y^{\prime }\right)\text{d}x^{\prime },\;t\notin \text{ev}(\text{Z}),\;w(t,x,y)=\sum_{uy^{\prime }}\int \tilde{w}\left(t,x^{\prime },y^{\prime }\right)\beta_{u}\left(t,x^{\prime },x,y^{\prime },y\right)\Psi_{u}\left(t,x^{\prime },x,y^{\prime },y\right)\text{d}x^{\prime },\;t\in \text{ev}(\text{Z}),$$

*then the likelihood of V is*

$$\text{𝓛}(V)=\sum_{y}\int w(T,x,y)\text{d}x.$$

Lemma A3 shows how this can be computed via Sequential Monte Carlo.

## 5. Discussion.

The theory presented here represents a strict generalization of the existing coalescent and birth-death process approaches to phylodynamic inference. In Appendix B, we demonstrate that both of the latter processes are special cases of the genealogical processes constructed here. Importantly, because the theory allows computation of the likelihood via strictly forward-in-time computations, it permits consideration of models for which time-reversal arguments are not available. Moreover, inasmuch as the formulae of Theorem 4 can be efficiently computed via sequential Monte Carlo, explicit expressions for transition probabilities are not needed: it is sufficient to be able to simulate from the population process. This feature of the algorithms—known as the *plug-and-play property* (He et al., 2010)—further expands the class of population models that can be confronted with data.

In particular, the theory gives us the freedom to choose models with many demes. For deterministic population models, Volz (2012) and Rasmussen et al. (2014) showed how one could accommodate discrete population structure. Their procedures involve solving a large number of differential equations backward in time, relying on the time-reversibility of deterministic dynamics. In general, this time-reversibility is not a property of stochastic processes.

Some existing methods put rather severe limits on the form of the sampling model and, as Volz & Frost (2014) pointed out, misspecification of the sampling model can lead to large inferential biases. With the theory presented here, essentially arbitrary specification of the sampling model is possible. In particular, one can posit sampling at a rate which is an arbitrary function of time and state and include discrete sampling events as well. It is also possible to condition on the existence of samples.

If Sequential Monte Carlo algorithms are used to compute the likelihoods of Theorem 4, then it is straightforward to simultaneously assimilate information from both time-series and genealogical data. One can therefore supplement traditional incidence, disease, or mortality time series with genealogical data in an inferential exercise.

A limitation of the theory is that the population models are assumed to be pure jump processes, which allows consideration of demographic stochasticity and environmental stochasticity modeled by jumps involving multiple individuals (Bretó & Ionides, 2011), but disallows stochastic processes with a diffusive component. It should be possible to incorporate of the full range of Markovian environmental stochasticity via extension of this theory to population models containing both diffusion and jump components.

The price of the theory’s flexibility is primarily computational. When Sequential Monte Carlo is used to evaluate the likelihood in Theorem 4, the computational effort scales linearly with the number of samples. In its most straightforward implementation—using an event-driven algorithm (e.g., Gillespie, 1977)—it scales nonlinearly with population size in general. However, stochastic simulation schemes are available that scale independently of population size (Higham, 2008). On the other hand, the importance sampling underlying Theorem 4 will in general require effort that is exponential in the number of demes. For models with many demes, therefore, approaches for ameliorating or circumventing this curse of dimensionality may be necessary. Critically, the substantial freedom one has in the choice of the importance-sampling distribution π can be exploited for this purpose. In particular, since it is permissible to “borrow information” from the future by means of the importance sampling, there is hope for highly efficient algorithmic computation.

## Supplementary Material

Supplement 1
