# EXACT PHYLODYNAMIC LIKELIHOOD VIA STRUCTURED MARKOV GENEALOGY PROCESSES

**Published:** 2025-07-08

**Authors:** AARON A. KING, QIANYING LIN, EDWARD L. IONIDES

**Affiliations:** Department of Ecology & Evolutionary Biology, Center for the Study of Complex Systems, and Department of Mathematics, University of Michigan, Ann Arbor, MI 48109 USA *and* Santa Fe Institute, 1399 Hyde Park Road, Santa Fe, NM 87501 USA; Theoretical Biology and Biophysics, Los Alamos National Laboratory, Los Alamos, NM 87545 USA; Department of Statistics, University of Michigan, Ann Arbor, MI 48109 USA

## Abstract

We show that each member of a broad class of Markovian population models induces a unique stochastic process on the space of genealogies. We construct this genealogy process and derive exact expressions for the likelihood of an observed genealogy in terms of a filter equation, the structure of which is completely determined by the population model. We show that existing phylodynamic methods based on either the coalescent or the linear birth-death processes are special cases. We derive some properties of filter equations and describe a class of algorithms that can be used to numerically solve them. Our results open the door to statistically efficient likelihood-based phylodynamic inference for a much wider class of models than is currently possible.

## 1. Introduction

When the genome of an infectious agent accumulates mutations on timescales similar to those of transmission and infection progression, the resulting pattern of differences among genomes contains information on the history of the pathogen’s passage through individual hosts and the host population. As Grenfell et al. (2004) observed, one can extract this information to gain insight into the structure and dynamics of the host-pathogen system. In particular, one can formalize mathematical models of transmission, estimate their parameters, and compare their ability to explain data, following standard statistical paradigms. This collection of tasks is known as *phylodynamic* inference; Alizon (2024) provides a recent review.

A common approach to phylodynamic inference involves building a mathematical linkage between the tree-like *genealogy* or *phylogeny* that expresses the relationships of shared ancestry among sampled genomes and a model of the dynamics of the transmission system. Various linkages are possible, but, to attain maximal statistical efficiency (i.e., lose the least information), it is desirable to be able to compute the likelihood function for models of interest. The likelihood function is the probability density of a given set of data, conditional on a given model, viewed as a function of the parameters of that model. While various kinds of data may be available, we focus here on the case where the data are a set of genomic sequences. Notationally, if the data, S, is a set of genome sequences, Φ a genealogical tree relating these sequences, E a model of sequence evolution, and D a dynamic transmission model, then the full likelihood is

ℒ(D,E)=f(S∣D,E)=∫f(S∣Φ,E)f(Φ∣D)dΦ,

where the integral is taken over all possible genealogies and we somewhat loosely use the symbol f for the various distinct probability densities, the nature of each of which is clear from its arguments. In this expression, f(S∣Φ,E) is typically the Felsenstein (2004) phylogenetic likelihood. The function f(Φ∣D), which links the phylogeny to the dynamic model, is the *phylodynamic likelihood*. In the Bayesian context, the phylodynamic likelihood is sometimes referred to as a *tree prior* (Möller et al., 2018; Volz & Siveroni, 2018). Its computation has remained out of reach, except in several special cases. This paper presents theory that facilitates its computation for a very broad range of dynamic models.

With few exceptions, existing approaches to the phylodynamic likelihood have been based on one of two mathematical idealizations. The first is the Kingman (1982a) coalescent, by which the likelihood of a given genealogy is computed using a reverse-time argument. This computation provides the exact likelihood for a genealogy resulting from a particular, constant population-size, dynamic model (the Moran model, e.g., Moran, 1958; Kingman, 1982b; Möhle, 2000; King et al., 2020, 2022). Extensions of this approach develop approximate likelihoods for the case when the population size varies as a function of time (Griffiths & Tavaré, 1994; Drummond et al., 2005) or according to an SIR process (Volz et al., 2009; Rasmussen et al., 2011), as long as the population size is large and the sample-fraction remains negligible. The second idealization is the linear birth-death process, for which the likelihood is available in closed form (Stadler, 2010). Linearity in this context amounts to the assumption that distinct lineages do not interact: it is the resulting self-similarity of genealogies that renders the likelihood analytically tractable. Extensions of this approach develop approximations via linearization of nonlinear processes or restriction to scenarios in which population growth is nearly linear (e.g., MacPherson et al., 2021). Although the tractability of these approaches is appealing, concern naturally arises as to validity of the approximations in specific cases, the biases introduced by them, and the amount of information in data left uncaptured by these approximate methods. For this reason, there is interest in improved phylodynamic inference techniques.

What would an ideal phylodynamic inference method look like? First, it would allow us to ask the questions we wish to ask. Since the set of scientifically interesting hypotheses is not contained within the set of statistically convenient models, an ideal phylodynamic inference methodology would put no restrictions on the form of the models that it can accommodate. In particular, since nonlinearity, nonstationarity, noise, and measurement error are prominent and ubiquitous in epidemiology, such a method would accommodate nonlinear, time-inhomogeneous, stochastic transmission models. Moreover, because many of the most scientifically and practically important uncertainties concern heterogeneities in transmission rates and the susceptibility, behavior, age, and location of hosts, it would accommodate host populations structured by these and other factors. Second, it would be both statistically efficient and robust to model misspecification. In particular, it would achieve maximal statistical efficiency by being based on the exact phylodynamic likelihood, which would also facilitate objective comparison among parameterizations and models. At the same time, model predictions would be robust to small deviations from the model assumptions. Finally, an ideal inference method would be computationally efficient. If numerical computations were absolutely required for its use, these would scale well with model complexity and data volume.

Of course, there is no hope for the realization of such an ideal method. In particular, in selecting methodology, one must navigate trade-offs between statistical efficiency and robustness, and between computational expense and fidelity of model to question. With respect to these trade-offs, far more attention has been paid to date on relatively inexpensive approaches that are restricted to models that can only approximate the motivating questions to a greater or lesser extent. The present paper offers a complementary perspective: we develop mathematical expressions, and corresponding numerical algorithms, for the phylodynamic likelihood of almost arbitrarily complex models. Our focus on the exact likelihood offers maximum information capture, even as the computations it involves are somewhat delicate. Numerical examples are postponed to Appendix C, and computational scalability is discussed in §5.

While the theory presented here greatly expands the class of models for which exact likelihood is computable, it is limited to models with *discrete structure*. Thus, while structuring factors such as age, stage of infection progression, and spatial location are most naturally expressed in terms of continuous variables, to apply the results presented here, one would have to discretize these factors. We suspect that this limitation may often be relatively painless in practice, since discretely structured models have repeatedly proved their value in epidemiology. In particular, compartmental models offer great flexibility and have often been used as approximations when continuous structure leads to uncomfortably high model dimension.

To connect a model at the level of a population with genealogies based on samples taken from individual hosts, it is necessary to make assumptions about the individuals in the population. The simplest such assumption is that the individuals that are identical with respect to the population dynamics are fully statistically identical. That is, that they are *exchangeable*. In a compartmental model, this is tantamount to the assumption that the residence times of the individuals within each compartment are identically distributed, though not independent. Although exchangeability is indeed an additional assumption, it is so natural that it is frequently unrecognized as such, and one often reads statements to the effect that exchangeability of individuals is a consequence of the Markovian assumption. Nonetheless, since it adds minimal additional structure, it is the natural assumption, and the one we will make in this paper.

From the mathematical point of view, the essential difficulty in determining expressions for the likelihood of a genealogy generated by a given population process is the fact that a genealogy’s structure is determined by events occurring at widely separated times. Existing approaches to phylodynamic inference employ one of a few tricks to get around this non-locality. In particular, both coalescent- and linear birth-death process-based approaches construct arguments in reverse time that depend on restrictions on the form of the population process. The main mathematical contribution of the present paper is to show how one can reformulate the phylodynamic likelihood in such a way that all needed computations are time-localized without placing restrictive assumptions on the population process. In particular, models for which forward simulation is possible but reversal of transition densities is intractable can be treated in this way.

In the following, we take as our starting point a population model in the form of a discretely structured continuoustime Markov process. We show how such a process uniquely induces several stochastic processes in the space of genealogies. We go on to show that exact likelihoods for these genealogies can be computed by solving a filter equation. In particular, for each given population-level model there is a specific filter equation. We then show that our approach generalizes both the coalescent- and linear birth-death-process-based approaches. Indeed, the filter equations for the models on which these approaches are based can be solved to obtain the familiar likelihood formulae. In appendices, we develop the properties of filter equations generally, provide a sequential Monte Carlo algorithm that can be used when numerical integration is needed for their solution, and work out the filter equations and their solutions in two examples.

Code sufficient for the reproduction of all the figures in this paper are freely available for download at https://github.com/kingaa/structured-genealogy-process-paper. An archival version of these will be stored on Zotero upon publication of a peer-reviewed version of this paper. The open-source R package phylopomp (https://github.com/kingaa/phylopomp) implements the simulation and likelihood-computation algorithms employed here.

## 2. Mathematical preliminaries

### 2.1. Notation.

Throughout the paper, we will adopt the convention that a bold-face symbol (e.g., **X**), denotes a random element. We will be concerned with a variety of stochastic processes, in both discrete and continuous time. In both cases, we will use a subscript to indicate the time parameter: e.g., $X_{t}$ or $G_{k}$, where t takes values in the non-negative reals ${\mathbb{R}}_{+}$ and k in the non-negative integers ${\mathbb{Z}}_{+}$. In the case of continuous-time processes, we will find ourselves needing to refer to their left- or right-limits. Accordingly, if $\Phi_{t}$ is any random process, let

$${\tilde{\Phi }}_{t}≔\underset{s↑t}{lim}\Phi_{s}\;\text{and}\;{\underset{∼}{\Phi }}_{t}≔\underset{s↓t}{lim}\Phi_{s}.$$

We will typically assume that the random processes treated here are right-continuous with left limits (i.e., càdlàg, so that ${\underset{∼}{\Phi }}_{t}=\Phi_{t}$). Note that, in this case, ${\tilde{\Phi }}_{t}$ is left-continuous with right limits (càglàd).

If $\Phi_{t},t\in {\mathbb{R}}_{+}$ is a pure jump process, knowledge of its sample path is equivalent to knowledge of the number, $K_{t}$, of jumps it has taken as of time t, the jump times ${\overset{ˆ}{T}}_{k}$, and the embedded chain ${\overset{ˆ}{\Phi }}_{k}≔\Phi_{{\overset{ˆ}{T}}_{k}},k=0,\ldots ,K_{t}$. In particular, if we adopt the convention that ${\overset{ˆ}{T}}_{0}=0$ and ${\overset{ˆ}{T}}_{K_{t}+1}=t$, then $\Phi_{t}={\overset{ˆ}{\Phi }}_{k}$ for $t\in \left[{\overset{ˆ}{T}}_{k},{\overset{ˆ}{T}}_{k+1}\right)$, $k=0,\ldots ,K_{t}$.

### 2.2. Population process.

**Figure 1.**
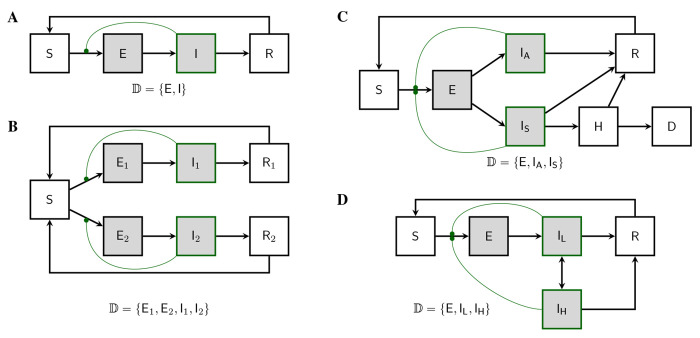
Examples of discretely-structured population models. Demes are shaded. Compartments containing infectious hosts are outlined in green. Curved green lines connect transmission rates with the compartments whose occupancies control their modulation; each such connection gives rise to a nonlinearity in the model. **(A)** An SEIRS model. Susceptible individuals (S), once infected, enter a transient incubation phase (E) before they become infectious (I). Upon recovery (R), individuals experience immunity from reinfection. If this immunity wanes, they re-enter the susceptible compartment. Pathogen lineages are to be found in hosts within the E and I compartments only. Accordingly, there are two demes: 𝔻={E,I}. If there is exactly one lineage per host, then the occupancy, $n\left(X_{t}\right)=\left(n_{E}\left(X_{t}\right),n_{I}\left(X_{t}\right)\right)$, is the integer 2-vector giving the numbers of hosts in the respective compartments. See §2.6 for definition and discussion of demes and deme occupancy. **(B)** In this four-deme model, two distinct pathogen strains compete for susceptibles. **(C)** A three-deme model according to which, after an incubation period, hosts may develop asymptomatic infection ($I_{A}$). If they do not recover, symptomatically infected hosts ($I_{S}$) can progress to hospitalization (H) and death (D). **(D)** A three-deme model with heterogeneity in transmission behavior. Contagious individuals move randomly between low-transmission ($I_{L}$) and high-transmission ($I_{H}$) behaviors.

We are motivated by the desire for exact phylodynamic inference methods for as wide a class of epidemiological models as possible. In particular, we would like to be able to formulate and parameterize an arbitrary compartmental model and to quantify its ability to explain data using likelihood. Fig. 1 depicts a few of the simplest such models in order to give a sense of the kinds of complexities that can arise. Of course, with the ability to entertain models with countably many compartments, much greater complexity is possible. In particular, one can model not only complex infection progression, but also strain structure, behavioral structure, age structure, and spatial structure using compartmental models. As is well known, one can discretize continuous structure-variables and employ the linear chain trick to accommodate non-exponential residence times. While the utility of these approximations will vary, a very wide range of model assumptions lie within the scope of the theory presented here.

We will refer to the stochastic process defined by the given model as the *population process*. We will assume that the population process is a time-inhomogeneous Markov jump process, $X_{t},t\in {\mathbb{R}}_{+}$, taking values in some space 𝕏. In earlier work (King et al., 2022), we limited ourselves to the case $𝕏={\mathbb{Z}}^{d}$, but here we assume only that 𝕏 is a complete metric measure space with a countable dense subset. The population process is completely specified by its initial-state density, $p_{0}$, and its transition rates α. In particular, we suppose that

(1) $$Prob\left[X_{0}\in ℰ\right]=\int_{ℰ}p_{0}(x)dx$$

for all measurable sets ℰ⊆𝕏. For any $t\in {\mathbb{R}}_{+},x,x^{\prime }\in 𝕏$, we think of the quantity $\alpha \left(t,x,x^{\prime }\right)$ as the instantaneous hazard of a jump from x to $x^{\prime }$. More precisely, the transition rates have the following properties:

$$\alpha \left(t,x,x^{\prime }\right)⩾0,\;\int_{𝕏}\alpha \left(t,x,x^{\prime }\right)dx^{\prime }<\infty ,$$

for all $t\in {\mathbb{R}}_{+}$ and $x,x^{\prime }\in 𝕏$ and that, as a function of time, α is càdlàg and continuous almost everywhere. Henceforth, we understand that integrals are taken over all of 𝕏 unless otherwise specified. Let $K_{t}$ be the number of jumps that X has taken by time t. We assume that $K_{t}$ is a simple counting process so that

$$\mathrm{Prob}\left[K_{t+\text{Δ}}=n+1|K_{t}=n\right]=\text{Δ}\int \alpha \left(t,x,x^{\prime }\right)\text{d}x^{\prime }+o(\text{Δ}),$$

$$\mathrm{Prob}\left[K_{t+\text{Δ}}>n+1|K_{t}=n\right]=o(\text{Δ}),$$

$$\mathrm{Prob}\left[X_{t+\text{Δ}}\in ℰ\setminus \{x\}|X_{t}=x\right]=\text{Δ}\int_{ℰ\setminus \{x\}}\alpha \left(t,x,x^{\prime }\right)\text{d}x^{\prime }+o(\text{Δ}),$$

$$\mathrm{Prob}\left[X_{t+\text{Δ}}=x|X_{t}=x\right]=1-\text{Δ}\int \alpha \left(t,x,x^{\prime }\right)\text{d}x^{\prime }+o(\text{Δ}).$$

We further assume that $X_{t}$ is *non-explosive*, i.e., that $Prob\left[K_{t}<\infty \right]=1$ for all t<∞.

### 2.3. Kolmogorov equations.

The above may be compactly summarized by stating that if v(t,x) satisfies the Kolmogorov forward equation (KFE),

(2) $$\frac{\partial v}{\partial t}(t,x)=\int v\left(t,x^{\prime }\right)\alpha \left(t,x^{\prime },x\right)dx^{\prime }-\int v(t,x)\alpha \left(t,x,x^{\prime }\right)dx^{\prime },\;v(0,x)=p_{0}(x),$$

for t∈[0,T], then $\int_{ℰ}v(t,x)dx=Prob\left[X_{t}\in ℰ\right]$ for every t∈[0,T] and measurable ℰ⊆𝕏. Eq. 2 is sometimes called the *master equation* for $X_{t}$. The adjoint form of the KFE is the Kolmogorov backward equation,

(3) $$-\frac{\partial F}{\partial s}(s,x)=\int \alpha \left(t,x,x^{\prime }\right)\left[F\left(s,x^{\prime }\right)-F(s,x)\right]dx^{\prime },\;F(T,x)=f(x).$$

If F satisfies Eq. 3 for s∈[0,T] and x∈𝕏, then $F(s,x)=𝔼\left[f\left(X_{T}\right)|X_{s}=x\right]$ for all t∈[0,T] and x∈𝕏.

### 2.4. Inclusion of jumps at deterministic times.

For modeling purposes, it is sometimes desirable to insist that certain events occur at specified times. For example, if samples are collected at specific times in such a way that the timing itself conveys no information about the process, one might wish to condition on the sampling time. We can expand the class of population models to allow for this as follows. Suppose that $S=\left\{s_{1},s_{2},\ldots ,\right\}\subset {\mathbb{R}}_{+}$ is a sequence of times. Let us postulate that, at each of these times, an event occurs at which $X_{t}$ jumps according to a given probability kernel π. In particular, for any state x∈𝕏 and measurable $ℰ\subset 𝕏,\pi \left(s_{i},x,ℰ\right)$ is the probability that the jump at time $s_{i}$ is to ℰ, conditional on ${\tilde{X}}_{s_{i}}=x$. With this notation, the KFE for the process becomes

(4) $$\frac{\partial v}{\partial t}(t,x)=\int v\left(t,x^{\prime }\right)\alpha \left(t,x^{\prime },x\right)\text{d}x^{\prime }-\int v(t,x)\alpha \left(t,x,x^{\prime }\right)\text{d}x^{\prime },\;\;\;\;t\notin S,$$

(5) $$v(t,x)\text{d}x=\int \tilde{v}\left(t,x^{\prime }\right)\pi \left(t,x^{\prime },\text{d}x\right)\text{d}x^{\prime },\;\;\;\;\;\;\;\;\;\;\;\;t\in S.$$

Note that Eq. 4 is identical to Eq. 2; we call this the *regular part* of the KFE. We refer to Eq. 5 as the *singular part* of the KFE. In this notation, $\pi \left(t,x^{\prime },dx\right)/dx$ is the density (i.e., Radon-Nikodym derivative) of π with respect to the base measure on 𝕏.

As a matter of notation, one can represent Eqs. 4 and 5 as a single equation in the form of Eq. 2. In particular, if in Eq. 2 we make the substitution

$$\alpha \left(t,x,x^{\prime }\right)dx^{\prime }\mapsto \alpha \left(t,x,x^{\prime }\right)dx^{\prime }+\sum_{s\in S}\delta (t,s)\pi \left(t,x,dx^{\prime }\right),$$

we obtain an equation which we can view as shorthand for Eqs. 4 and 5. Here, δ(t,s) is a one-sided Dirac delta function.

### 2.5. Jump marks.

It will be useful to divide the jumps of the population process $X_{t}$ into distinct categories which differ with respect to the changes they induce in a genealogy. For this purpose, we let 𝕌 be a countable set of jump *marks* such that

$$\alpha \left(t,x,x^{\prime }\right)=\sum_{u\in 𝕌}\alpha_{u}\left(t,x,x^{\prime }\right).$$

**Figure 2.**
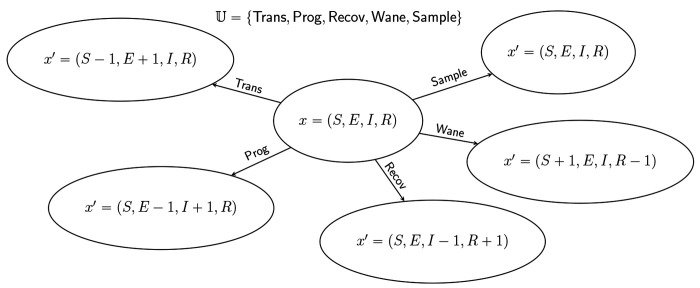
Markov state transition diagram for the SEIRS model depicted in Fig. 1A. The state, x, is characterized by four numbers, S,E,I, and R. From a given state x, there are five possible kinds of jumps $x\mapsto x^{\prime }$. Accordingly, the set, 𝕌, of jump marks has five elements. In the terminology of §3.2, each of these is of a different type: Trans (transmission) is of birth type, Prog (progression) is of migration type, Recov (recovery) is of death type, Sample (sampling) is of sample type, and Wane (loss or waning of immunity) is of neutral type. Note that, in this formulation, when a sampling event occurs, the state does not change.

Fig. 2 shows an example for which 𝕌 has five elements. In the following, sums over u are to be taken over the whole of 𝕌 unless otherwise indicated.

Let us define the *jump mark process*, $U_{t}$, to be the mark of the latest jump as of time t. As usual, we take the sample paths of $U_{t}$ to be càdlàg. Observe that $X_{t}$ and $\left(X_{t},U_{t}\right)$ are Markov processes, but that $U_{t}$ is not.

### 2.6. Demes and deme occupancy.

Our first goal in this paper is to show how a given population process induces a stochastic process on the space of genealogies. At each time, this genealogy will represent the relationships of shared ancestry among a population of lineages extant at that time. To accommodate the structure of the population, this population of lineages will itself be subdivided into discrete categories. In particular, we suppose that there are a countable set of subpopulations, within each of which individual lineages are exchangeable. We call these subpopulations *demes* and let 𝔻 be an index set for them. We note that other authors have used different terminology for the same concept, including “colony”, “type”, “state”, and “population” (Takahata, 1988; Notohara, 1990; Boskova et al., 2014; Volz & Siveroni, 2018; Barido-Sottani et al., 2020; Seidel et al., 2024; Vaughan & Stadler, 2025). Fig. 1 illustrates this concept in the context of several specific models.

We define the *deme occupancy* function $n:𝔻\times 𝕏\to {\mathbb{Z}}_{+}$ so that for $i\in 𝔻,x\in 𝕏,n_{i}(x)$ is the number of lineages in deme i when the population is in state x.

### 2.7. Examples.

The class of population models to which the theory presented here applies is very broad. In particular, it encompasses the entire class of compartmental models with time-dependent flow rates. Here, to give a sense of this breadth, we briefly describe a few models of interest.

#### SIRS model.

King et al. (2022) developed formulae for the exact likelihood of a genealogy induced by an SIRS model. The theory developed in this paper applies, but since there is only one deme in this model, it is a simple case. In Appendix C, we illustrate the theory by working it out in detail for this model.

#### SEIRS model.

Modifying the SIRS model by incorporating an incubation period results in the SEIRS model, which has two demes (Fig.1A): 𝔻={E,I}. We can take the state space to be ${\mathbb{Z}}_{+}^{4}$: the state x=(S,E,I,R) is defined by the numbers of hosts in each of the four compartments. The deme occupancy function in this case is n(x)=(E,I). In Appendix C, we work out the theory for this model in detail.

#### Two-strain competition model.

A simple model for the competition of two pathogen strains is depicted in Fig. 1B. In this model, the state vector consists of seven numbers: $x=\left(S,E_{1},E_{2},I_{1},I_{2},R_{1},R_{2}\right)$. There are four demes $\left(𝔻=\left\{E_{1},E_{2},I_{1},I_{2}\right\}\right)$ and the occupancy function is $n(x)=\left(E_{1},E_{2},I_{1},I_{2}\right)$.

#### Complex infection progression.

Fig. 1C depicts a model in which infections are heterogeneous with respect to the severity of the disease they engender. There are three demes ($𝔻=\left\{E,I_{A},I_{S}\right\}$). Asymptomatic infections ($I_{A}$) are unobservable, while symptomatic infections ($I_{S}$) may develop into hospitalized cases (H) and deaths (D).

#### Superspreading model.

Compartmental models can be used to capture heterogeneities of various kinds. Fig. 1D depicts a model with behavioral heterogeneity. There are three demes ($𝔻=\left\{E,I_{L},I_{H}\right\}$). Hosts in the low-transmitting $I_{L}$ compartment have fewer contacts than those in the high-transmitting $I_{H}$ compartment, and individuals move between these compartments as they engage in bouts of risky or cautious behavior.

#### Kingman coalescent and Moran model.

The Kingman (1982a) coalescent is one of the two foundations upon which most existing phylodynamic approaches have been constructed. It is the ancestral process for the Moran model, in which a fixed population of n lineages experiences events at times distributed according to a rate-μ Poisson process. At each such event, an individual lineage selected uniformly at random dies and is replaced by the offspring of a second randomly selected lineage. In §4.3.1, we show that the Kingman coalescent likelihood is a special case of the theory presented in this paper.

#### Linear birth-death model.

The linear birth-death process, the second basis for widely-used phylodynamic methods, is also a special case of the theory presented here. For this process, we have $𝕏={\mathbb{Z}}_{+}$ and there is a single deme. $X_{t}$ represents the size of a population and n(x)=x. In §4.3.2, we derive exact expressions for the likelihood under this model.

### 2.8. History process.

Consider the Markov process ($X_{t},U_{t}$). We define its *history process*, $H_{t}$, to be the restriction of the random function $s\mapsto \left(X_{s},U_{s}\right)$ to the interval [0,t]. Note that $H_{t}$ is itself trivially a Markov process, since it contains its own history. Alternatively, one can identify $H_{t}$ with the sequence ${\left(\left({\overset{ˆ}{T}}_{k},{\overset{ˆ}{X}}_{k},{\overset{ˆ}{U}}_{k}\right)\right)}_{k=0}^{K_{t}}$. In particular, conditional on $H_{t}$, both $X_{t}$ and $U_{t}$ are deterministic, as are $K_{t}$, the embedded chains, ${\overset{ˆ}{X}}_{k},{\overset{ˆ}{U}}_{k}$, and the point process of event times ${\overset{ˆ}{T}}_{k}$. The probability measure on the space of histories can be expressed in terms of these:

(6) $$Prob\left[{dH}_{t}\right]=p_{0}\left({\overset{ˆ}{X}}_{0}\right)d{\overset{ˆ}{X}}_{0}\prod_{k=1}^{K_{t}}\alpha_{{\overset{ˆ}{U}}_{k}}\left({\overset{ˆ}{T}}_{k},{\overset{ˆ}{X}}_{k-1},{\overset{ˆ}{X}}_{k}\right)d{\overset{ˆ}{X}}_{k}d{\overset{ˆ}{T}}_{k}exp\left(-\sum_{k=0}^{K_{t}}\int_{{\overset{ˆ}{T}}_{k}}^{{\overset{ˆ}{T}}_{k+1}}\sum_{u}\int \alpha_{u}\left(t^{\prime },{\overset{ˆ}{X}}_{k},x^{\prime }\right)dx^{\prime }dt^{\prime }\right),$$

where again, by convention, ${\overset{ˆ}{T}}_{0}=0$ and ${\overset{ˆ}{T}}_{K_{t}+1}=t$.

If H is a potential value of a history process, $H_{t}$, we define t(H) to be the right endpoint of its domain and use the notation $ev(H)≔\left\{{\overset{ˆ}{T}}_{1},\ldots ,{\overset{ˆ}{T}}_{K_{t}}\right\}\subset [0,t(H)]$ to denote the set of its jump times.

## 3. Genealogy processes

### 3.1. Genealogies.

**Figure 3.**
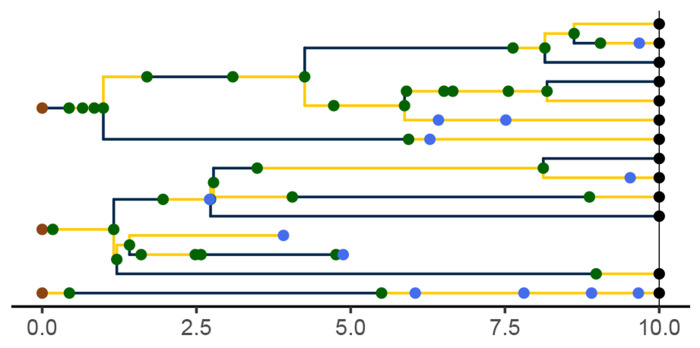
A genealogy, G, specifies the relationships of shared ancestry (via its tree-structure) and deme occupancy histories (via the coloring of its branches) of a set of lineages extant at some time t(G), as well as some samples gathered at earlier times. Here, t(G)=10 and there are two demes, 𝔻={blue, yellow}. Tip nodes, denoting extant lineages, are shown as black dots; sample nodes are shown as blue dots; internal nodes are indicated in green. Note that internal nodes occur not only at branch-points, but also inline (i.e., along branches). Wherever a lineage moves from one deme (color) to another, an internal node occurs; the converse does not necessarily hold.

A *genealogy*, G, encapsulates the relationships of shared ancestry among a set of lineages that are extant at some time $t(G)\in {\mathbb{R}}_{+}$ and perhaps a set of samples collected at earlier times (Fig. 3). A genealogy has a tree- or forest-like structure, with four distinct kinds of nodes: (i) *tip nodes*, which represent labeled extant lineages; (ii) *internal nodes*, which represent events at which lineages diverged and/or moved from one deme to another; (iii) *sample nodes*, which represent labeled samples; and (iv) root nodes, at the base of each tree. Each node a is associated with a specific time, t(a). In particular, if a is a tip node in G, then t(a)=t(G); if a is a sample node, then t(a)⩽t(G) is the time at which the sample was taken. Moreover, if node a is ancestral to node $a^{\prime }$, then $t(a)⩽t\left(a^{\prime }\right)$ and $t\left(a^{\prime }\right)-t(a)$ is the distance between a and $a^{\prime }$ along the genealogy. Without loss of generality we assume that t(a)=0 for all root nodes a. We let ev(G) denote the set of all internal and sample node-times of the genealogy G; we refer to these as *genealogical event times*.

Importantly, a genealogy informs us not only about the shared ancestry of any pair of lineages, but also about where in the set of demes any given lineage was at all times. Accordingly, we can visualize a genealogy as a graph, the nodes and edges of which are painted with a distinct color for each deme (Fig. 3). Note that a genealogy will in general have *branch-point nodes*, i.e., internal nodes with more than one descendant, but may also have internal nodes with only one descendant. We refer to such nodes as *inline nodes*. These occur whenever the color changes along a branch, but can also occur without a color-change.

Formally, we define a genealogy, G, to be a triple, (T,Z,Y), where $T=t(G)\in {\mathbb{R}}_{+}$ is the *genealogy time*, Z specifies the genealogy’s *tree structure*, and Y gives the *coloring*. In particular, let 𝕃 be a countable set of labels-referring to samples and/or extant lineages—and let partit (𝕃) be the set of all collections of finite, mutually-disjoint subsets of 𝕃. That is, an element z∈partit(𝕃) is a partition of the finite set ⋃z⊆𝕃. Partition *fineness* defines a partial order on partit(𝕃). Specifically, for $z,z^{\prime }\in partit(𝕃)$, we say $z≼z^{\prime }$ if and only if for every $b^{\prime }\in z^{\prime }$ there is b∈z such that $b⊇b^{\prime }$. The tree structure of G is a càdlàg map Z:[0,T]→partit(𝕃) that is monotone in the sense that $t_{1}⩽t_{2}$ implies $Z_{t_{1}}≼Z_{t_{2}}$. An element $b\in Z_{t}$ is a set of labels; it represents the branch of the tree that bears the corresponding lineages. We use the notation ev(Z) to denote the set of times at which Z is discontinuous. Note that ev(Z) includes the times of all tip, sample, and branch-point nodes, but excludes root and non-sample inline nodes. Therefore, ev(Z)⊆ev(G).

The third element of G specifies the coloring of branches and locations of tip, sample, and internal nodes (including inline nodes). Mathematically, if G=(T,Z,Y), then Y is a càdlàg function that maps each point on the genealogy to a deme and a non-negative integer. In particular, if t∈[0,T] and a is the label of any tip or sample node, $Y_{t}(a)=\left(Y_{t}^{d}(a),Y_{t}^{m}(a)\right)\in 𝔻\times {\mathbb{Z}}_{+}$, where $Y_{t}^{d}(a)$ is the deme in which the lineage of a is located at time t and $Y_{t}^{m}(a)$ is the number of internal or sample nodes encountered along the lineage of a in going from time 0 to time t. In particular, $Y_{t}^{m}(a)$ is a simple counting process, with $Y_{0}^{m}(a)=0$ for all a. Since $a,a^{\prime }\in b\in Z_{t}$ implies $Y_{t}(a)=Y_{t}\left(a^{\prime }\right)$, one can equally well think of $Y_{t}$ as a map $Z_{t}\to 𝔻\times {\mathbb{Z}}_{+}$. Given a tree Z, we let Y(Z) denote the set of colorings Y that are compatible with Z. We moreover define $Y_{t}(Z)≔\left\{Y_{t}|Y\in Y(Z)\right\}$. Formally speaking, Y(Z) is a fiber bundle over Z, each $Y_{t}(Z)$ being a fiber.

It will sometimes be convenient to make use of notation whereby the tree structure of genealogy G is $G^{Z}$ and the coloring is $G^{Y}$, so that $G=\left(t(G),G^{Z},G^{Y}\right)$.

### 3.2. Event types.

To see how events in the population process leave a trace in the genealogy, we assume that, at each jump in the population process, a corresponding change occurs in the genealogy, according to whether lineages branch, die, move between demes, or are sampled. For this purpose, there are five distinct *pure types* of events:

- *Birth-type events* result in the branching of one or more new lineages from an existing lineage. Examples of birth-type events include transmission events, speciations, and actual births. Importantly, we assume that all new lineages arising from a birth event share the same parent and that at most one birth event occurs at a time, almost surely. While it is possible to relax this assumption, the resulting notational complexities are nontrivial, so we postpone consideration of this more general case.
- *Death-type events* result in the extinction of one or more lineages. Examples include recovery from infection, death of a host, and species extinctions. We allow for the possibility that multiple lineages, potentially in multiple demes, die simultaneously.
- *Migration-type events* result in the movement of a lineage from one deme to another. Spatial movements, changes in host age or behavior, and progression of an infection can all be represented as migration-type events. In a migration-type event, one or more lineages within exactly one deme may move simultaneously, and they may move to different demes. Again, while it possible to relax the assumption that exactly one deme is the source of all migrating lineages within any one event, we postpone consideration of this case for the present.
- *Sample-type events* result in the collection of a sample from a lineage. We allow for the possibility that multiple samples are collected simultaneously, though we require that, in this case, each extant lineage is sampled at most once and that all sampled lineages be in the same deme. It is possible to allow for simultaneous sampling of multiple demes but, again, we postpone consideration of this case to ease the exposition.
- *Neutral-type events* result in no change to any of the lineages.

Fig. 2 depicts an example with jumps of all five pure types. It is not necessary that an event be of a pure type; *compound events* partake of more than one type. For example, a sample/death-type event, in which a lineage is simultaneously sampled and removed, has been employed (Leventhal et al., 2014), as have birth/death events in which one lineage reproduces at the same moment that another dies (e.g., the Moran (1958) process). In combining the pure types, the main restriction is that, at any compound event, all parent lineages must be drawn from the same deme. Beyond this, the theory presented here places few restrictions on the complexity of the events that can occur by combining events of the various pure types.

### 3.3. Genealogy process.

We now show how a given population process induces a stochastic process, $G_{t}$, on the space of genealogies. In the case of unstructured population processes (i.e., those having a single deme), King et al. (2022) gave a related construction that is equivalent to the one presented here.

**Figure 4.**
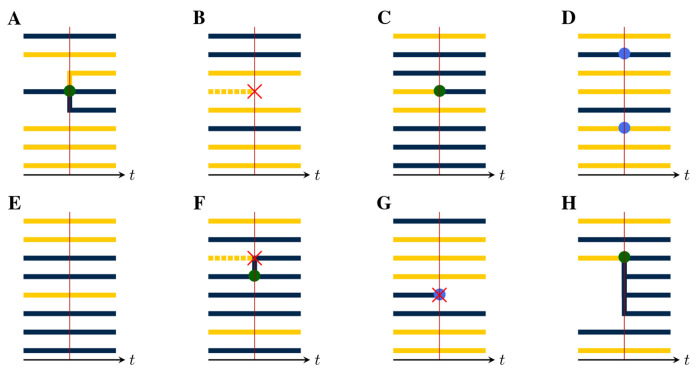
Event types differ by their effects on the genealogy. This can be seen by examining the local structure of the genealogy in the neighborhood of a jump. **(A)** A birth-type jump results in the branching of one or more child lineages from the parent. There can be only one parent, though the demes of the child lineages may differ from that of their parent. Here, a parent of the blue deme sires one child lineage in each of the blue and yellow demes. The *production* of an event is an integer vector, with one entry for each deme. The production of this event is therefore $r=\left(r_{\text{blue}},r_{\text{yellow}}\right)=(2,1)$. The *deme occupancy* of an event is the number of lineages in each deme just to the right of the event. The deme occupancy at this event is therefore $n=\left(n_{\text{blue}},n_{\text{yellow}}\right)=(3,5)$. **(B)** A death-type event causes the extinction of a lineage. Since internal nodes without children are recursively removed, the affected branch is dropped. The production of this event is r=(0,0) and the deme occupancy is n=(3,4). **(C)** A migration-type event results in the movement of one or more lineages from one deme to another. Here, one lineage moves from the yellow to the blue deme. The production of this event is r=(1,0), i.e., the production is 1 for the blue deme and 0 for the yellow. The deme occupancy is n=(6,2). **(D)** In a sample-type event, one or more sample nodes (blue circles) are inserted. Here, there are two samples, one in each of the blue and yellow demes. Accordingly, r=(1,1) and n=(2,6). **(E)** A neutral-type event has no effect on the genealogy and zero production in all demes: r=(0,0),n=(5,3). **(F)** The theory presented here allows for compound events. As an example, here a birth/death-type event occurs, wherein one yellow lineage is extinguished and a blue lineage simultaneously sires a blue child. For this event, we have r=(2,0) and n=(6,2). **(G)** Here, a compound sample/death-type event with r=(0,0) and n=(2,5) occurs. A blue lineage is sampled and simultaneously extinguished. Note that recursive removal does not occur, since sample nodes are never removed. **(H)** A compound birth/migration-type event with r=(4,0) and n=(6,2).

At each jump in the population process, a change is made to the genealogy, according to the mark, u, of the jump (Fig. 4). In particular:

- If u is of birth-type (Fig. 4A), it results in the creation of one new internal node, call it b. A tip node, a, of the appropriate deme is chosen with uniform probability from among those present and b is inserted so that its ancestor is that of a, while a takes b as its ancestor. One new tip node, of the appropriate deme, is created for each of the children, all of which take b as their immediate ancestor.
- If u is of death-type (Fig. 4B), one or more tip nodes of the appropriate demes are selected with uniform probability from among those present. These are deleted. Next, non-sample internal nodes without children are recursively removed. Sample nodes are never removed.
- At a migration-type event (Fig. 4C), the appropriate number of migrating lineages are selected at random with uniform probability, from among those present in the appropriate deme. For each selected lineage, one new branch node is inserted between the selected tip node and its ancestor. The color of the descendant branch changes accordingly.
- At a sample-type event (Fig. 4D), the appropriate number of sampled lineages are selected at random from among the tip nodes, with uniform probability according to deme. One new sample node is introduced for each selected lineage: each is inserted between a selected tip node and its ancestor.
- At a neutral-type event (Fig. 4E), no change is made to the genealogy.
- Finally, events of compound type (e.g., Fig. 4F–H) are accommodated by combining the foregoing rules.

In each of these events, the new node or nodes that are introduced have node-times equal to the time of the jump.

#### 3.3.1. Emergent lineages and production.

At each event, the lineages which descend from a node inserted at that event are said to *emerge* from the event. Thus, after a birth-type event, the emerging lineages include all the new offspring as well as the parent. Likewise, at pure migration- or sample-type events, each migrating or sampled lineage emerges from the event. At pure death-type events, no lineages emerge. In general, at an event of mark u, there are $r_{i}^{u}$ emergent lineages in deme i. We require that $r_{i}^{u}$ be a constant, for each u and i. Thus there is a function $r:𝕌\times 𝔻\to {\mathbb{Z}}_{+}$, such that $r_{i}^{u}$ lineages of deme i emerge from each event of mark u. Since, in applications, one is free to expand the set of jump-marks 𝕌 as needed, this is not a restriction on the models that the theory can accommodate. We say $r^{u}≔{\left(r_{i}^{u}\right)}_{i\in 𝔻}$ is the *production* of an event of mark u. Note that the lineages that die as a result of an event do not count in the production but that a parent lineage that survives the event does count.

#### 3.3.2. Conditional independence and exchangeability.

Application of these rules at each jump of $X_{t}$ constructs a chain of genealogies ${\overset{ˆ}{G}}_{k}$. In particular, at each jump-time ${\overset{ˆ}{T}}_{k}$, the genealogy ${\overset{ˆ}{G}}_{k-1}$ is modified according to the jump-mark ${\overset{ˆ}{U}}_{k}$ to yield ${\overset{ˆ}{G}}_{k}$. We view ${\overset{ˆ}{G}}_{k},k=0,1,2,\ldots$, as the embedded chain of the continuous-time genealogy process $G_{t}$. It is very important to note that, conditional on (${\overset{ˆ}{X}}_{k},{\overset{ˆ}{U}}_{k}$), the number of parents and number of offspring in each deme is determined and the random choice of which lineages die, migrate, are sampled, or sire offspring is independent of these choices at any other times and independent of (${\overset{ˆ}{X}}_{j},{\overset{ˆ}{U}}_{j}$) for all j≠k. Moreover, by assumption, the lineages within each deme are exchangeable: any lineage within a deme is as likely as any other lineage in that deme to be selected as a parent or for death, sampling, or migration. Finally, note that $G_{t}$ does not have the Markov property, though $\left(X_{t},U_{t},G_{t}\right)$ and $\left(X_{t},G_{t}\right)$ do. In passing, we observe that, if instead of dropping tip nodes at death events we were to retain them as we do samples, the resulting genealogy—which we might call the *complete genealogy*—would have the Markov property.

### 3.4. Pruned and obscured genealogies.

**Figure 5.**
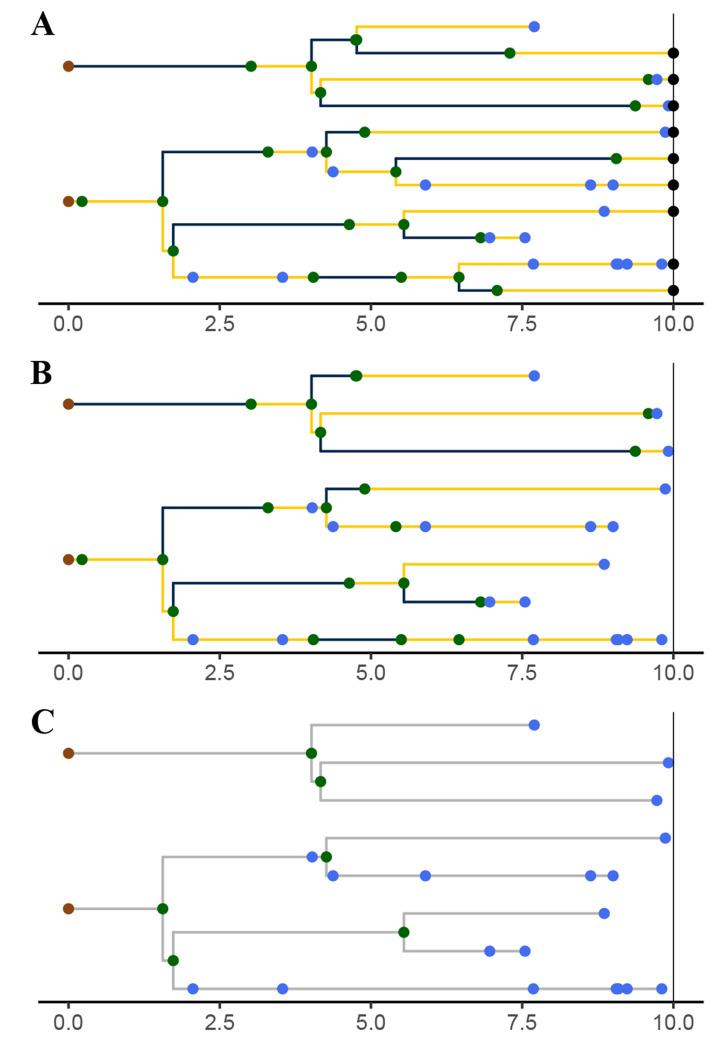
Unpruned, pruned, and obscured genealogies from a single realization of the genealogy process induced by the SEIRS model depicted in Figs. 1 and 2. **(A)** A realization of the unpruned genealogy process $G_{t}$ is shown at t=10. Tip nodes, corresponding to lineages alive at time t=10 are indicated with black points. Blue points represent samples; green points, internal nodes. Branches are colored according to the deme in which the corresponding lineage resided at that point in time: blue denotes E and yellow, I. **(B)** The genealogy is *pruned* by deleting all tip nodes and then recursively pruning away childless internal nodes. Sample nodes are never removed. **(C)** A pruned genealogy is *obscured* by effacing all deme information from lineage histories: the colors are erased, as are all inline nodes. See the text (§§3.1, 3.4.1, and 3.4.4) for more detail.

The process just described yields a genealogy that relates all extant members of the population, and all samples. Moreover, it details each lineage’s complete history of movement through the various demes. However, the data we ultimately wish to analyze will be based only on samples. Nor, in general, will the histories of deme occupancy be observable. A generative model must account for this loss of information. We therefore now describe how genealogies are *pruned* to yield sample-only genealogies and then *obscured* via the erasure of color from their branches (Fig. 5).

#### 3.4.1. Pruned genealogy.

Given a genealogy G, one obtains the *pruned genealogy*, P=prune(G) by first dropping every tip node and then recursively dropping every childless internal node (Fig. 5A–B). In a pruned genealogy only internal and sample nodes remain, and sample nodes are found at all of the leaves and possibly some of the interior nodes of the genealogy. Observe that a pruned genealogy is a colored genealogy: it retains information about where among the demes each of its lineages was through time (Fig. 5B). Note also that a pruned genealogy P is characterized by its time, t(P) and the functions $P^{Y}$ and $P^{Z}$ just as an unpruned genealogy is. Crucially, since it contains within itself all of its past history, the pruned genealogy process $P_{t}=\mathrm{prune}\left(G_{t}\right)$ is Markov, even though the unpruned genealogy process, $G_{t}$, is not.

#### 3.4.2. Lineage count and saturation.

**Figure 6.**
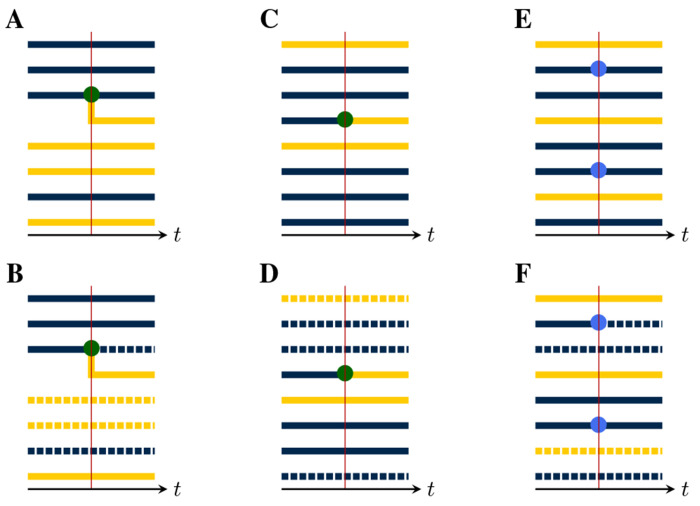
Lineage count and saturation. Each panel shows the neighborhood of a single event in the unpruned genealogy (top row) and the corresponding pruned genealogy (bottom row). Pruning consists of the removal of all branches that are not ancestral to some sample. In the bottom row of panels, pruned branches are indicated using broken lines. **(A)** A birthtype event with production $r=\left(r_{\text{blue}},r_{\text{yellow}}\right)=(1,1)$ occurs. **(B)** Suppose that pruning results in the removal of the dashed lineages. Then the lineage count at this event-time is $\ell =\left(\ell_{\text{blue}},\ell_{\text{yellow}}\right)=(2,2)$. The saturation is s=(0,1) since only a single, yellow lineage emerges from the event. **(C)** A migration-type event with production r=(0,1) occurs. **(D)** After pruning, ℓ=(2,2) and s=(0,1). **(E)** A sample-type event occurs in which two blue lineages are sampled (production =(2,0)). **(F)** After pruning, ℓ=(2,2) and s=(1,0). Observe that in panels B and D, the local structures of the pruned genealogies are identical, though they arise from events of different type.

In the following, we will find that we need to count the deme-specific numbers of lineages present in a given pruned genealogy at a given time. Accordingly, suppose P=(T,Z,Y) is a pruned genealogy and suppose t∈[0,T]. Let $\ell_{i}$ denote the number of lineages in deme i at time t and $\ell ≔{\left(\ell_{i}\right)}_{i\in 𝔻}\in {\mathbb{Z}}_{+}^{𝔻}$. Clearly, ℓ depends only $Y_{t}$. Therefore, we can define ℓ as a function such that, whenever P=(T,Z,Y) is a pruned genealogy, $\ell \left(Y_{t}\right)$ is the vector of deme-specific lineage counts at time t. We refer to ℓ as the *lineage-count* function (cf. Fig. 6).

We will also have occasion to refer to the deme-specific number of lineages emerging from a given event. In particular, given a node time t in a pruned genealogy P=(T,Y,Z), the number $s_{i}$ of lineages of deme i emerging from all nodes with time t is well defined and we can write $s≔{\left(s_{i}\right)}_{i\in 𝔻}$. Like the lineage-count function, s depends only on the local structure of P. However, s depends not only on $Y_{t}$, but also on ${\tilde{Y}}_{t}$. Thus, we can define the *saturation* function such that, whenever P=(T,Y,Z) is a pruned genealogy, $s\left({\tilde{Y}}_{t},Y_{t}\right)$ is the integer vector of deme-specific numbers of emerging lineages at time t. Fig. 6 illustrates.

#### 3.4.3. Compatibility.

Suppose P is a pruned genealogy, with t(P)=T and t∈[0,T]. The local structure of P at t is, in general, compatible with only a subset of the possible jumps 𝕌. For example, if there is a branch node or a sample node at t, then it is compatible only with birth-type or sample-type jumps, respectively. Similarly, if a lineage moves from deme i to deme $i^{\prime }$ at t, then u must be either of $i\to i^{\prime }$ migration type or of a birth type with parent in i and $r_{i^{\prime }}^{u}>0$. To succinctly accommodate all possibilities, let us introduce the indicator function Q such that Q=1 if the local genealogy structure—which is captured by the values of $P^{Y}$ just before and at t—is compatible with an event of type u and Q=0 otherwise. That is, $Q_{u}\left(y,y^{\prime }\right)=1$ if and only if there is a feasible genealogy, G=(T,Z,Y), and history, H, and a t∈[0,T] such that, given $G_{T}=G$ and $H_{T}=H$, we have $U_{t}=u,{\tilde{Y}}_{t}=y$, and $Y_{t}=y^{\prime }$. We refer to Q as the *compatibility indicator*.

#### 3.4.4. Obscured genealogy.

The *obscured genealogy* is obtained by discarding all information about demes and events not visible from the topology of the tree alone (Fig. 5B–C). In particular, if P=(T,Z,Y) is a pruned genealogy, we write obs(P)=(T,Z) to denote the obscured genealogy.

### 3.5. Binomial ratio.

The statement of the theorems to come is made easier with the following definition. For $n,r,\ell ,s\in {\mathbb{Z}}_{+}^{𝔻}$, we define the *binomial ratio*

nℓrs≔Πi∈𝔻ni−ℓiri−siΠi∈𝔻niri,if∀ini⩾ℓi,ri⩾si⩾0,0,otherwise.

where ab=a!b!(a−b)! is the binomial coefficient. Observe that $\left(\begin{array}{ll} n & \ell \\ r & s \end{array}\right)\in [0,1]$. Moreover, in consequence of the Chu-Vandermonde identity, we have

∑s∈ℤ+𝔻nℓrsℓs=1,

whenever $n_{i}⩾\left\{\ell_{i},r_{i}\right\}⩾0$ for all i.

## 4. Results

### 4.1. Likelihood for pruned genealogies.

Our first result will be an expression for the likelihood of a given pruned genealogy given the history of the population process.

**Theorem 1.** *Suppose* P=(T,Z,Y) *is a given pruned genealogy. Define*

(7) $$\phi_{u}\left(x,y,y^{\prime }\right)≔\left(\begin{array}{cc} n(x) & \ell \left(y^{\prime }\right)\\ r^{u} & s\left(y,y^{\prime }\right) \end{array}\right)Q_{u}\left(y,y^{\prime }\right),$$

where n is the deme occupancy (§2.6), $r^{u}$ is the production (§3.3.1), ℓ and s are the lineage-count and saturation functions, respectively (§3.4.2), Q is the compatibility indicator (§3.4.3), and the binomial ratio is as defined in §3.5. Then

$$Prob\left[P_{T}=P|H_{T}=H\right]=𝟙\{ev(H)⊇ev(P)\}\prod_{t\in ev(H)}\phi_{U_{t}}\left(X_{t},{\tilde{Y}}_{t},Y_{t}\right).$$

The proof is given in Appendix A.

Next, we show how the likelihood of a pruned genealogy, unconditional on the history, can be computed. For this, we use the filter equation technology developed in Appendix B. In particular, the following theorem follows immediately from Theorem 1 and Lemma B2.

**Theorem 2.** *Suppose that* P=(T,Z,Y) *is a given pruned genealogy. Suppose that* w=w(t,x) *is càdlàg in* t *and satisfies the initial condition* $w(0,x)=p_{0}(x)$ *and the filter equation*

(8) $$\begin{array}{ll} \frac{\partial w}{\partial t}(t,x)=\underset{u}{\Sigma }\int w\left(t,x^{\prime }\right)\alpha_{u}\left(t,x^{\prime },x\right)\phi_{u}\left(x,{\tilde{Y}}_{t},Y_{t}\right)dx^{\prime }-\underset{u}{\Sigma }\int w(t,x)\alpha_{u}\left(t,x,x^{\prime }\right)dx^{\prime }, & t\notin ev(P),\\ \;w(t,x)=\underset{u}{\Sigma }\int \tilde{w}\left(t,x^{\prime }\right)\alpha_{u}\left(t,x^{\prime },x\right)\phi_{u}\left(x,{\tilde{Y}}_{t},Y_{t}\right)dx^{\prime }, & t\in ev(P), \end{array}$$

*where* ϕ *is defined in* Eq. 7. *Then the likelihood of* P *is*

ℒ=∫w(T,x)dx.

Alternatively, one can express the likelihood of a pruned genealogy using the adjoint form of the filter equation (see Appendix B.3).

**Corollary 3.** *If* P=(T,Z,Y) *is a given pruned genealogy and* F=F(s,x) *is càglàd in* s *and satisfies the final condition* $\underset{∼}{F}(T,x)=1$ *and the adjoint filter equation*

(9) $$\begin{array}{ll} -\frac{\partial F}{\partial s}(s,x)=\underset{u}{\Sigma }\int {\tilde{\alpha }}_{u}\left(s,x,x^{\prime }\right)\left[\phi_{u}\left(x^{\prime },{\text{Y}}_{s},{\text{Y}}_{s}\right)F\left(s,x^{\prime }\right)-F(s,x)\right]\;\text{d}x^{\prime }, & s\notin \mathrm{ev}(\text{P}),\\ \;F(s,x)=\underset{u}{\Sigma }\int \alpha_{u}\left(s,x,x^{\prime }\right)\phi_{u}\left(x^{\prime },{\text{Y}}_{s},{\text{Y}}_{s}\right)\underset{∼}{F}\left(s,x^{\prime }\right)\text{d}x^{\prime }, & s\in \mathrm{ev}(\text{P}), \end{array}$$

*where* 0⩽s⩽T and ϕ *is defined in* Eq. 7, *then the likelihood of* P *is*

$$ℒ=\int F(0,x)p_{0}(x)dx.$$

*Remark* 1. Note that the likelihood given by the foregoing expressions is the likelihood of a given genealogy *in which the samples are ordered* and that, while this order should be compatible with time-ordering, it may not be completely determined by it. In particular, if n samples are taken at one time, there are n! possible orderings of the samples, each of which has an identical likelihood. In consequence, the likelihood given above will differ by a factor of n! from the likelihood computed ignoring ordering of samples. From the standpoint of parameter estimation, this is immaterial, since factors that depend only on the data drop out of the score function. This consideration is relevant, however, in the context of model comparison.

### 4.2. Likelihood for obscured genealogies.

Our next result concerns the likelihood of a given obscured genealogy conditional on the history.

**Theorem 4.** *Suppose that* (T,Z) *is a given obscured genealogy. Let* q *and* π *be probability kernels, such that for all* x∈𝕏 and $y\in Y_{0}(Z)$,

$$q(x,y)⩾0,\;\sum_{y\in Y_{0}(Z)}q(x,y)=1,$$

*and, for all* $u\in 𝕌,t\in {\mathbb{R}}_{+},x,x^{\prime }\in 𝕏,y,y^{\prime }\in Y_{t}(Z)$,

$$\pi_{u}\left(t,x,x^{\prime },y,y^{\prime }\right)⩾0,\;\sum_{y^{\prime }\in Y_{t}(Z)}\pi_{u}\left(t,x,x^{\prime },y,y^{\prime }\right)=1.$$

*Suppose moreover that* $\pi_{u}\left(t,x,x^{\prime },y,y^{\prime }\right)>0$ *whenever* $\alpha_{u}\left(t,x,x^{\prime }\right)Q_{u}\left(y,y^{\prime }\right)>0$ *and that* q(x,y)>0 *whenever* $Prob\left[P_{0}^{Y}=y|X_{0}=x\right]>0$. *Then there is a stochastic jump process* $y_{t}$ *with sample paths in* Y(Z) *such that* $\left(X_{t},U_{t},y_{t}\right)$ *is Markov and*

$$Prob\left[P_{T}^{Z}=Z|H_{T}=H\right]=𝟙\{ev(H)⊇ev(Z)\}𝔼\;\left[\frac{1}{q\left(X_{0},y_{0}\right)}\prod_{t\in ev(H)}\frac{\phi_{U_{t}}\left(X_{t},{\tilde{y}}_{t},y_{t}\right)}{\pi_{U_{t}}\left(t,{\tilde{X}}_{t},X_{t},{\tilde{y}}_{t},y_{t}\right)}\right],$$

*where* ϕ *is defined in* Eq. 7 *and the expectation is taken over the sample paths of* $y_{t}$.

*Proof*. First, observe that, since obs is a deterministic operator,

(10) $$Prob\left[P_{T}^{Z}=Z|H_{T}=H\right]=𝔼\left[𝟙\left\{P_{T}^{Z}=Z\right\}|H_{T}=H\right].$$

Our strategy will be to evaluate Eq. 10 using a change of measure: we will propose pruned genealogies compatible with Z as sample paths from a stochastic process driven by $X_{t}$ and evaluate the the expectation in Eq. 10 by a suitably weighted expectation over these paths. Conditional on $H_{T}=H$, the initial distribution q and probability kernel π generate a Markov chain, ${\overset{ˆ}{y}}_{k}$ such that

$$Prob\left[{\overset{ˆ}{y}}_{0}|H_{T}=H\right]=q\left(X_{0},{\overset{ˆ}{y}}_{0}\right),\;Prob\left[{\overset{ˆ}{y}}_{k}|{\overset{ˆ}{y}}_{k-1},H_{T}=H\right]=\pi_{{\overset{ˆ}{U}}_{k}}\left({\overset{ˆ}{T}}_{k},{\overset{ˆ}{X}}_{k-1},{\overset{ˆ}{X}}_{k},{\overset{ˆ}{y}}_{k-1},{\overset{ˆ}{y}}_{k}\right).$$

The required process $y_{t}$ is the unique càdlàg process with event times ${\overset{ˆ}{T}}_{k}$ and ${\overset{ˆ}{y}}_{k}$ as its embedded chain. This construction of $y_{t}$ obviously guarantees that ev(H)⊇ev(y)⊇ev(Z) and that $\left(X_{t},U_{t},y_{t}\right)$ is Markov.

Now, for y∈Y(Z), let us define C(y)=(T,Z,y). Then, by construction, obs (C(y))=(T,Z) and, conversely, for every pruned genealogy P satisfying t(P)=T and $P^{Z}=Z,C\left(P^{Y}\right)=P$. Moreover, the conditions on the kernels q and π guarantee that, if $Prob\left[P_{T}=P|H_{T}=H\right]>0$ and $P^{Z}=Z$, then $Prob\left[y=P^{Y}|H_{T}=H\right]>0$. We therefore have that

$$Prob\;\left[P_{T}^{Z}=Z|H_{T}=H\right]=𝔼\;\left[\frac{Prob\left[P_{T}=C(y)|H_{T}=H\right]}{\pi (y|H)}\right],$$

the expectation being taken with respect to the random process y. Here, by definition,

$$\pi (y|H)=q\left(X_{0},y_{0}\right)\prod_{t\in ev(H)}\pi_{U_{t}}\left(t,{\tilde{X}}_{t},X_{t},{\tilde{y}}_{t},y_{t}\right).$$

The result then follows from Theorem 1.

Note that, since $Y_{t}(Z)$ is finite, it is permissible, for example, to choose q and π to be uniform.

The final result shows how to compute the likelihood of an obscured genealogy. It is an immediate consequence of Theorem 4 and Lemma B2.

**Theorem 5.** *Let* V=(T,Z) *be a given obscured genealogy. Then there are probability kernels* q *and* π *as in Theorem 4 such that if*

$$\beta_{u}\left(t,x,x^{\prime },y,y^{\prime }\right)=\alpha_{u}\left(t,x,x^{\prime }\right)\pi_{u}\left(t,x,x^{\prime },y,y^{\prime }\right),\;\Psi_{u}\left(t,x,x^{\prime },y,y^{\prime }\right)=\frac{\phi_{u}\left(x^{\prime },y,y^{\prime }\right)}{\pi_{u}\left(t,x,x^{\prime },y,y^{\prime }\right)},$$

and if w=w(t,x,y) satisfies the initial condition $w(0,x,y)=p_{0}(x)𝟙\{q(x,y)>0\}$ and the filter equation

$$\begin{array}{ll} \frac{\partial w}{\partial t}=\underset{uy^{\prime }}{\Sigma }\int w\left(t,x^{\prime },y^{\prime }\right)\beta_{u}\left(t,x^{\prime },x,y^{\prime },y\right)\Psi_{u}\left(t,x^{\prime },x,y^{\prime },y\right)dx^{\prime }-\underset{uy^{\prime }}{\Sigma }\int w(t,x,y)\beta_{u}\left(t,x,x^{\prime },y,y^{\prime }\right)dx^{\prime }, & t\notin ev(Z),\\ w(t,x,y)=\underset{uy^{\prime }}{\Sigma }\int \tilde{w}\left(t,x^{\prime },y^{\prime }\right)\beta_{u}\left(t,x^{\prime },x,y^{\prime },y\right)\Psi_{u}\left(t,x^{\prime },x,y^{\prime },y\right)dx^{\prime }, & t\in ev(Z), \end{array}$$

then the likelihood of V is

$$ℒ=\sum_{y\in Y_{T}(Z)}\int w(T,x,y)dx.$$

Lemma B3 shows how this can be computed via sequential Monte Carlo. As before, there is also an adjoint form of Theorem 5, to wit:

**Corollary 6.** *Let* V=(T,Z) *be a given obscured genealogy and let* β and Ψ *be defined as in Theorem 5. Suppose* F *is càglàd and satisfies the final condition* $\underset{∼}{F}(T,x,y)=1$ *for all* $x\in 𝕏,y\in Y_{T}(Z)$ *and also the adjoint filter equation*

$$\begin{array}{ll} -\frac{\partial F}{\partial s}=\underset{uy^{\prime }}{\Sigma }\int {\tilde{\beta }}_{u}\left(s,x,x^{\prime },y,y^{\prime }\right)\left[\Psi_{u}\left(s,x,x^{\prime },y,y^{\prime }\right)F\left(s,x^{\prime },y^{\prime }\right)-F(s,x,y)\right]\text{d}x^{\prime }, & s\notin \mathrm{ev}(\text{Z}),\\ \;F(s,x,y)=\underset{uy^{\prime }}{\Sigma }\int \beta_{u}\left(s,x,x^{\prime },y,y^{\prime }\right)\Psi_{u}\left(s,x,x^{\prime },y,y^{\prime }\right)\underset{˜}{F}\left(s,x^{\prime },y^{\prime }\right)\text{d}x^{\prime }, & s\in \mathrm{ev}(\text{Z}), \end{array}$$

*for* 0⩽s⩽T. *Then the likelihood of* V is

$$ℒ=\sum_{y\in Y_{0}(Z)}\int F(0,x,y)p_{0}(x)dx.$$

### 4.3. Special cases.

#### 4.3.1. Moran model and the Kingman coalescent.

In the Moran model (Moran, 1958; King et al., 2020), events occur according to a rate −μ Poisson process. At each event, a compound birth-death jump occurs so that the population size, n, remains constant (cf. Fig. 4F). If we let $X_{t}$ be the number of events that have occurred by time t, then $X_{t}$ is a simple counting process, which we can use to define the state of the population process. Its KFE is then

$$\frac{\partial v}{\partial t}=\mu v(t,x-1)-\mu v(t,x),\;v(0,x)=\left\{\begin{array}{ll} 1, & x=0,\\ 0, & x>0. \end{array}\right.$$

Since there is only a single deme, and since nothing depends on the state, in writing the corresponding filter equation (Theorem 5), we can take w to be independent of both x and y.

In the classical case (Kingman, 1982a), m samples are taken simultaneously at a single time, T. Then, if B is the set of branch-times and ℓ(t) is the number of lineages in the genealogy at time t, upon summing over x and y, the filter equation reads

(11) w(0)=1,∂w∂t=μw(t)1−(ℓ(t)2)(n2)−μw(t),t∉B,w(t)=μ(n2)w˜(t),t∈B.

Integrating Eqs. 11 and taking logarithms yields

(12) logℒ=|B|logμ(n2)−μ(n2)∑i=1mi2si,

where the $s_{i}≔\int 𝟙\{\ell (t)=i\}dt$ are the durations of the *coalescent intervals*, i.e., intervals between successive branch-points. We recognize Eq. 12 as the expression for the Kingman (1982a) coalescent likelihood (e.g., Wakeley, 2009).

More generally, if in addition samples are taken according to a rate-ψ Poisson process such that the set of sample-times in the genealogy is $S=S_{0}\cup S_{1}$, where $S_{0},S_{1}$ are the sets of times of terminal and inline samples, respectively, then the filter equation reads

(13) w(0)=1,∂w∂t=−μ(ℓ(t)2)(n2)w(t),t∉S∪B,w(t)w˜(t)=μ(n2),t∈B,ψ1−ℓ(t)n,t∈S0,ψn,t∈S1.

Integrating Eqs. 13 yields

(14) logℒ−|S|logψ=∑t∈S0log1−ℓ(t)n−S1logn+|B|logμ(n2)−μ(n2)∑i=1∞i2si,

in agreement with the result of the very different derivation of King et al. (2020).

#### 4.3.2. Linear birth-death model.

In this model, the state variable, $X_{t}$, is the size of a population at time t. All individuals face the same per-capita birth and death rates, which are λ and μ, respectively. Accordingly, the Kolmogorov backward equation for this model is

$$-\frac{\partial F}{\partial s}=\lambda x[F(s,x+1)-F(s,x)]+\mu x[F(s,x-1)-F(s,x)].$$

Stadler (2010) considered the case where samples are taken through time at a uniform per-capita rate ψ and lineages extant at time T are sampled with probability ρ. Let B denote the set of branch-times in an observed genealogy, let $S_{0}$ and $S_{1}$ be, respectively, the sets of terminal and inline sample times prior to time T, and suppose n lineages are sampled at time T. Since there is only a single deme, and since nothing depends on the genealogy coloring, in writing the corresponding filter equation, we can apply Corollary 6 and take F to be independent of y. After summing over y, the regular part of the adjoint filter equation then reads

(15) −∂F∂s(s,x)=λx1−(ℓ(s)2)(x+12)F(s,x+1)+μxF(s,x−1)−(λ+μ+ψ)xF(s,x),

which holds for x⩾ℓ(s) and $s\notin B\cup S_{0}\cup S_{1}\cup \{T\}$. The singular part is

(16) F(s,x)=2λx+1F∼(s,x+1),s∈B,ψ(x−ℓ(s))F∼(s,x),s∈S0,ψF∼(s,x),s∈S1,xn(1−ρ)x−nρn,s=T.

We note that F(s,x)=0 when x<ℓ(s). Let us make the ansatz

(17) $$F(s,x)=C(s)G(s{)}^{x-\tilde{\ell }(s)}\;H(s{)}^{\tilde{\ell }(s)}\;K(x,\tilde{\ell }(s)),$$

where G,H are smooth functions and C is càglàd and piecewise constant, with discontinuities only at genealogical events, so that

$$\underset{∼}{F}(s,x)=\underset{∼}{C}(s)G(s{)}^{x-\ell (s)}\;H(s{)}^{\ell (s)}\;K(x,\ell (s)).$$

Putting these into the first three cases of Eq. 16 yields conditions on C and K that are satisfied when

$$K(x,\ell )=\frac{x!}{(x-\ell )!}\;\text{and}\;\frac{C(s)}{\underset{∼}{C}(s)}=\left\{\begin{array}{ll} 2\lambda \;H(s), & s\in B,\\ \psi G(s)\;H(s{)}^{-1}, & s\in S_{0},\\ \psi , & s\in S_{1}. \end{array}\right.$$

To satisfy the last case of Eq. 16, we note that $\tilde{\ell }(T)=n$ and therefore impose the conditions

(18) $$G(T)=1-\rho ,\;H(T)=1,\;C(T)=\frac{\rho^{n}}{n!}.$$

Finally, upon substituting the ansatz into Eq. 15, we obtain for $s\notin B\cup S_{0}\cup S_{1}\cup \{T\}$,

$$-\frac{1}{F}\frac{\partial F}{\partial s}=-(x-\ell )\frac{G^{\prime }}{G}-\ell \frac{H^{\prime }}{H}\;=\lambda x\left(1-\frac{\left(\begin{array}{c} \ell \\ 2 \end{array}\right)}{\left(\begin{array}{c} x+1\\ 2 \end{array}\right)}\right)\frac{x+1}{x+1-\ell }G+\mu x\frac{x-\ell }{x}G^{-1}-(\lambda +\mu +\psi )x\;=\lambda (x+\ell )G+\mu (x-\ell )G^{-1}-(\lambda +\mu +\psi )\;x.$$

If this is to hold for all x and ℓ, we must have

(19) $$-\frac{G^{\prime }}{G}=\lambda G+\mu G^{-1}-(\lambda +\mu +\psi ),\;-\frac{H^{\prime }}{H}=2\lambda G-(\lambda +\mu +\psi ).$$

In passing, we note that Eqs. 19 are central to the original derivation of Stadler (2010). Eqs. 18 and 19 form a final-value problem that can be solved by elementary means to obtain closed-form expressions for G(s) and H(s). Corollary 6 then states that $ℒ=\int F(0,x)p_{0}(x)dx$. In particular, conditional on $X_{0}=x$, we have

(20) $$ℒ=\frac{x!}{(x-\ell (0))!}\frac{\rho^{n}}{n!}G(0{)}^{x-\ell (0)}\;H(0{)}^{\ell (0)}\;\psi^{\left|S_{1}\right|}\prod_{e\in B}(2\lambda H(e))\prod_{e\in S_{0}}\left(\psi G(e)H(e{)}^{-1}\right).$$

Stadler (2010, Thm. 3.5) derives an expression for this quantity in the special case x=ℓ(0)=1, which is equivalent up to the factor of n! explained in Remark 1.

The foregoing represents an independent verification of the correctness of the theory in the case of the simple linear birth-death process with constant sampling through time and one bulk-sampling event. It should be clear, however, that even within the context of the unstructured, linear, birth-death process, the theory presented here readily accommodates complexities such as time-varying sampling rates, multiple bulk-sampling events at specified or random times, and time-varying birth and death rates.

## 5. Discussion

The theory presented here represents a generalization of the existing coalescent and birth-death-process approaches to phylodynamic inference. Importantly, because it allows computation of the likelihood via strictly forward-in-time computations, it permits consideration of models for which time-reversal arguments are not available. Moreover, inasmuch as the formulae of Theorem 5 can be efficiently computed via sequential Monte Carlo, explicit expressions for transition probabilities are not needed: it is sufficient to be able to simulate from the population process. This feature of the algorithms—known as the *plug-and-play property* (He et al., 2010)—further expands the class of population models that can be confronted with data.

In particular, the theory gives us the freedom to choose models with many demes. For deterministic population models, Volz (2012) and Rasmussen et al. (2014) showed how one could accommodate discrete population structure. Their procedures involve solving a large number of differential equations backward in time, relying on the time-reversibility of deterministic dynamics. In general, this time-reversibility is not available for stochastic processes.

Some existing methods put rather severe limits on the form of the sampling model and, as Volz & Frost (2014) pointed out, misspecification of the sampling model can lead to large inferential biases. With the theory presented here, essentially arbitrary specification of the sampling model is possible. In particular, one can posit sampling at a rate which is an arbitrary function of time and state and include discrete sampling events as well. It is also possible to condition on the existence of samples.

If sequential Monte Carlo algorithms are used to compute the likelihoods of Theorem 5, then it is straightforward to simultaneously assimilate information from both time-series and genealogical data. One can therefore supplement traditional incidence, disease, or mortality time series with genealogical data to improve inference.

A limitation of the theory is that the population models are assumed to be pure jump processes, which allows consideration of demographic stochasticity and environmental stochasticity modeled by jumps involving multiple individuals (Bretó & Ionides, 2011), but disallows stochastic processes with a diffusive component. However, we anticipate that it will be possible to incorporate the full range of Markovian environmental stochasticity via extension of this theory to population models containing both diffusion and jump components.

Similarly, the theory presented here assumes that at most one birth event can occur at a time and that at most one deme can migrate or be sampled at a time (though multiple migrators or samples are permitted). These restrictions are not essential, and the proof of Theorem 1 can be adapted to accommodate relaxations of these assumptions. This will be developed in a sequel.

The price of the theory’s flexibility is primarily computational. When sequential Monte Carlo is used to evaluate the likelihood in Theorem 5, the computational effort scales linearly with the number of samples. In its most straightforward implementation—using an event-driven algorithm (e.g., Gillespie, 1977)—it scales nonlinearly with population size in general. However, stochastic simulation schemes are available that scale independently of population size (Higham, 2008). On the other hand, the importance sampling underlying Theorem 5 will in general require effort that is exponential in the number of demes. For models with many demes, therefore, approaches for ameliorating or circumventing this curse of dimensionality may be necessary. Critically, the substantial freedom one has in the choice of the importance-sampling kernel π can be exploited for this purpose. In particular, since it is permissible to “borrow information” from the future by means of the importance sampling, there is hope for highly efficient algorithmic computation.

## Figures and Tables

**Figure A1.**
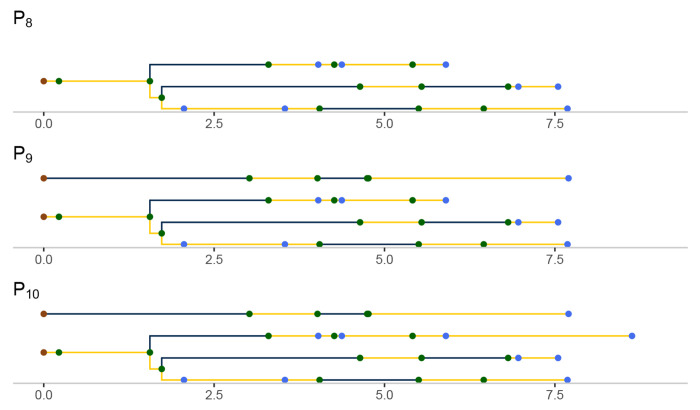
Embedded chain of the pruned genealogy process. Three successive states of the embedded chain of the pruned genealogy process are shown. At each step, a new lineage is added in a sampling event. This lineage extends backward until it either coalesces with the pre-existing genealogy or reaches t=0. Theorem 1 is derived by computing the probability that it coalesces at each possible point, conditional on the history of the population process.

**Figure C1.**
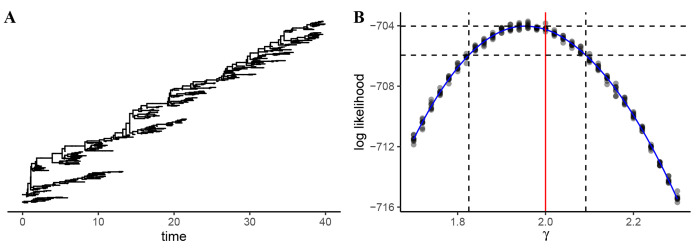
Likelihood computation for the SiRS model by sequential Monte Carlo. (A) A simulated genealogy for $\beta =4,\gamma =2,\omega =1,\psi =1,\left(S_{0},I_{0},R_{0}\right)=(97,3,0)$. (B) A slice through the likelihood surface at the true parameters in the γ-direction. Each point is a distinct Monte Carlo estimate. The blue curve is a LOESS smooth; the dashed lines bound the Monte Carlo-adjusted 95% confidence interval (Ionides et al., 2017).

**Figure C2.**
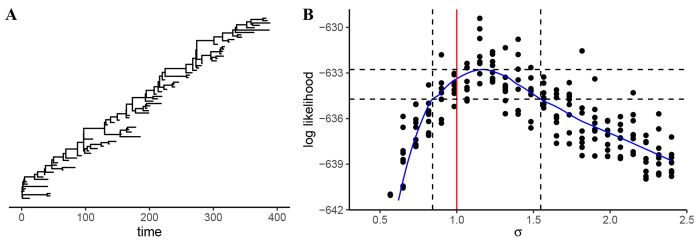
Likelihood computation for the SEIRS model by sequential Monte Carlo, using the scheme of Table C3. (A) Simulated genealogy for β=3,σ=1,γ=0.5,ψ=0.02, $\omega =0.08,\left(S_{0},E_{0},I_{0},R_{0}\right)=(70,1,0,50)$. (B) Likelihood slice in the σ-direction. Each point represents the estimate of an independent sequential Monte Carlo computation. The blue curve shows a LOESS smooth; the dashed vertical lines enclose the Monte Carlo-adjusted 95% confidence interval (Ionides et al., 2017).

## Appendix A. Proof of Theorem 1.

We are given a pruned genealogy P=(T,Z,Y) and a history H and wish to compute $Prob\left[P_{T}=P|H_{T}=H\right]$. First, note that if ev(H)⊉ev(P), then H and P are incompatible and $Prob\left[P_{T}=P|H_{T}=H\right]=0$. Similarly, if any event of H is incompatible with the local structure of P in the sense of §3.4.3, then $Prob\left[P_{T}=P|H_{T}=H\right]=0$. In either case, the conclusion of the theorem follows. Let us therefore suppose that neither of these conditions hold.

We can always construct a time-respecting, but otherwise arbitrary ordering of the sample nodes. Let $P_{j}$ represent the genealogy spanned by the first j samples in this ordering. Since each $P_{j}$ contains $P_{j^{\prime }}$ for all $j^{\prime }<j,P_{j}$ is a Markov chain. Therefore, we have the factorization

$$Prob[P|H]=\prod_{j}Prob\left[P_{j}|P_{j-1},H\right].$$

Each factor, $Prob\left[P_{j}|P_{j-1},H\right]$, can itself be factorized. In particular, suppose H and $P_{j-1}$ are given and suppose that it is the K-th event in H that introduces the j-th sample. The j-th lineage extends backward in time until it either coalesces with $P_{j-1}$ or reaches t=0 (Fig. A1). Moreover, since P is compatible with H, nothing can happen to this lineage between events of H. Therefore, we proceed backward, event by event, as follows. Since the type of event K is given, the color of the j-th lineage just prior to that event is known. At each subsequent event, the lineage either emerges from the event or does not. Moreover, if the lineage does emerge from the event, the color prior to the event is determined and the color remains unchanged if it does not. Suppose that lineage j is in deme i just to the right of event k<K and that the deme-i occupancy and production are $n_{i},r_{i}$, respectively. Let $\ell_{ij},s_{ij}$ be the lineage count and saturation at this event in $P_{j-1}$. Then the new lineage can be any one of the $n_{i}-\ell_{i}$ lineages as yet unaccounted for. Of these, $r_{i}-s_{ij}$ emerge from the event. Therefore, the probability that lineage j emerges from the event is $\left(r_{i}-s_{ij}\right)/\left(n_{i}-\ell_{ij}\right)$ and the probability that it does not is $\left(n_{i}-r_{i}-\ell_{ij}+s_{ij}\right)/\left(n_{i}-\ell_{ij}\right)$. Let $q_{jk}$ be the former if lineage j does in fact emerge from the event and the latter if it does not. Then $\mathrm{Prob}\left[P_{j}|P_{j-1},H\right]=\prod_{k}q_{jk}$ and $\mathrm{Prob}[P|H]=\prod_{jk}q_{jk}$.

Now fix k and consider the cumulative product $\prod_{m=1}^{j}q_{mk}$. Clearly $\ell_{1,k}=s_{1,k}=0$ and, if lineage j has not already coalesced to the right of k, then $\ell_{j+1,k}=\ell_{j,k}+1$. Moreover, if j emerges from k, then $s_{j+1,k}=s_{j,k}+1$ and $s_{j+1,k}=s_{j,k}$ if it does not. Therefore, as we accumulate each successive lineage j, we move one step to the right in the following diagram.

At each step, the lineage count increases by 1 and the saturation either increases by a unit or stays the same. The probability of each option is indicated on the corresponding arrow. Although there are multiple paths by which the process may arrive at the final lineage count and saturation, the product of the probabilities along each path is the same:

(ni−ℓiri−si)(niri).

Moreover, since each of the options is independent of the others,

$$\prod_{j}q_{jk}=\left(\begin{array}{ll} n & \ell \\ r & s \end{array}\right),$$

the latter being the binomial ratio defined in §3.5.

Alternatively, one can reason as follows. Conditional on $H_{T}=H$, at each time t∈ev(H), a jump of mark $U_{t}$ occurred, with a production of $r^{U_{t}}={\left(r_{i}\right)}_{i\in 𝔻}$, resulting in a deme-occupancy of $n\left(X_{t}\right)={\left(n_{i}\right)}_{i\in 𝔻}$. In P, at time t, there are $\ell_{i}=\ell_{i}\left(Y_{t}\right)$ lineages in deme i, of which $s_{i}=s_{i}\left({\tilde{Y}}_{t},Y_{t}\right)$ are emergent. By assumption, at each genealogical event, lineages within a deme are exchangeable: each has an identical probability of being involved. This exchangeability implies that each lineage present in a deme at time t was equally likely to have been one of the emergent lineages. In particular, at time t, the probability that $s_{i}$ of the $\ell_{i}$ deme-i lineages were among the $r_{i}$ of $n_{i}$ lineages emergent in the unpruned genealogy process is the same as the probability that, upon drawing $\ell_{i}$ balls without replacement from an urn containing $r_{i}$ red balls and $n_{i}-r_{i}$ black balls, exactly $s_{i}$ of the drawn balls are red, namely

ni−ℓiri−siℓisi(niri).

Because the lineages are labeled, each of the ℓisi equally probable sets of $s_{i}$ lineages is distinct; just one of these is the one present in P. Moreover, since, again conditional on $H_{T}=H$, the identities of the lineages involved in a genealogical event are random and independent of the identities selected at all other events, we have established that

$$Prob\left[P_{T}=P|H_{T}=H\right]=\prod_{t\in ev(H)}\left(\begin{array}{cc} n\left(X_{t}\right) & \ell \left(Y_{t}\right)\\ r^{U_{t}} & s\left({\tilde{Y}}_{t},Y_{t}\right) \end{array}\right).$$

Returning to the possibility that H is incompatible with P, since $Prob\left[P_{T}=P\right]=0$ if either any $Q_{U_{t}}=0$ or ev(P)⊈ev(H), we obtain the result. □

## Appendix B. Filter equations.

### B.1. Definition.

The likelihoods that appear in Theorems 2 and 5 are integrals over large sets of histories. As such, explicit expressions for them are not available, and we require mathematical tools to allow us to manipulate these quantities and devise algorithms for their numerical solution. The *filter equations* we introduce here are suitable for these purposes, and we devote this appendix to exposing their essential properties. This extremely convenient formalism has, to our knowledge, not been thoroughly exploited in the context we introduce here, though we note their resemblance to the constructions of Ogata (1978), Puri & Tuan (1986), Kliemann et al. (1990), and Giesecke & Schwenkler (2018).

**Definition.** Let $X_{t}$ be a continuous-time Markov process with KFE

(B1)
$$\frac{\partial u}{\partial t}(t,x)=\int u\left(t,x^{\prime }\right)\beta \left(t,x^{\prime },x\right)dx^{\prime }-\int u(t,x)\beta \left(t,x,x^{\prime }\right)dx^{\prime }.$$

Suppose that $B\;:\;{\mathbb{R}}_{+}\times {𝕏}^{2}\to {\mathbb{R}}_{+}$ and $\lambda \;:\;{\mathbb{R}}_{+}\times 𝕏\to \mathbb{R}$ are are given measurable functions. Let $S\subset {\mathbb{R}}_{+}$ be locally finite (i.e., S∩[0,t] is finite for all t>0). Then the system of equations

(B2)
$$\frac{\partial w}{\partial t}(t,x)=\int w\left(t,x^{\prime }\right)\beta \left(t,x^{\prime },x\right)B\left(t,x^{\prime },x\right)\text{d}x^{\prime }-\int w(t,x)\beta \left(t,x,x^{\prime }\right)\text{d}x^{\prime }-\lambda (t,x)w(t,x),\;\;t\notin S,$$

(B3)
$$\;w(t,x)=\int \tilde{w}\left(t,x^{\prime }\right)\beta \left(t,x^{\prime },x\right)B\left(t,x^{\prime },x\right)\text{d}x^{\prime },\;\;\;\;\;\;\;\;\;\;\;\;\;\;\;\;\;t\in S,$$

is called the *filter equation generated by* β, with *boost* B, *decay* λ, and *observed event times* S. The process $X_{t}$ is said to be the *driver* of the filter equation. Eq. B2 is the *regular part* of the filter equation; Eq. B3 is known as the *singular part*.

*Remark 2*. Trivially, a Kolmogorov forward equation is itself a filter equation with boost 1, decay 0, and S=∅.

### B.2. Properties.

The following results show how filter equations allow one to integrate over random histories. First, Lemma B1 shows how one integrates over the full space of histories using a regular filter equation. Lemma B2 builds on this when the set of histories is restricted.

**Lemma B1.** *Suppose that* $B:{\mathbb{R}}_{+}\times {𝕏}^{2}\to {\mathbb{R}}_{+}$ *is measurable. Let* $V_{t}$ *be an* ${\mathbb{R}}_{+}$-*valued random process satisfying*

$$𝔼\left[V_{t}|H_{t}=H_{t}\right]=\prod_{e\in ev\left(H_{t}\right)}B\left(e,{\tilde{X}}_{e},X_{e}\right).$$

Let the family of measures $\lambda_{t}$ on 𝕏 be defined by

$$\lambda_{t}(ℰ)=𝔼\left[V_{t}\cdot 𝟙\left\{X_{t}\in ℰ\right\}\right],$$

*for measurable* ℰ, *and let* w(t,x) *be the density of* $\lambda_{t}$, *i.e.*, $\lambda_{t}(dx)=w(t,x)dx$. *In particular,* $𝔼\left[V_{t}\right]=\lambda_{t}(𝕏)=\int w(t,x)dx$. *Then* w *satisfies the initial condition* $w(0,x)=p_{0}(x)$ *and the regular filter equation,*

(B4)
$$\frac{\partial w}{\partial t}=\int w\left(t,x^{\prime }\right)\alpha \left(t,x^{\prime },x\right)B\left(t,x^{\prime },x\right)dx^{\prime }-\int w(t,x)\alpha \left(t,x,x^{\prime }\right)dx^{\prime }.$$

*Proof*. Since $Prob\left[V_{0}=1\right]=1,\lambda_{0}(ℰ)=Prob\left[X_{0}\in ℰ\right]$, which implies that $w(0,x)=p_{0}(x)$. For t>0 and Δ>0, the expectation can be broken into three terms, according to whether $H_{t}$ has zero, one, or more than one event in (t,t+Δ]. Accordingly, as Δ↓0,

$$w(t+\Delta ,x)=\left(1-\Delta \int \alpha \left(t,x,x^{\prime }\right)dx^{\prime }\right)w(t,x)+\Delta \int \alpha \left(t,x^{\prime },x\right)\;B\left(t,x^{\prime },x\right)w\left(t,x^{\prime }\right)dx^{\prime }+o(\Delta ).$$

Forming a difference quotient and taking the limit, we obtain Eq. B4. Note that here we use the assumption that α and B are càdlàg as functions of t. □

When events are known to have occurred at particular times, it is of interest to integrate over those histories that include an event at each of these times. This leads to singular filter equations, as the next lemma shows. Before we state the lemma, some terminology is needed. Let 𝕊 be the space of increasing, locally finite sequences in ${\mathbb{R}}_{+}$, with the topology induced by the Skorokhod metric and Lebesgue measure. For $t\in {\mathbb{R}}_{+}$ and S∈𝕊, let $S_{t}≔S\cap [0,t]$. Thus if S∈𝕊 and $S_{t}=\left({\overset{ˆ}{s}}_{1},\ldots ,{\overset{ˆ}{s}}_{K}\right)$, then the infinitesimal element of Lebesgue measure at $S_{t}$ is $dS_{t}=\prod_{n=1}^{K}d{\overset{ˆ}{s}}_{n}$.

**Lemma B2.** *Suppose that* $B:{\mathbb{R}}_{+}\times {𝕏}^{2}\to {\mathbb{R}}_{+}$ *is measurable and* $V_{t}$ *is an* ${\mathbb{R}}_{+}$-*valued random process satisfying*

$$𝔼\left[V_{t}|H_{t}=H_{t}\right]=\prod_{e\in ev\left(H_{t}\right)}B\left(e,{\tilde{X}}_{e},X_{e}\right).$$

*Let* $\lambda_{t}$ *be a family of measures on* 𝕏×𝕊 *defined by*

$$\lambda_{t}(ℰ,S)=𝔼\left[V_{t}\cdot 𝟙\left\{X_{t}\in ℰ\right\}\cdot 𝟙\left\{\exists S\in 𝒮\;\text{s.t.}\;ev\left(H_{t}\right)⊇S_{t}\right\}\right],$$

*whenever* ℰ⊆𝕏 *and* 𝒮⊆𝕊 *are measurable. Let* w(t,x,S) *be the density of this measure, i.e.,*

$$\lambda_{t}(dxdS)=w(t,x,S)dxdS_{t}.$$

Then w satisfies

(B5)
$$\frac{\partial w}{\partial t}(t,x,S)=\int w\left(t,x^{\prime },S\right)\;\alpha \left(t,x^{\prime },x\right)\;B\left(t,x^{\prime },x\right)\text{d}x^{\prime }-\int w(t,x,S)\alpha \left(t,x,x^{\prime }\right)\text{d}x^{\prime },\;\;t\notin S,$$

(B6)
$$w(t,x,S)=\int \tilde{w}\left(t,x^{\prime },S\right)\alpha \left(t,x^{\prime },x\right)\;B\left(t,x^{\prime },x\right)\text{d}x^{\prime },\;\;\;\;\;\;\;\;\;\;\;\;\;t\in S.$$

*Proof*. The proof proceeds by induction on the cardinality of $S_{t}$. The base case, for which $S_{t}=\emptyset$, follows immediately from Lemma B1. Assuming that it holds for $\left|S_{t}\right|<K$, one has only to verify Eq. B6. This can be accomplished by integrating Eq. 6 directly. □

*Remark* 3. In the same way that Eqs. 4 and 5 can be represented as a single equation by means of a Dirac delta notation, the Eqs. B5 and B6 can be collapsed into a more compact form if β is allowed to have atoms at a countable set of time-points and the boost B is adjusted appropriately.

### B.3. Adjoint filter equation.

Given driver, boost, and decay functions, a filter equation determines a family of measures on 𝕏. Specifically, suppose that, for s⩽t and S⊂ℝ locally finite, $w\left(s,x,t,x^{\prime }\right)$ satisfies the filter equation

(B7)
$$w\left(s,x,s,x^{\prime }\right)=\delta \left(x,x^{\prime }\right)$$

(B8)
$$\frac{\partial w}{\partial t}\left(s,x,t,x^{\prime }\right)=\int w(s,x,t,\xi )\;\beta \left(t,\xi ,x^{\prime }\right)\;B\left(t,\xi ,x^{\prime }\right)\text{d}\xi -\int w\left(s,x,t,x^{\prime }\right)\;\beta \left(t,x^{\prime },\xi \right)\text{d}\xi -\lambda \left(t,x^{\prime }\right)w\left(s,x,t,x^{\prime }\right),\;\;\;t\notin S,$$

(B9)
$$w\left(s,x,t,x^{\prime }\right)=\int \tilde{w}(s,x,t,\xi )\;\beta \left(t,\xi ,x^{\prime }\right)\;B\left(t,\xi ,x^{\prime }\right)\text{d}\xi ,$$

Let ω be the measure whose density is *w*:

$$\omega (s,x,t,ℰ)≔\int_{ℰ}w\left(s,x,t,x^{\prime }\right)dx^{\prime }.$$

If f:𝕏→ℝ is a measurable function, then one defines its integral with respect to this measure in the usual way:

$$\omega (s,x,t,f)=\int \omega \left(s,x,t,dx^{\prime }\right)f\left(x^{\prime }\right)=\int w\left(s,x,t,x^{\prime }\right)f\left(x^{\prime }\right)dx^{\prime }.$$

In particular, ω(s,x,t,f) is itself a measurable function of x and we have the Chapman-Kolmogorov-like relation

(B10)
ω(s,x,t,f)=∫ω(s,x,τ,ω(τ,ξ,t,f))dξ=∫ω(s,x,τ,dξ)ω(τ,ξ,t,f),

whenever s⩽τ⩽t. Let us now fix t and f and write F(s,x)=ω(s,x,t,f). Let us take β,B, and λ to be càdlàg in t and F to be càglàd in s. Applying Eq. B10 we obtain, for Δ>0 and s∉S,

$$F(s-\Delta ,x)=\left(1-\Delta \int \beta (s-\Delta ,x,\xi )d\xi -\Delta \lambda (s-\Delta ,x)\right)\;F(s,x)+\Delta \int \beta (s-\Delta ,x,\xi )B(s-\Delta ,x,\xi )F(s,\xi )d\xi +o(\Delta ).$$

Taking Δ↓0 gives

(B11)
$$-\frac{\partial F}{\partial s}=\int \tilde{\beta }(s,x,\xi )[\tilde{B}(s,x,\xi )F(s,\xi )-F(s,x)]\;d\xi -\tilde{\lambda }(s,x)F(s,x).$$

For s∈S, we have

(B12)
$$F(s,x)=\int \omega (s,x,s,d\xi )\underset{∼}{F}(s,\xi )=\int \beta (s,x,\xi )B(s,x,\xi )\underset{∼}{F}(s,\xi )d\xi ,$$

where $\underset{∼}{F}$ denotes the right-limit of F. Eqs. B11 and B12 constitute the *adjoint form* of Eqs. B7–B9. Together with the final condition F(t,x)=f(x), these determine ω(s,x,t,f) for all s⩽t. Note that, when B=1 and λ=0, the adjoint filter equation becomes the familiar Kolmogorov backward equation (Eq. 3) for the driving process.

### B.4. Solving filter equations by sequential Monte Carlo.

Filter equations afford a convenient means of computing expectations and likelihoods for pure jump processes. This is facilitated by the following Lemma, the statement of which uses a one-sided Dirac delta function. Specifically, let $\delta \left(v,v^{\prime }\right)$ be the right-sided Dirac delta function satisfying $\delta \left(v,v^{\prime }\right)=0$ for $v\neq v^{\prime }$ and

$$\int_{a}^{b}f(v)\delta \left(v,v^{\prime }\right)dv=f\left(v^{\prime }\right)𝟙\left\{v^{\prime }\in [a,b)\right\},$$

whenever f is càdlàg and −∞⩽a<b⩽∞.

**Lemma B3.** Eqs. B2 *and* B3 *are satisfied by* $w(t,x)=\int_{0}^{\infty }vu(t,x,v)dv$, *where* u(t,x,v) *satisfies the KFE*

(B13)
$$\begin{array}{ll} \frac{\partial u}{\partial t}(t,x,v)=\frac{\partial }{\partial v}[\lambda (t,x)vu(t,x,v)]+\int_{0}^{\infty }\int u\left(t,x^{\prime },v^{\prime }\right)\beta \left(t,x^{\prime },x\right)\delta \left(v,B\left(t,x^{\prime },x\right)v^{\prime }\right)\text{d}x^{\prime }\text{d}v^{\prime }-\int_{0}^{\infty }\int u(t,x,v)\beta \left(t,x,x^{\prime }\right)\delta \left(v^{\prime },B\left(t,x,x^{\prime }\right)v\right)\text{d}x^{\prime }\text{d}v^{\prime }, & t\notin S,\\ u(t,x,v)\text{d}x=\int_{0}^{\infty }\tilde{u}\left(t,x^{\prime },v^{\prime }\right)\pi \left(t,x^{\prime },\text{d}x\right)\delta \left(v,A\left(t,x^{\prime }\right)B\left(t,x^{\prime },x\right)v^{\prime }\right)\text{d}x^{\prime }\text{d}v^{\prime }, & t\in S. \end{array}$$

Here, $A(t,x)≔\int \beta \left(t,x,x^{\prime }\right)dx^{\prime }$ *and* $\pi \left(t,x,dx^{\prime }\right)≔\beta \left(t,x,x^{\prime }\right)dx^{\prime }/A(t,x)$.

*Proof*. For each t∉S, we have

$$\frac{\partial w}{\partial t}(t,x)=\int_{0}^{\infty }v\frac{\partial u}{\partial t}(t,x,v)\text{d}v\;=\int_{0}^{\infty }\iint_{0}^{\infty }vu\left(t,x^{\prime },v^{\prime }\right)\beta \left(t,x^{\prime },x\right)\delta \left(v,B\left(t,x^{\prime },x\right)v^{\prime }\right)\text{d}v\text{d}x^{\prime }\text{d}v^{\prime }\;-\int_{0}^{\infty }\iint_{0}^{\infty }vu(t,x,v)\beta \left(t,x,x^{\prime }\right)\delta \left(v^{\prime },B\left(t,x,x^{\prime }\right)v\right)\text{d}v\text{d}x^{\prime }\text{d}v^{\prime }\;+\int_{0}^{\infty }v\frac{\partial }{\partial v}[\lambda (t,x)vu(t,x,v)]\text{d}v.$$

Here, the non-explosivity assumption guarantees that we can differentiate under the integral sign and exchange the order of integration. Moreover, it ensures that u→0 as v→∞. Hence, by evaluating the first integral with respect to v, the second with respect to $v^{\prime }$, and the third by parts, we obtain

$$\frac{\partial w}{\partial t}(t,x)=\int v^{\prime }u\left(t,x^{\prime },v^{\prime }\right)\beta \left(t,x^{\prime },x\right)B\left(t,x^{\prime },x\right)dv^{\prime }dx^{\prime }-\int vu(t,x,v)\beta \left(t,x,x^{\prime }\right)dvdx^{\prime }-\lambda (t,x)\int vu(t,x,v)dv,$$

which is simplified to obtain Eq. B2. Similarly, at each t∈S, we have

$$w(t,x)\text{d}x=\int_{0}^{\infty }\iint_{0}^{\infty }v\tilde{u}\left(t,x^{\prime },v^{\prime }\right)\pi \left(t,x^{\prime },\text{d}x\right)\delta \left(v,A\left(t,x^{\prime }\right)B\left(t,x^{\prime },x\right)v^{\prime }\right)\text{d}x^{\prime }\text{d}v^{\prime }\text{d}v\;=\int_{0}^{\infty }\int v^{\prime }\tilde{u}\left(t,x^{\prime },v^{\prime }\right)\pi \left(t,x^{\prime },\text{d}x\right)A\left(t,x^{\prime }\right)B\left(t,x^{\prime },x\right)\text{d}x^{\prime }\text{d}v^{\prime }\;=\int \tilde{w}\left(t,x^{\prime }\right)\beta \left(t,x^{\prime },x\right)B\left(t,x^{\prime },x\right)\text{d}x^{\prime }\text{d}x.$$

which is equivalent to Eq. B3 □

Eqs. B13 are recognizable as the KFE of a certain process ($X_{t},V_{t}$). In particular, the driver $X_{t}$ has KFE Eq. B1. $V_{t}$ *is directed* by $X_{t}$ in the sense that V has jumps wherever X does: when X jumps at time t from x to $x^{\prime },V$ jumps by the multiplicative factor $B\left(t,x,x^{\prime }\right)⩾0$. Between jumps, $V_{t}$ decays deterministically and exponentially at rate λ(t,x). At the known times in S,X jumps according to the probability kernel π and, V jumps by the factor $A(t,x)B\left(t,x,x^{\prime }\right)$. If we view $V_{t}$ as a weight, then Lemma B3 tells us how the $V_{t}$-weighted average of $X_{t}$ evolves in time: this average is simply ∫w(t,x)dx. Thus, Lemma B3 gives a recipe for the integration of Eqs. B5 and B6 in the Monte Carlo sense. A pseudocode representation of this procedure is given in Algorithm B1. This is a simple sequential importance sampling algorithm and as such can be improved upon in many ways (Doucet et al., 2001; Arulampalam et al., 2002; Wills & Schön, 2023). Our purpose here is merely to illustrate the implications of Lemma B3 by showing how the features of Eqs. B2 and B3 translate into computations, in the simplest possible context.

## Appendix C. Examples.

### C.1. SIRS model.

King et al. (2022) worked out formulae for the exact likelihood of a genealogy induced by an SIRS model. The theory developed in this paper applies, but since there is only one deme in this model, this is a simple case. Its state vector is x=(S,I,R) and its KFE is

$$\frac{\partial v}{\partial t}(S,I,R)=\frac{\beta (S+1)(I-1)}{N}v(t,S+1,I-1,R)-\frac{\beta SI}{N}v(t,S,I,R)\;\;+\gamma (I+1)v(t,S,I+1,R-1)-\gamma Iv(t,S,I,R)\;\;+\omega (R+1)v(t,S-1,I,R+1)-\omega Rv(t,S,I,R).$$

Here N=S+I+R is the host population size. Note that, though the theory allows for time-dependent event rates, this example is time-homogeneous. This model has one deme and occupancy function n(x)=I. There are four kinds of jumps: transmission, recovery, waning of immunity, and sampling. Accordingly, the marks are 𝕌={Trans, Recov, Wane, Sample}. Table C1 gives $\alpha_{u},r^{u}$, and the event type for each of these marks.

**Table C1.** Elements of the SIRS model pertinent to the genealogy process. The table shows the rate ($\alpha_{u}$), jump ($x\mapsto x^{\prime }$), production ($r^{u}$), and event type for each of the model’s four marks (u).

u	$\alpha_{u}$	$x\mapsto x^{'}$	$r^{u}$	Event type
Trans	$\frac{\beta SI}{N}$	(S,I)↦(S−1,I+1)	2	pure birth
Recov	γI	(I,R)↦(I−1,R+1)	0	pure death
Wane	ωR	(S,R)↦(S+1,R−1)	0	neutral
Sample	ψI	x↦x	1	pure sample

Given an obscured genealogy Z, let $ev(Z)=B\cup S_{0}\cup S_{1}$, where B is the set of branch-times, and $S_{0},S_{1}$ are the sets of sample-times with saturations 0 and 1, respectively. Since there is only one deme, paintings of Z can differ only in number and position of inline, internal nodes along branches. Each of these can only correspond to

**Algorithm B1:** Sequential Monte Carlo filter.

The following is a simple Monte Carlo scheme for the filter given by Eqs. B2 and B3. It takes as input an initial time $t_{0}$, a final time T, and a sequence of observed event times $S=\left\{t_{1},\ldots ,t_{K}\right\}$. We order the latter so that $t_{0}⩽t_{1}<t_{2}<\cdots <t_{K}⩽t_{K+1}≔T$. In addition, we are given event rates β, a boost function B, a decay function λ, and the initial distribution of latent states $p_{0}$. As in Lemma B3, we let $$A(t,x)≔\int \beta \left(t,x,x^{\prime }\right)dx^{\prime },\;\;\pi \left(t,x,dx^{\prime }\right)≔\frac{\beta \left(t,x,x^{\prime }\right)}{A(t,x)}dx^{\prime }.$$ For a given, fixed ensemble size, $J\in {\mathbb{Z}}_{+}$, the algorithm returns an unbiased Monte Carlo estimate, $\overset{ˆ}{ℒ}$, of the likelihood.	

1: **procedure** SMCFilter$\left(t_{0},T,S,\beta ,B,\lambda ,p_{0},J\right)$ Initialize an ensemble of latent states and weights:	
2: $x_{j}∼p_{0}(\cdot )$, for *j* = 1, …, *J*	
3: *w_j_* ↑ 1, for *j* = 1, …, *J*	
4: **for** *k* = 0, …, *K* **do**	▷ loop over observed event times
5: **for** *j* = 1, … , *J* **do**	▷ loop over the ensemble
6: *t* ← *t_k_*	
7: **if** *k* > 0 **then**	▷ singular part (Eq. B3)
8: $x_{j}^{\prime }∼\pi \left(t,x_{j},\cdot \right)$	
9: $w_{j}\leftarrow w_{j}A\left(t,x_{j}\right)B\left(t,x_{j},x_{j}^{\prime }\right)$	
10: $x_{j}\leftarrow x_{j}^{\prime }$	
11: **while** *t* < *t*_*k*+1_ **do**	▷ regular part (Eq. B2)
12: $\text{Δ}∼\mathrm{Exp}\left(A\left(t,x_{j}\right)\right)$	▷ Sojourn times are exponentially distributed.
13: **if** *t* + Δ < *t*_*k*+1_ **then**	
14: $x_{j}^{\prime }∼\pi \left(t,x_{j},\cdot \right)$	
15: $t^{\prime }\leftarrow t+\text{Δ}$	
16: $w_{j}\leftarrow w_{j}B\left(t^{\prime },x_{j},x_{j}^{\prime }\right)\exp\left(-\lambda \left(t,x_{j}\right)\text{Δ}\right)$	
17: $x_{j}\leftarrow x_{j}^{\prime }$	
18: $t\leftarrow t^{\prime }$	
19: **else**	
20: Δ ← *t*_*k*+1_ – *t*	
21: $w_{j}\leftarrow w_{j}\exp\left(-\lambda \left(t,x_{j}\right)\text{Δ}\right)$	
22: $t\leftarrow t_{k+1}$	
23: $\hat{ℒ}\leftarrow \frac{1}{J}\underset{j}{\sum }w_{j}$	

u=Trans with s=1. For t∉ev(Z), we can take the importance sampling distribution to be

$$\pi_{u}=\left\{\begin{array}{ll} c, & u=\text{Trans},\;s=0,t\notin ev(Z),\\ \frac{1-c}{\ell }, & u=\text{Trans},\;s=1,t\notin ev(Z),\\ 0, & \text{otherwise.} \end{array}\right.$$

Here c is an arbitrary probability that does not affect the computation. The relevant binomial ratios are

$$\left(\begin{array}{ll} n & \ell \\ r & s \end{array}\right)=\left\{\begin{array}{ll} \frac{\left(I-\ell )(I-\ell -1\right)}{I(I-1)}, & u=\text{Trans,}\;s=0,\\ \frac{2(I-\ell )}{I(I-1)}, & u=\text{Trans,}\;s=1,\\ \frac{2}{I(I-1)}, & u=\text{Trans,}\;s=2,\\ \frac{I-\ell }{I}, & u=\text{Sample,}\;s=0\\ \frac{1}{I}, & u=\text{Sample,}\;s=1. \end{array}\right.$$

This leads, for t∉ev(Z), to the following regular part of the filter equation:

$$\frac{\partial w}{\partial t}=\frac{\beta (S+1)(I-1)}{N}\left(1-\frac{\left(\begin{array}{c} \ell (t)\\ 2 \end{array}\right)}{\left(\begin{array}{c} I\\ 2 \end{array}\right)}\right)w(t,S+1,I-1,R)-\frac{\beta SI}{N}w(t,S,I,R)\;+\gamma (I+1)w(t,S,I+1,R-1)-\gamma Iw(t,S,I,R)\;+\omega (R+1)w(t,S-1,I,R+1)-\omega Rw(t,S,I,R)\;-\psi Iw(t,S,I,R).$$

Here, we have summed over the various paintings for u= Trans, s<2. Note the presence of the decay term proportional to ψ. At event-times t∈ev(Z), the singular part of the filter equation reads

$$w(t,S,I,R)=\left\{\begin{array}{ll} \tilde{w}(t,S+1,I-1,R)\frac{2\beta (S+1)}{NI}, & t\in B,\\ \tilde{w}(t,S,I,R)\psi (I-\ell (t)), & t\in S_{0},\\ \tilde{w}(t,S,I,R)\psi , & t\in S_{1}. \end{array}\right.$$

Finally, note that w(t,S,I,R)=0 for all I<ℓ(t).

### C.2. SEIRS model.

A simple, yet interesting, model with more than one deme is the SEIRS model (Fig. 1A). The state space is ${\mathbb{R}}_{+}^{4}$, with the state x=(S,E,I,R) defined by the numbers of hosts in each of the four compartments. The KFE for the population process is

$$\frac{\partial v}{\partial t}(t,S,E,I,R)=\frac{\beta (S+1)I}{N}v(t,S+1,E-1,I,R)-\frac{\beta SI}{N}v(t,S,E,I,R)\;+\sigma (E+1)v(t,S,E+1,I-1,R)-\sigma Ev(t,S,E,I,R)\;+\gamma (I+1)v(t,S,E,I+1,R-1)-\gamma Iv(t,S,E,I,R)\;+\omega (R+1)v(t,S-1,E,I,R+1)-\omega Rv(t,S,E,I,R),$$

where N=S+E+I+R is the total population size. Note that the terms associated with sampling cancel each other in the KFE, since, with the state space defined as above, sampling has no effect on the state.

This model has two demes: 𝔻={E,I}. Its deme occupancy function is n(x)=(E,I). There are five kinds of jumps: transmission, progression, recovery, waning of immunity, and sampling. The corresponding marks are 𝕌={Trans, Prog, Recov, Wane, Sample}. Table C2 gives $\alpha_{u},r^{u}$, and the event type for each of these marks.

**Table C2.** Elements of the SEIRS model pertinent to the genealogy process. The table shows the rate $\alpha_{u}$, jump $x\mapsto x^{\prime }$ production $r^{u}$ and event type for each of the model’s five marks (u).

u	$\alpha_{u}$	$x\mapsto x^{'}$	$r^{u}$	Event type
Trans	$\frac{\beta SI}{N}$	(S,E)↦(S−1,E+1)	(1, 1)	pure birth
Prog	σE	(E,I)↦(E−1,I+1)	(0,1)	pure migration
Recov	γI	(I,R)↦(I−1,R+1)	(0,0)	pure death
Wane	ωR	(S,R)↦(S+1,R−1)	(0,0)	neutral
Sample	ψI	x↦x	(0,1)	pure sample

**Table C3.** Elements of a scheme for numerically computing the likelihood under the SEIRS model. The regular portion of the filter equation holds in between genealogical events (i.e., for $t\notin B\cup S_{0}\cup S_{1}$ the singular portion describes the effect of these events $t\in B\cup S_{0}\cup S_{1}$ Each line corresponds to a potential event. An event with mark u and saturation s has the boost given by the binomial ratio shown (third column). The $y\mapsto y^{\prime }$ column depicts the proposed painting schematically, and π is the probability of that proposal. Blue is used for the E deme and yellow for the I deme. For each line, the filter equation contains m terms. An asterisk(*) stands for cases not explicitly mentioned.

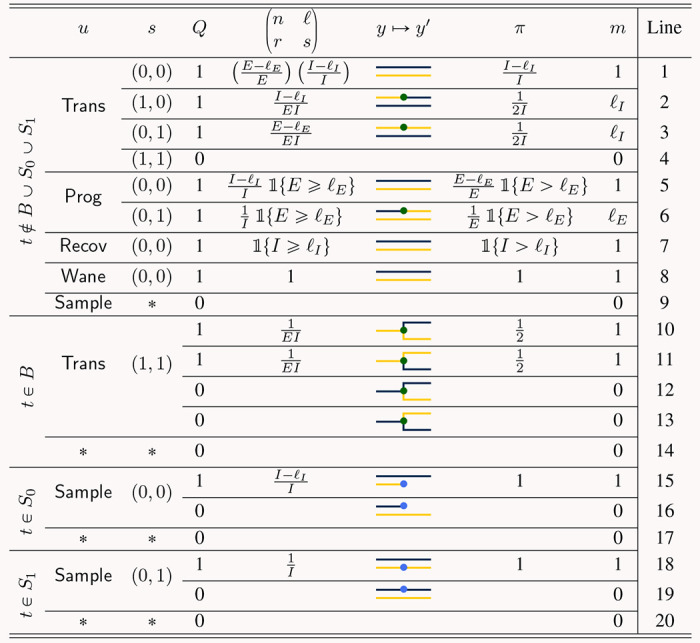

In the application of Theorem 5 and Corollary 6, one has wide latitude in the selection of the kernels π and q. Table C3 gives a particular set of choices that are *naïve* in the sense that they refrain from borrowing information from the future. Such a naïve filter is always available, though it will rarely be optimal.

Using the choices of Table C3, we proceed to write down a filter equation for the SEIRS model. As previously, given an obscured genealogy, let B be the set of its branch times, $S_{0}$ be the set of tip-sample times, and $S_{1}$ be the set of inline sample times. Then for $t\notin B\cup S_{0}\cup S_{1}$, the filter equation reads:

(C1)
$$\frac{\partial w}{\partial t}(t,S,E,I,R,y)=\frac{\beta (S+1)I}{N}\left(1-\frac{\ell_{E}}{E}\right)\;\left(1-\frac{\ell_{I}}{I}\right)w(t,S+1,E-1,I,R,y)\;+\overset{\ell_{I}}{\underset{k=1}{\sum }}\frac{\beta (S+1)I}{N}\frac{1}{E}\left(1-\frac{\ell_{I}}{I}\right)\;w\left(t,S+1,E-1,I,R,{\tilde{y}}_{2}^{k}\right)\;+\overset{\ell_{I}}{\underset{k=1}{\sum }}\frac{\beta (S+1)I}{N}\frac{1}{I}\left(1-\frac{\ell_{E}}{E}\right)\;w\left(t,S+1,E-1,I,R,{\tilde{y}}_{3}^{k}\right)-\frac{\beta SI}{N}w(t,S,E,I,R,y)\;+\sigma (E+𝟙)\left\{E⩾\ell_{E}\right\}\;\left(1-\frac{\ell_{I}}{I}\right)\;w(t,S,E+1,I-1,R,y)\;+\overset{\ell_{E}}{\underset{k=1}{\sum }}\sigma (E+𝟙)\left\{E⩾\ell_{E}\right\}\frac{1}{I}w\left(t,S,E+1,I-1,R,{\tilde{y}}_{6}^{k}\right)-\sigma Ew(t,S,E,I,R,y)\;+\gamma (I+𝟙)\left\{I⩾\ell_{I}\right\}w(t,S,E,I+1,R-1,y)-\gamma Iw(t,S,E,I,R,y)\;+\omega (R+1)w(t,S-1,E,I,R+1,y)-\omega Rw(t,S,E,I,R,y)-\psi Iw(t,S,E,I,R,y).$$

Here, ${\tilde{y}}_{j}^{k}$ refers to the coloring of the tree immediately preceding the proposal indicated on line j of Table C3. The integer k specifies the particular branch on which the change occurs.

The singular portion of the filter equation has one component for each distinct type of genealogical event:

(C2)
$$w(t,S,E,I,R,y)=\left\{\begin{array}{ll} \frac{\beta (S+1)I}{N}\frac{1}{EI}\tilde{w}\left(t,S+1,E-1,I,R,{\tilde{y}}_{10}\right) & \\ \;+\frac{\beta (S+1)I}{N}\frac{1}{EI}\tilde{w}\left(t,S+1,E-1,I,R,{\tilde{y}}_{11}\right), & t\in B,\\ \psi \left(I-\ell_{I}\right)\tilde{w}\left(t,S,E,I,R,{\tilde{y}}_{15}\right), & t\in S_{0},\\ \psi \tilde{w}\left(t,S,E,I,R,{\tilde{y}}_{18}\right), & t\in S_{1}. \end{array}\right.$$

In addition to Eqs. C1 and C2, the quantity w should satisfy the condition w(t,S,E,I,R,y)=0 whenever $E<\ell_{E}$ or $I<\ell_{I}$.

A variety of importance-sampling kernels q,π are permissible under the terms of Theorem 5. With a particular choice of importance-sampling kernel, the filter equation uniquely specifies a sequential Monte Carlo algorithm for estimating the likelihood. The specific choices made in Table C3 underlie the results displayed in Fig. C2. Some numerical results obtained using this scheme are presented in Fig. C2.
