# Supplementary material for: EXACT PHYLODYNAMIC LIKELIHOOD VIA STRUCTURED MARKOV GENEALOGY PROCESSES
Source: ArXiv. 2025 Jan 16:arXiv:2405.17032v2. Originally published 2024 May 27. Preprint. [Version 2] (PMC11160859)
Supplement: Supplementary file 1 [file NIHPP2405.17032v2-supplement-1.pdf]

Appendix A. Filter equations.

The likelihoods that appear in Theorems 2 and 4 are integrals over large sets of histories. As such, explicit expressions for them are not available, and we require mathematical tools to allow us to manipulate these quantities and devise algorithms for their numerical solution. The *filter equations* we introduce here are suitable for these purposes, and we devote this appendix to exposing their essential properties. This extremely convenient formalism has, to our knowledge, not been thoroughly exploited, though we note their resemblance to the constructions of Ogata (1978), Puri & Tuan (1986), Kliemann et al. (1990), and Giesecke & Schwenkler (2018).

Definition. Let $\mathbf{X}_t$ be a continuous-time Markov process with KFE

$$\frac{\partial u}{\partial t}(t,x) = \int u(t,x')\,\beta(t,x',x)\,\mathrm{d}x' - \int u(t,x)\,\beta(t,x,x')\,\mathrm{d}x'. \tag{A1}$$

Suppose that $B : \mathbb{R}_+ \times \mathbb{X}^2 \to \mathbb{R}_+$ and $\lambda : \mathbb{R}_+ \times \mathbb{X} \to \mathbb{R}$ are are given measurable functions. Let $S \subset \mathbb{R}_+$ be countable and locally finite (i.e., $S \cap [0,t]$ is finite for all $t > 0$). Then the system of equations

$$\frac{\partial w}{\partial t}(t,x) = \int w(t,x')\,\beta(t,x',x)\,B(t,x',x)\,\mathrm{d}x' - \int w(t,x)\,\beta(t,x,x')\,\mathrm{d}x' - \lambda(t,x)\,w(t,x), \qquad t \notin S, \tag{A2}$$

$$w(t,x) = \int \tilde{w}(t,x')\,\beta(t,x',x)\,B(t,x',x)\,\mathrm{d}x', \qquad t \in S, \tag{A3}$$

is called the *filter equation generated by* β, with *boost* B, *decay* λ, and *observation times* S. The process $\mathbf{X}_t$ is said to be the *driver* of the filter equation. Eq. A2 is the *regular part* of the filter equation; Eq. A3 is known as the *singular part*.

Remark. Trivially, a Kolmogorov forward equation is itself a filter equation with boost 1, decay 0, and $S = \varnothing$.

The following results show how filter equations allow one to integrate over random histories. First, Lemma A1 shows how one integrates over the full space of histories using a regular filter equation. Lemma A2 builds on this when the set of histories is restricted.

Lemma A1. *Suppose that* $B : \mathbb{R}_+ \times \mathbb{X}^2 \to \mathbb{R}_+$ *is measurable. Let* $\mathbf{V}_t$ *be an* $\mathbb{R}_+$*-valued random process satisfying*

$$\mathbb{E}\left[\mathbf{V}_t \mid \mathbf{H}_t = \mathrm{H}_t\right] = \prod_{e \,\in\, \mathsf{ev}(\mathrm{H}_t)} B(e, \tilde{\mathrm{X}}_e, \mathrm{X}_e).$$

Let the family of measures λ_t *on* $\mathbb{X}$ *be defined by*

$$\lambda_t(\mathcal{E}) = \mathbb{E}\left[\mathbf{V}_t \cdot \mathbb{1}\{\mathbf{X}_t \in \mathcal{E}\}\right],$$

for measurable $\mathcal{E}$, and let $w(t,x)$ be the density of λ_t, i.e., $\lambda_t(\mathrm{d}x) = w(t,x)\,\mathrm{d}x$. In particular, $\mathbb{E}\left[\mathbf{V}_t\right] = \lambda_t(\mathbb{X}) = \int w(t,x)\,\mathrm{d}x$. Then w satisfies the initial condition $w(0,x) = p_0(x)$ and the regular filter equation,

$$\frac{\partial w}{\partial t} = \int w(t,x')\,\alpha(t,x',x)\,B(t,x',x)\,\mathrm{d}x' - \int w(t,x)\,\alpha(t,x,x')\,\mathrm{d}x'. \tag{A4}$$

Proof. Since $\mathsf{Prob}\left[\mathbf{V}_0 = 1\right] = 1$, $\lambda_0(\mathcal{E}) = \mathsf{Prob}\left[\mathbf{X}_0 \in \mathcal{E}\right]$, which implies that $w(0,x) = p_0(x)$. For $t > 0$ and $\Delta > 0$ sufficiently small, the expectation can be broken into three terms, according to whether H_t has zero, one, or more than one event in $(t-\Delta, t]$. Accordingly, as $\Delta \downarrow 0$,

$$\begin{aligned} w(t,x) = &\left(1 - \Delta \int \alpha(t-\Delta,x,x')\,\mathrm{d}x'\right) w(t-\Delta,x) \\ &+ \Delta \int \alpha(t-\Delta,x',x)\,B(t-\Delta,x',x)\,w(t-\Delta,x')\,\mathrm{d}x' + o(\Delta). \end{aligned}$$

In the limit, we obtain Eq. A4, the regular filter equation generated by α, with boost B and zero decay. □

When events are known to have occurred at particular times, it is of interest to integrate over those histories that include an event at each of these times. This leads to singular filter equations, as the next lemma shows. Before we state the lemma, some terminology is needed. Let $\mathbb{S}$ be the space of increasing, locally finite sequences in $\mathbb{R}_+$, with the topology induced by the Skorokhod metric and Lebesgue measure. For $t \in \mathbb{R}_+$ and $s \in \mathbb{S}$, let $s_t := s \cap [0,t]$. Thus if $s \in \mathbb{S}$ and $s_t = (\hat{s}_1, \dots, \hat{s}_K)$, then the infinitesimal element of Lebesgue measure at s_t is $\mathrm{d}s_t = \prod_{n=1}^{K} \mathrm{d}\hat{s}_n$.

Lemma A2. *Suppose that $B : \mathbb{R}_+ \times \mathbb{X}^2 \to \mathbb{R}_+$ is measurable and $\mathbf{V}_t$ is an $\mathbb{R}_+$-valued random process satisfying*

$$\mathbb{E}\left[\mathbf{V}_t \mid \mathbf{H}_t = \mathrm{H}_t\right] = \prod_{e \,\in\, \mathsf{ev}(\mathrm{H}_t)} B(e, \widetilde{\mathrm{X}}_e, \mathrm{X}_e).$$

Let λ_t be a family measures on $\mathbb{X} \times \mathbb{S}$ defined by

$$\lambda_t(\mathcal{E}, \mathcal{S}) = \mathbb{E}\left[\mathbf{V}_t \cdot \mathbb{1}\{\mathbf{X}_t \in \mathcal{E}\} \cdot \mathbb{1}\{\exists s \in \mathcal{S} \text{ s.t. } \mathsf{ev}(\mathbf{H}_t) \supseteq s_t\}\right],$$

whenever $\mathcal{E} \subseteq \mathbb{X}$ and $\mathcal{S} \subseteq \mathbb{S}$ are measurable. Let $w(t,x,s)$ be the density of this measure, i.e.,

$$\lambda_t(\mathrm{d}x\,\mathrm{d}s) = w(t,x,s)\,\mathrm{d}x\,\mathrm{d}s_t.$$

Then w satisfies

$$\frac{\partial w}{\partial t}(t,x,s) = \int w(t,x',s)\,\alpha(t,x',x)\,B(t,x',x)\,\mathrm{d}x' - \int w(t,x,s)\,\alpha(t,x,x')\,\mathrm{d}x', \qquad t \notin s, \tag{A5}$$

$$w(t,x,s) = \int \widetilde{w}(t,x',s)\,\alpha(t,x',x)\,B(t,x',x)\,\mathrm{d}x', \qquad t \in s. \tag{A6}$$

Proof. The proof proceeds by induction on the cardinality of s_t. The base case, for which $s_t = \varnothing$, follows immediately from Lemma A1. Assuming that it holds for $|s_t| < K$, one has only to verify Eq. A6. This can be accomplished by integrating Eq. 5 directly. □

Remark. In the same way that Eqs. 3 and 4 can be represented as a single equation by means of a Dirac delta notation, the Eqs. A5 and A6 can be collapsed into a more compact form if β is allowed to have atoms at a countable set of time-points and the boost B is adjusted appropriately.

Filter equations afford a convenient means of computing expectations and likelihoods for pure jump processes. This is facilitated by the following Lemma, the statement of which uses a one-sided Dirac delta function. Specifically, let $\delta(v,v')$ be the right-sided Dirac delta function satisfying $\delta(v,v') = 0$ for $v \neq v'$ and

$$\int_a^b f(v)\,\delta(v,v')\,\mathrm{d}v = f(v')\,\mathbb{1}\{v' \in [a,b)\},$$

whenever f is càdlàg and $-\infty \leqslant a < b \leqslant \infty$.

Lemma A3. *Eqs. A2 and A3 are satisfied by* $w(t,x) = \int_0^\infty v\,u(t,x,v)\,\mathrm{d}v$, *where* $u(t,x,v)$ *satisfies the KFE*

$$\begin{aligned}\frac{\partial u}{\partial t}(t,x,v) =&\frac{\partial}{\partial v}\left[\lambda(t,x)\,v\,u(t,x,v)\right] + \int_0^\infty\!\!\int u(t,x',v')\,\beta(t,x',x)\,\delta\big(v,B(t,x',x)\,v'\big)\,\mathrm{d}x'\,\mathrm{d}v'\\ &-\int_0^\infty\!\!\int u(t,x,v)\,\beta(t,x,x')\,\delta\big(v',B(t,x,x')\,v\big)\,\mathrm{d}x'\,\mathrm{d}v', && t\notin S,\\ u(t,x,v)\,\mathrm{d}x =& \int_0^\infty\!\!\int \tilde{u}(t,x',v')\,\pi(t,x',\mathrm{d}x)\,\delta\big(v,A(t,x')\,B(t,x',x)\,v'\big)\,\mathrm{d}x'\,\mathrm{d}v', && t\in S.\end{aligned}\tag{A7}$$

Here, $A(t,x) \coloneqq \int \beta(t,x,x')\,\mathrm{d}x'$ *and* $\pi(t,x,\mathrm{d}x') \coloneqq \beta(t,x,x')\,\mathrm{d}x'/A(t,x)$.

Proof. For each $t\notin S$, we have

$$\begin{aligned}\frac{\partial w}{\partial t}(t,x) &= \int_0^\infty v\,\frac{\partial u}{\partial t}(t,x,v)\,\mathrm{d}v\\ &= \int_0^\infty\!\!\int\!\int_0^\infty v\,u(t,x',v')\,\beta(t,x',x)\,\delta\big(v,B(t,x',x)\,v'\big)\,\mathrm{d}v\,\mathrm{d}x'\,\mathrm{d}v'\\ &\qquad-\int_0^\infty\!\!\int\!\int_0^\infty v\,u(t,x,v)\,\beta(t,x,x')\,\delta\big(v',B(t,x,x')\,v\big)\,\mathrm{d}v\,\mathrm{d}x'\,\mathrm{d}v'\\ &\qquad+\int_0^\infty v\,\tfrac{\partial}{\partial v}\left[\lambda(t,x)\,v\,u(t,x,v)\right]\,\mathrm{d}v.\end{aligned}$$

Here, the non-explosivity assumption guarantees that we can differentiate under the integral sign and exchange the order of integration. Moreover, it ensures that $u\to 0$ as $v\to\infty$. Hence, by evaluating the first integral with respect to v, the second with respect to v', and the third by parts, we obtain

$$\begin{aligned}\frac{\partial w}{\partial t}(t,x) =& \int v'\,u(t,x',v')\,\beta(t,x',x)\,B(t,x',x)\,\mathrm{d}v'\,\mathrm{d}x' - \int v\,u(t,x,v)\,\beta(t,x,x')\,\mathrm{d}v\,\mathrm{d}x'\\ &-\lambda(t,x)\int v\,u(t,x,v)\,\mathrm{d}v,\end{aligned}$$

which is simplified to obtain Eq. A2. Similarly, at each $t\in S$, we have

$$\begin{aligned}w(t,x)\,\mathrm{d}x &= \int_0^\infty\!\!\int\!\int_0^\infty v\,\tilde{u}(t,x',v')\,\pi(t,x',\mathrm{d}x)\,\delta\big(v,A(t,x')\,B(t,x',x)\,v'\big)\,\mathrm{d}x'\,\mathrm{d}v'\,\mathrm{d}v\\ &= \int_0^\infty\!\!\int v'\,\tilde{u}(t,x',v')\,\pi(t,x',\mathrm{d}x)\,A(t,x')\,B(t,x',x)\,\mathrm{d}x'\,\mathrm{d}v'\\ &= \int \tilde{w}(t,x')\,\beta(t,x',x)\,B(t,x',x)\,\mathrm{d}x'\,\mathrm{d}x.\end{aligned}$$

which is equivalent to Eq. A3 □

Remark. Eqs. A7 are recognizable as the KFE of a certain process $(\mathbf{X}_t,\mathbf{V}_t)$. In particular, the driver $\mathbf{X}_t$ has KFE Eq. A1. $\mathbf{V}_t$ is *directed* by $\mathbf{X}_t$ in the sense that $\mathbf{V}$ has jumps wherever $\mathbf{X}$ does: when $\mathbf{X}$ jumps at time t from x to x', $\mathbf{V}$ jumps by the multiplicative factor $B(t,x,x')\geqslant 0$. Between jumps, $\mathbf{V}_t$ decays deterministically and exponentially at rate $\lambda(t,x)$. At the known times in S, $\mathbf{X}$ jumps according to the probability kernel π and, $\mathbf{V}$ jumps by the factor $A(t,x)\,B(t,x,x')$. If we view $\mathbf{V}_t$ as a weight, then Lemma A3 tells us how the $\mathbf{V}_t$-weighted average of $\mathbf{X}_t$ evolves in time: this average is simply $\int w(t,x)\,\mathrm{d}x$. Thus, Lemma A3 shows how to integrate Eqs. A5 and A6 in the Monte Carlo sense.

Appendix B. Examples.

B.1. SIRS model. King et al. (2022) worked out formulas for the exact likelihood of a genealogy induced by an SIRS model. The theory developed in this paper applies, but since there is only one deme in this model, this is a simple case. Its state vector is $x = (S,I,R)$ and its KFE is

$$\begin{aligned}\frac{\partial v}{\partial t}(S,I,R) =& \frac{\beta\,(S+1)\,(I-1)}{N}\,v(t,S+1,I-1,R) - \frac{\beta\,S\,I}{N}\,v(t,S,I,R)\\ &+\gamma\,(I+1)\,v(t,S,I+1,R-1) - \gamma\,I\,v(t,S,I,R)\\ &+\omega\,(R+1)\,v(t,S-1,I,R+1) - \omega\,R\,v(t,S,I,R).\end{aligned}$$

Here $N = S + I + R$ is the host population size. Note that, though the theory allows for time-dependent event rates, this example is time-homogeneous. This model has one deme and occupancy function $n(x) = I$. There are four kinds of jumps: transmission, recovery, waning of immunity, and sampling. Accordingly, the marks are $\mathbb{U} = \{\mathsf{Trans}, \mathsf{Recov}, \mathsf{Wane}, \mathsf{Sample}\}$. Table B1 gives α_u, r_u, and the event type for each of these marks.

TABLE B1. Elements of the SIRS model pertinent to the genealogy process. The table shows the rate (α_u), jump ($x \mapsto x'$), production (r_u), and event type for each of the model's four marks (u).

u	α_u	$x \mapsto x'$	r_u	Event type
Trans	$\frac{\beta SI}{N}$	$(S, I) \mapsto (S-1, I+1)$	2	pure birth
Recov	γI	$(I, R) \mapsto (I-1, R+1)$	0	pure death
Wane	ωR	$(S, R) \mapsto (S+1, R-1)$	0	neutral
Sample	ψI	$x \mapsto x$	1	pure sample

Given an obscured genealogy Z, let $\mathsf{ev}(Z) = B \cup S_0 \cup S_1$, where B is the set of branch-times, and S_0, S_1 are the sets of sample-times with saturations 0 and 1, respectively. Since there is only one deme, paintings of Z can differ only in number and position of inline, internal nodes along branches. Each of these can only correspond to $u = \mathsf{Trans}$ with $s = 1$. For $t \notin \mathsf{ev}(Z)$, we can take the importance sampling distribution to be

$$\pi_u = \begin{cases} c, & u = \mathsf{Trans}, s = 0, t \notin \mathsf{ev}(Z), \\ \frac{1-c}{\ell}, & u = \mathsf{Trans}, s = 1, t \notin \mathsf{ev}(Z), \\ 0, & \text{otherwise.} \end{cases}$$

Here c is an arbitrary probability that does not affect the computation. The relevant binomial ratios are

$$\begin{pmatrix} n & \ell \\ r & s \end{pmatrix} = \begin{cases} \frac{(I-\ell)\,(I-\ell-1)}{I\,(I-1)}, & u = \mathsf{Trans}, s = 0, \\ \frac{2\,(I-\ell)}{I\,(I-1)}, & u = \mathsf{Trans}, s = 1, \\ \frac{2}{I\,(I-1)}, & u = \mathsf{Trans}, s = 2, \\ \frac{I-\ell}{I}, & u = \mathsf{Sample}, s = 0, \\ \frac{1}{I}, & u = \mathsf{Sample}, s = 1. \end{cases}$$

This leads, for $t \notin \mathsf{ev}(Z)$, to the following regular part of the filter equation:

$$\begin{aligned} \frac{\partial w}{\partial t} =& \frac{\beta\,(S+1)\,(I-1)}{N} \left(1 - \frac{\binom{\ell(t)}{2}}{\binom{I}{2}}\right) w(t, S+1, I-1, R) - \frac{\beta\,S\,I}{N}\,w(t, S, I, R) \\ &+ \gamma\,(I+1)\,w(t, S, I+1, R-1) - \gamma\,I\,w(t, S, I, R) \\ &+ \omega\,(R+1)\,w(t, S-1, I, R+1) - \omega\,R\,w(t, S, I, R) \\ &- \psi\,I\,w(t, S, I, R). \end{aligned}$$

Here, we have summed over the various paintings for $u = \mathsf{Trans}$, $s < 2$. Note the presence of the decay term proportional to ψ. At event-times $t \in \mathsf{ev}(Z)$, the singular part of the filter equation reads

$$w(t, S, I, R) = \begin{cases} \widetilde{w}(t, S+1, I-1, R)\,\frac{2\,\beta\,(S+1)}{N\,I}, & t \in B, \\ \widetilde{w}(t, S, I, R)\,\psi\,(I - \ell(t)), & t \in S_0, \\ \widetilde{w}(t, S, I, R)\,\psi, & t \in S_1. \end{cases}$$

Finally, note that $w(t, S, I, R) = 0$ for all $I < \ell(t)$.

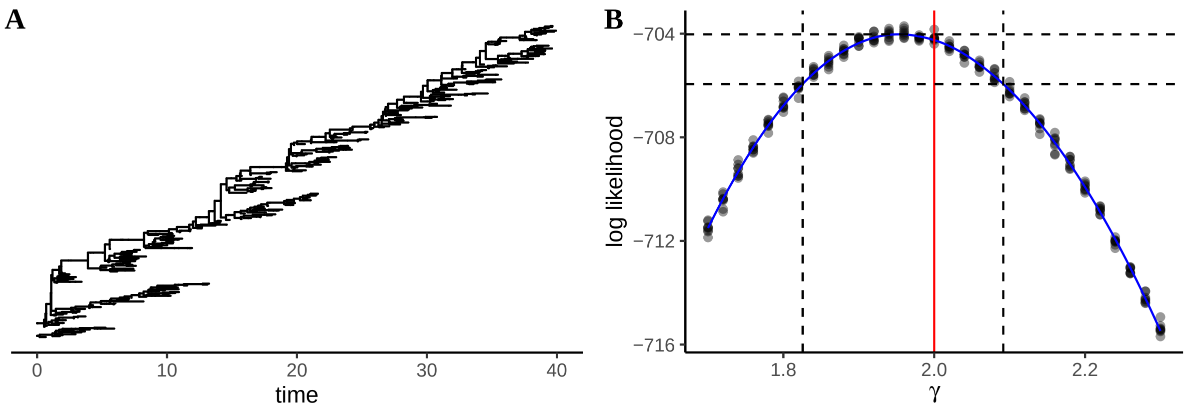

FIGURE B1. Likelihood computation for the SIRS model by Sequential Monte Carlo. (A) A simulated genealogy for $\beta = 4$, $\gamma = 2$, $\omega = 1$, $\psi = 1$, $(S_0, I_0, R_0) = (97, 3, 0)$. (B) A slice through the likelihood surface at the true parameters in the γ-direction. Each point is a distinct Monte Carlo estimate. The blue curve is a LOESS smooth; the dashed lines bound the Monte Carlo-adjusted 95% confidence interval (Ionides et al., 2017).

B.2. SEIRS model. A simple, yet interesting, model with more than one deme is the SEIRS model (Fig. 1A). The state space is $\mathbb{R}^4_+$, with the state $x = (S, E, I, R)$ defined by the numbers of hosts in each of the four compartments. The KFE for the population process is

$$\begin{aligned}\frac{\partial v}{\partial t}(t, S, E, I, R) = &\frac{\beta\,(S+1)\,I}{N}\,v(t, S+1, E-1, I, R) - \frac{\beta\,S\,I}{N}\,v(t, S, E, I, R)\\ &+ \sigma\,(E+1)\,v(t, S, E+1, I-1, R) - \sigma\,E\,v(t, S, E, I, R)\\ &+ \gamma\,(I+1)\,v(t, S, E, I+1, R-1) - \gamma\,I\,v(t, S, E, I, R)\\ &+ \omega\,(R+1)\,v(t, S-1, E, I, R+1) - \omega\,R\,v(t, S, E, I, R),\end{aligned}$$

where $N = S + E + I + R$ is the total population size. Note that the terms associated with sampling cancel each other in the KFE, since, in this model, sampling has no effect on the state.

This model has two demes: $\mathbb{D} = \{\mathsf{E}, \mathsf{I}\}$. Its deme occupancy function is $n(x) = (E, I)$. There are five kinds of jumps: transmission, progression, recovery, waning of immunity, and sampling. The corresponding marks are $\mathbb{U} = \{\mathsf{Trans}, \mathsf{Prog}, \mathsf{Recov}, \mathsf{Wane}, \mathsf{Sample}\}$. Table B2 gives α_u, r_u, and the event type for each of these marks.

TABLE B2. Elements of the SEIRS model pertinent to the genealogy process. The table shows the rate (α_u), jump ($x \mapsto x'$), production (r_u), and event type for each of the model's five marks (u).

u	α_u	$x \mapsto x'$	r_u	Event type
Trans	$\frac{\beta S I}{N}$	$(S, E) \mapsto (S-1, E+1)$	$(1, 1)$	pure birth
Prog	σE	$(E, I) \mapsto (E-1, I+1)$	$(0, 1)$	pure migration
Recov	γI	$(I, R) \mapsto (I-1, R+1)$	$(0, 0)$	pure death
Wane	ωR	$(S, R) \mapsto (S+1, R-1)$	$(0, 0)$	neutral
Sample	ψI	$x \mapsto x$	$(0, 1)$	pure sample

The filter equation corresponding to the scheme of Table B3 is presented in Box 1. Some numerical results are presented in Fig. B2.

TABLE B3. Elements of a scheme for numerically computing the likelihood under the SEIRS model. The regular portion of the filter equation holds in between genealogical events (i.e., for $t \notin B \cup S_0 \cup S_1$); the singular portion describes the effect of these events ($t \in B \cup S_0 \cup S_1$). Each line corresponds to a potential event, but only those for which $Q = 1$ appear in the equation. An event with mark u and saturation s has the boost given by the binomial ratio shown (third column). The $y \mapsto y'$ column depicts the proposed painting schematically, and π is the probability of that proposal. Blue is used for the E deme and yellow for the I deme. For each line, the filter equation contains m terms. An asterisk ($*$) stands for cases not explicitly mentioned.

	u	s	Q	$\begin{pmatrix} n & \ell \\ r & s \end{pmatrix}$	$y \mapsto y'$	π	m	Line
$t \notin B \cup S_0 \cup S_1$	Trans	$(0,0)$	1	$\left(\frac{E-\ell_E}{E}\right)\left(\frac{I-\ell_I}{I}\right)$	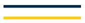	$\frac{I-\ell_I}{I}$	1	1
		$(1,0)$	1	$\frac{I-\ell_I}{EI}$	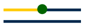	$\frac{1}{2I}$	ℓ_I	2
		$(0,1)$	1	$\frac{E-\ell_E}{EI}$	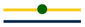	$\frac{1}{2I}$	ℓ_I	3
		$(1,1)$	0				0	4
	Prog	$(0,0)$	1	$\frac{I-\ell_I}{I}\,\mathbb{1}\{E \geqslant \ell_E\}$	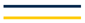	$\frac{E-\ell_E}{E}\,\mathbb{1}\{E > \ell_E\}$	1	5
		$(0,1)$	1	$\frac{1}{I}\,\mathbb{1}\{E \geqslant \ell_E\}$	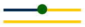	$\frac{1}{E}\,\mathbb{1}\{E > \ell_E\}$	ℓ_E	6
	Recov	$(0,0)$	1	$\mathbb{1}\{I \geqslant \ell_I\}$	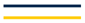	$\mathbb{1}\{I > \ell_I\}$	1	7
	Wane	$(0,0)$	1	1	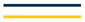	1	1	8
	Sample	$*$	0				0	9
$t \in B$	Trans	$(1,1)$	1	$\frac{1}{EI}$	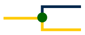	$\frac{1}{2}$	1	10
			1	$\frac{1}{EI}$	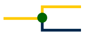	$\frac{1}{2}$	1	11
			0		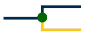		0	12
			0		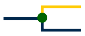		0	13
	$*$	$*$	0				0	14
$t \in S_0$	Sample	$(0,0)$	1	$\frac{I-\ell_I}{I}$	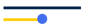	1	1	15
			0		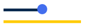		0	16
	$*$	$*$	0				0	17
$t \in S_1$	Sample	$(0,1)$	1	$\frac{1}{I}$	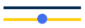	1	1	18
			0		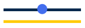		0	19
	$*$	$*$	0				0	20

Box 1: Filter equation for the SEIRS model

As previously, given an obscured genealogy, let B be the set of its branch times, S_0 be the set of tip-sample times, and S_1 be the set of inline sample times. Then for $t \notin B \cup S_0 \cup S_1$, the filter equation reads:

$$\text{(B8)}\quad \begin{aligned}\frac{\partial w}{\partial t}(t,S,E,I,R,y) = {} & \frac{\beta\,(S+1)\,I}{N}\left(1-\frac{\ell_E}{E}\right)\left(1-\frac{\ell_I}{I}\right) w(t,S+1,E-1,I,R,y) \\ & + \sum_{k=1}^{\ell_I} \frac{\beta\,(S+1)\,I}{N}\,\frac{1}{E}\left(1-\frac{\ell_I}{I}\right) w(t,S+1,E-1,I,R,\tilde{y}_2^k) \\ & + \sum_{k=1}^{\ell_I} \frac{\beta\,(S+1)\,I}{N}\,\frac{1}{I}\left(1-\frac{\ell_E}{E}\right) w(t,S+1,E-1,I,R,\tilde{y}_3^k) - \frac{\beta\,S\,I}{N}\,w(t,S,E,I,R,y) \\ & + \sigma\,(E+1)\,\mathbb{1}\{E \geqslant \ell_E\}\left(1-\frac{\ell_I}{I}\right) w(t,S,E+1,I-1,R,y) \\ & + \sum_{k=1}^{\ell_E} \sigma\,(E+1)\,\mathbb{1}\{E \geqslant \ell_E\}\,\frac{1}{I}\,w(t,S,E+1,I-1,R,\tilde{y}_6^k) - \sigma\,E\,w(t,S,E,I,R,y) \\ & + \gamma\,(I+1)\,\mathbb{1}\{I \geqslant \ell_I\}\,w(t,S,E,I+1,R-1,y) - \gamma\,I\,w(t,S,E,I,R,y) \\ & + \omega\,(R+1)\,w(t,S-1,E,I,R+1,y) - \omega\,R\,w(t,S,E,I,R,y) - \psi\,I\,w(t,S,E,I,R,y).\end{aligned}$$

Here, $\tilde{y}_j^k$ refers to the coloring of the tree immediately preceding the proposal indicated on line j of Table B3. The integer k specifies the particular branch on which the change occurs.
The singular portion of the filter equation has one component for each distinct type of genealogical event:

$$\text{(B9)}\quad w(t,S,E,I,R,y) = \begin{cases} \frac{\beta\,(S+1)\,I}{N}\,\frac{1}{EI}\,\tilde{w}(t,S+1,E-1,I,R,\tilde{y}_{10}) \\ \qquad + \frac{\beta\,(S+1)\,I}{N}\,\frac{1}{EI}\,\tilde{w}(t,S+1,E-1,I,R,\tilde{y}_{11}), & t \in B, \\ \psi\;(I-\ell_I)\;\tilde{w}(t,S,E,I,R,\tilde{y}_{15}), & t \in S_0, \\ \psi\,\tilde{w}(t,S,E,I,R,\tilde{y}_{18}), & t \in S_1. \end{cases}$$

In addition to Eqs. B8 and B9, the quantity w should satisfy the condition $w(t,S,E,I,R,y) = 0$ whenever $E < \ell_E$ or $I < \ell_I$.
A variety of importance-sampling kernels are permissible under the terms of Theorem 4. With a particular choice of importance-sampling kernel, the filter equation uniquely specifies a Sequential Monte Carlo algorithm for estimating the likelihood. The choices made in Table B3 underlie the results displayed in Fig. B2.

B.3. Two-strain competition model. A simple model for the competition of two strains for susceptible hosts is depicted in Fig. 1B. This example will be included in a forthcoming draft.

B.4. Superspreading model. Fig. 1D depicts a model of superspreading. This example will be included in a forthcoming draft.

B.5. Linear birth-death model. In this model, the state variable is the size, N_t, of a population at time t. All individuals face the same per-capita birth and death rates, which are λ and μ, respectively. The KFE is

$$\frac{\partial v}{\partial t} = \lambda\,(n-1)\,v(t,n-1) - \lambda\,n\,v(t,n) + \mu\,(n+1)\,v(t,n+1) - \mu\,n\,v(t,n)$$

Stadler (2010) considered the case where samples are taken through time at a uniform per-capita rate ψ. In this case, since there is only one deme, in the filter equation, w can be taken to be independent of y. If B is the set of branch-times and S_0, S_1 are the sets of terminal and inline samples, respectively, then the regular part of the filter equation is

$$\text{(B10)}\quad \begin{aligned}\frac{\partial w}{\partial t}(t,n) = {} & \lambda\,(n-1)\left(1-\frac{\binom{\ell(t)}{2}}{\binom{n}{2}}\right) w(t,n-1) - \lambda\,n\,w(t,n) \\ & + \mu\,(n+1)\,w(t,n+1) - \mu\,n\,w(t,n) - \psi\,n\,w(t,n), \quad n \geqslant \ell(t), \quad t \notin B \cup S_0 \cup S_1\end{aligned}$$

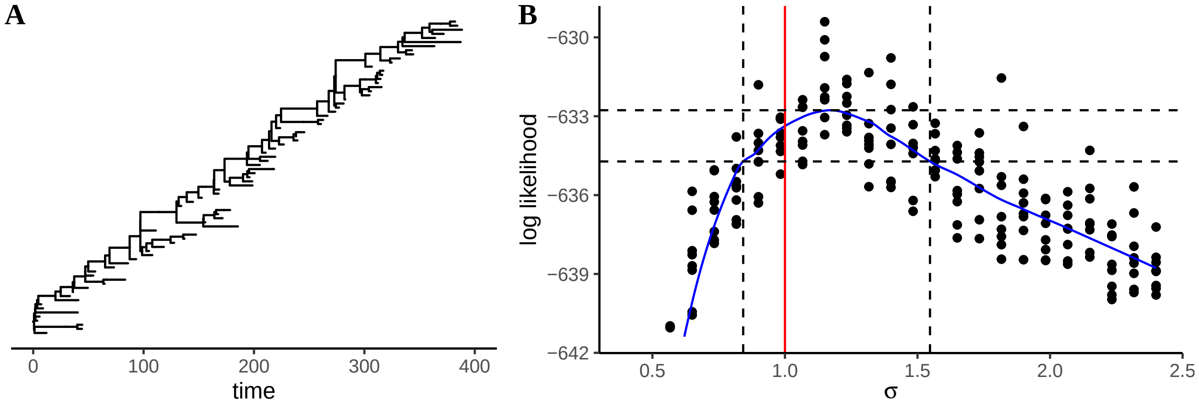

FIGURE B2. Likelihood computation for the SEIRS model by Sequential Monte Carlo, using the scheme of Box 1. (A) Simulated genealogy for $\beta = 3, \sigma = 1, \gamma = 0.5, \psi = 0.02, \omega = 0.08$, $(S_0, E_0, I_0, R_0) = (70, 1, 0, 50)$. (B) Likelihood slice in the σ-direction. Each point represents the estimate of an independent Sequential Monte Carlo computation. The blue curve shows a LOESS smooth; the dashed vertical lines enclose the Monte Carlo-adjusted 95% confidence interval (Ionides et al., 2017).

and the singular part is

$$w(t,n) = \frac{\lambda\,(n-1)}{\binom{n}{2}}\,\tilde{w}(t,n-1), \quad t \in B,$$

(B11)

$$w(t,n) = \psi\, n\left(1 - \frac{\ell(t)}{n}\right)\tilde{w}(t,n), \quad t \in S_0, \qquad w(t,n) = \psi\,\tilde{w}(t,n), \quad t \in S_1.$$

Eqs. B10 and B11 are supplemented by the ancillary condition $w(t,n) = 0$ for $n < \ell(t)$.

TABLE B4. Elements of the linear birth-death-sampling model pertinent to the genealogy process.

u	α_u	r_u	Event type
Birth	λN	2	pure birth
Death	γN	0	pure death
Sample	ψN	1	pure sample

B.6. Moran model and the Kingman coalescent. In the Moran model, events occur according to a rate-μ Poisson process. At each event, a compound birth-death jump (cf. Fig. 4F) occurs so that the population size, n, remains constant. If we let X_t be the number of events that have occurred by time t, then X_t is a simple counting process, which we can use to define the state of the population process. Its KFE is then

$$\frac{\partial v}{\partial t} = \mu\,(x-1)\,v(t,x-1) - \mu\,x\,v(t,x), \qquad v(0,x) = \begin{cases} 1, & x = 0, \\ 0, & x > 0. \end{cases}$$

Since there is only a single deme, and since nothing depends on the state, in writing the corresponding filter equation, we can take w to be independent of both x and y.

In the classical case (Kingman, 1982a), m samples are taken simultaneously at a single time, T. Then, if B is the set of branch-times and $\ell(t)$ is the number of lineages in the genealogy at time t, the filter equation reads

(B12) $$w(0) = 1, \qquad \frac{\partial w}{\partial t} = \mu\,w(t)\left(1 - \frac{\binom{\ell(t)}{2}}{\binom{n}{2}}\right) - \mu\,w(t), \quad t \notin B, \qquad w(t) = \frac{\mu}{\binom{n}{2}}\,\tilde{w}(t), \quad t \in B.$$

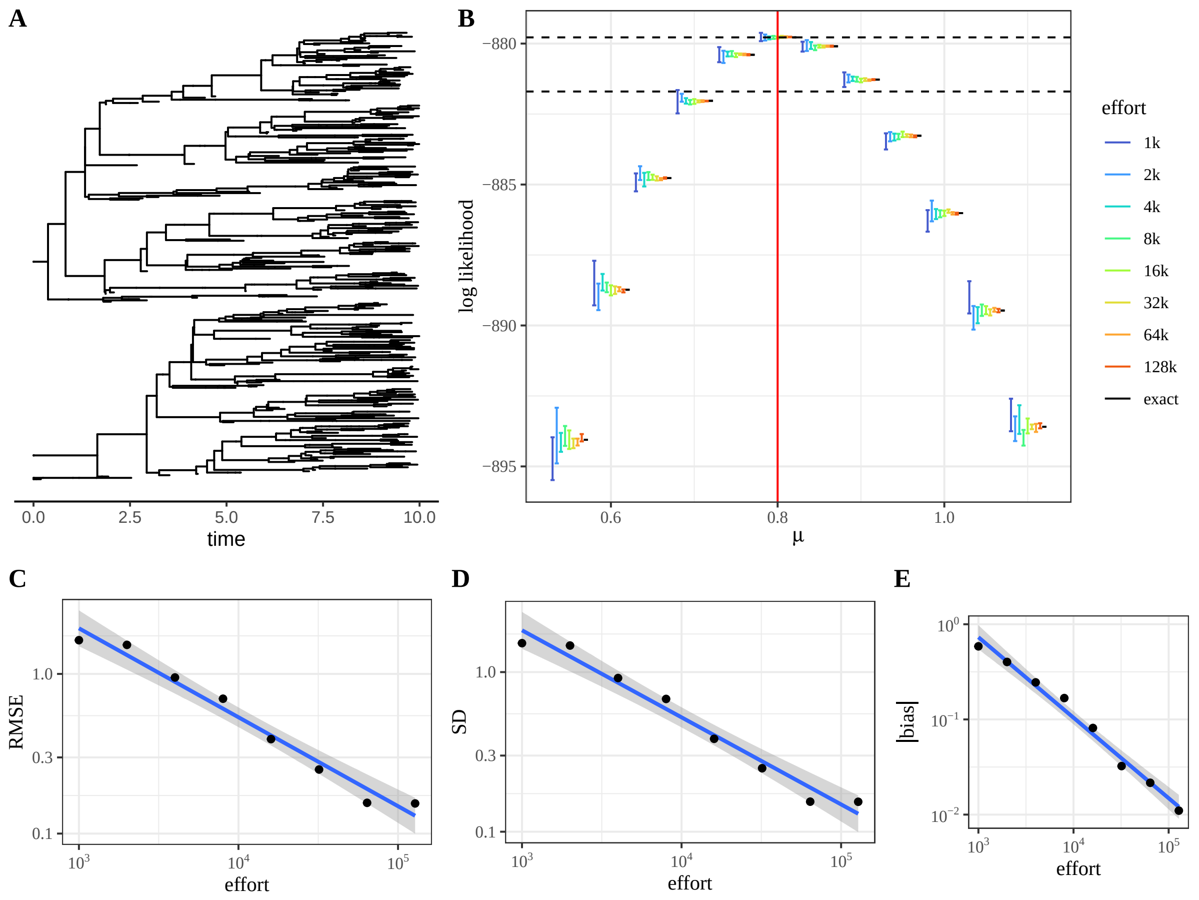

FIGURE B3. Likelihood computation for the constant-parameter, linear birth-death-sampling model, according to Theorem 4 via Sequential Monte Carlo. Panel (A) shows the genealogy, simulated for $\lambda = 1.2$, $\mu = 0.8$, $\psi = 1$, $N_0 = 5$. Panel (B) shows a likelihood slice, through the true parameters in the μ direction. As computational effort (i.e., number of particles) increases, the Monte Carlo estimates converge on the exact values, for which an explicit formula exists in this case. The dashed horizontal lines show the approximate maximized likelihood and the 95% critical value (under the likelihood-ratio test). Panels (C–E) are log-log plots that show how the root-mean-square error (RMSE), imprecision (SD), and bias decrease with effort. Note that the bias and the SD are roughly inversely proportional to the effort and its square-root, respectively, as expected.

Integrating Eqs. B12 and taking logarithms yields

$$\log w(T) = k \log \frac{\mu}{\binom{n}{2}} - \frac{\mu}{\binom{n}{2}} \sum_{i=m-k}^{m} \binom{i}{2} s_i, \tag{B13}$$

where $k = |B|$ is the number of branch-points in $[0, T]$ and the $s_i := \int \mathbb{1}\{\ell(t) = i\}\, dt$ are the durations of the *coalescent intervals*, i.e., intervals between successive branch-points. We recognize Eq. B13 as the expression for the Kingman (1982a) coalescent (e.g., Wakeley, 2009).

More generally, if in addition samples are taken according to a rate-ν Poisson process such that the set of sample-times in the genealogy is $S = S_0 \cup S_1$, where S_0, S_1 are the sets of times of terminal and inline samples,

respectively, then the filter equation reads

$$w(0) = 1, \qquad \frac{\partial w}{\partial t} = -\mu \, \frac{\binom{\ell(t)}{2}}{\binom{n}{2}} \, w(t), \quad t \notin S \cup B, \qquad w(t) = \frac{\mu}{\binom{n}{2}} \, \widetilde{w}(t), \quad t \in B,$$

(B14)

$$w(t) = \nu \left(1 - \frac{\ell(t)}{n}\right) \widetilde{w}(t), \quad t \in S_0, \qquad w(t) = \frac{\nu}{n} \, \widetilde{w}(t), \quad t \in S_1.$$

Integrating Eqs. B14 yields

$$\log w(T) - |S| \, \log \nu = \sum_{t \in S_0} \log \left(1 - \frac{\ell(t)}{n}\right) - |S_1| \, \log n + |B| \, \log \frac{\mu}{\binom{n}{2}} - \frac{\mu}{\binom{n}{2}} \sum_{i=1}^{\infty} \binom{i}{2} \, s_i. \tag{B15}$$

A. A. King, Department of Ecology & Evolutionary Biology, Center for the Study of Complex Systems, and Department of Mathematics, University of Michigan, Ann Arbor, MI 48109 USA *and* Santa Fe Institute, 1399 Hyde Park Road, Santa Fe, NM 87501 USA

Email address: kingaa@umich.edu

URL: https://kinglab.eeb.lsa.umich.edu/

Q.-Y. Lin, Theoretical Biology and Biophysics, Los Alamos National Laboratory, Los Alamos, NM 87545 USA

E. L. Ionides, Department of Statistics, University of Michigan, Ann Arbor, MI 48109 USA
